# CTCF mediates chromatin looping via N-terminal domain-dependent cohesin retention

**DOI:** 10.1073/pnas.1911708117

**Published:** 2020-01-14

**Authors:** Elena M. Pugacheva, Naoki Kubo, Dmitri Loukinov, Md Tajmul, Sungyun Kang, Alexander L. Kovalchuk, Alexander V. Strunnikov, Gabriel E. Zentner, Bing Ren, Victor V. Lobanenkov

**Affiliations:** ^a^Molecular Pathology Section, Laboratory of Immunogenetics, National Institute of Allergy and Infectious Diseases, National Institutes of Health, Bethesda, MD 20892;; ^b^Ludwig Institute for Cancer Research, University of California San Diego, La Jolla, CA 92093;; ^c^Department of Biology, Indiana University, Bloomington, IN 47405;; ^d^Molecular Epigenetics Laboratory, Guangzhou Institutes of Biomedicine and Health, Science Park, 510530 Guangzhou, China;; ^e^Indiana University Melvin and Bren Simon Cancer Center, Indiana University–Purdue University, Indianapolis, IN 46202;; ^f^Department of Cellular and Molecular Medicine, Center for Epigenomics, University of California San Diego School of Medicine, La Jolla, CA 92093-0653;; ^g^Moores Cancer Center and Institute of Genomic Medicine, University of California San Diego School of Medicine, La Jolla, CA 92093-0653

**Keywords:** CTCF, cohesin, BORIS, 3D genome organization

## Abstract

The DNA-binding protein CCCTC-binding factor (CTCF) and the cohesin complex function together to shape chromatin architecture in mammalian cells, but the molecular details of this process remain unclear. Here, we demonstrate that a 79-aa region within the CTCF N terminus is essential for cohesin positioning at CTCF binding sites and chromatin loop formation. However, the N terminus of CTCF fused to artificial zinc fingers was not sufficient to redirect cohesin to non-CTCF binding sites, indicating a lack of an autonomously functioning domain in CTCF responsible for cohesin positioning. BORIS (CTCFL), a germline-specific paralog of CTCF, was unable to anchor cohesin to CTCF DNA binding sites. Furthermore, CTCF–BORIS chimeric constructs provided evidence that, besides the N terminus of CTCF, the first two CTCF zinc fingers, and likely the 3D geometry of CTCF–DNA complexes, are also involved in cohesin retention. Based on this knowledge, we were able to convert BORIS into CTCF with respect to cohesin positioning, thus providing additional molecular details of the ability of CTCF to retain cohesin. Taken together, our data provide insight into the process by which DNA-bound CTCF constrains cohesin movement to shape spatiotemporal genome organization.

## Significance

The DNA-binding protein CCCTC-binding factor (CTCF) and the cohesin complex function together to establish chromatin loops and regulate gene expression in mammalian cells. It has been proposed that the cohesin complex moving bidirectionally along DNA extrudes the chromatin fiber and generates chromatin loops when it pauses at CTCF binding sites. To date, the mechanisms by which cohesin localizes at CTCF binding sites remain unclear. In the present study we define two short segments within the CTCF protein that are essential for localization of cohesin complexes at CTCF binding sites. Based on our data, we propose that the N-terminus of CTCF and 3D geometry of the CTCF–DNA complex act as a roadblock constraining cohesin movement and establishing long-range chromatin loops.

The 3D structures of chromosomes in vertebrate cells play a critical role in nearly all nuclear processes, including transcriptional control of gene expression, replication of DNA, repair of DNA damage, and splicing of messenger RNA (1–4). A key player in chromatin organization is the CCCTC-binding factor (CTCF), an 11-zinc finger (11ZF) protein (5, 6) that regulates formation of topologically associating domains and long-range chromatin loops in interphase nuclei (7–9). CTCF works together with cohesin, a multipolypeptide complex with an evolutionarily conserved role in sister chromatid cohesion during mitosis, to shape chromatin architecture (10–13). This process has been proposed to involve the loading of cohesin onto chromatin by factors such as NIPBL followed by ATP-dependent bidirectional movement of the cohesin complex and extrusion of the chromatin fiber (14–16). According to this model, bidirectional movement of the cohesin complex along the chromatin fiber can be stalled at CTCF sites, especially at a pair of sites in convergent orientation, resulting in the appearance of topologically associating domains and chromatin loops in a cell population (15, 17). While this model is supported by a substantial amount of experimental evidence (17–19), important questions remain. First, while it is well known that CTCF and cohesin are frequent partners in the formation of large-scale chromatin loops, genome-wide mapping of both factors has suggested that there are substantial fractions of cohesin binding sites not occupied by CTCF (cohesin non-CTCF [CNC]) in various mammalian cell lines (20–22). Based on these findings, it has been suggested that there is a significant CTCF-independent role for cohesin in shaping genome architecture (21, 23, 24). However, the proportion of CNC sites varies between cell types, and their implication for chromatin loop formation is largely unclear. Second, it is unclear how cohesin is retained at CTCF binding sites. Does CTCF directly recruit cohesin to chromatin via protein–protein interactions, or act to constrain cohesin movement via other means during loop extrusion?

Here, we sought to clarify the relationship between CTCF and cohesin during formation of chromatin loops. First, we show that more than 95% of CTCF sites are also bound by cohesin in multiple cell lines, implying a nearly universal role of CTCF in the retention of cohesin onto chromatin independent of the sequence or function of the CTCF binding site. Second, we demonstrate that the N terminus of CTCF is essential but not sufficient for cohesin retention at CTCF sites and chromatin loop formation. We further delineate a minimal 79-aa segment within the CTCF N terminus responsible for cohesin retention. Third, we also show that BORIS, a paralog of CTCF, is not able to retain cohesin. Using chimeric proteins constructed between CTCF and BORIS, we found that the first two ZF domains of CTCF are also involved in cohesin localization to CTCF binding sites, and together with the N terminus of CTCF, can confer the ability to anchor cohesin to BORIS. Taken together, our data provide insights into the mechanism by which the CTCF–DNA complex stalls cohesin movement to establish chromatin loops in mammalian cells.

## Results

### CTCF and NIBPL Are Sufficient to Explain the Genomic Distribution of Cohesin.

**Fig. 1.**
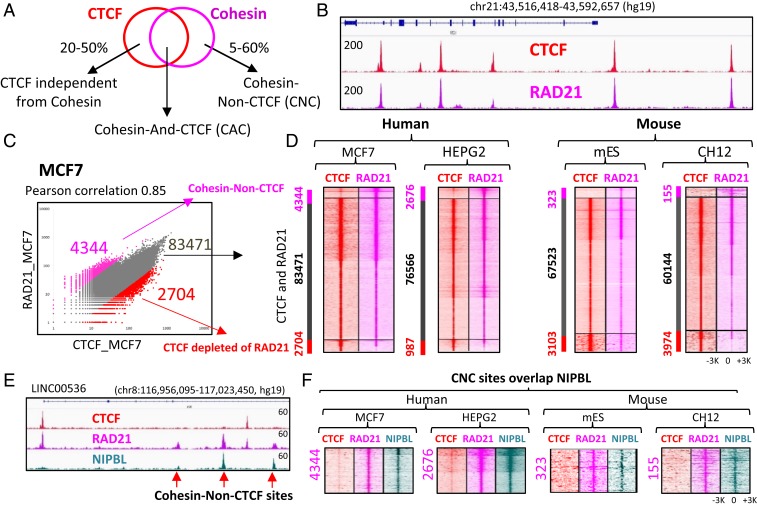
Cohesin occupancy is mainly explained by CTCF and NIPBL in diverse cell types. (*A*) Venn diagram representation of the inconsistent overlap of CTCF and cohesin ChIP-seq binding regions according to the current literature. (*B*) Genome browser view of CTCF and cohesin (RAD21) occupancy in MCF7 cells. Red and pink tracks display CTCF and RAD21 ChIP-seq signals, respectively. (*C*) Scatter plot of CTCF and RAD21 occupancy in MCF7 cells. CTCF and RAD21 ChIP-seq peaks were combined into a merged set of 90,519 sites and tag density was calculated at each binding region. (*D*) Heatmaps of CTCF (red) and RAD21 (pink) occupancy at genomic regions bound either by CTCF or RAD21 or both in four different cell lines. The first panel of heatmap (MCF7) corresponds to the scatter plot in *C*, with the connection between two panels shown by black arrows. Every heatmap is separated into three sections based on differential CTCF and RAD21 occupancy determined by scatter plots in *C*. (*E*) Genome browser view of CTCF, RAD21, and NIPBL occupancy in MCF7 cells showing that RAD21 sites depleted of CTCF are coincident with NIPBL binding sites, highlighted by red arrows. (*F*) Heatmaps showing that CNC sites are coincident with NIPBL binding (green) in four different cell lines. The RAD21 sites correspond to those determined in *D*.

While CTCF has been proposed to be the major factor required for cohesin anchoring on chromatin, there are conflicting reports as to the extent of overlap in their genomic distributions (10, 21, 22, 25, 26) (Fig. 1*A*). To resolve this issue, we performed chromatin immunoprecipitation-sequencing (ChIP-seq) analysis of CTCF and cohesin binding in several cancer cell lines. In the MCF7 human breast cancer cell line, where a large percentage of CTCF-independent cohesin binding sites were previously reported (22), we observed robust correspondence between CTCF and RAD21 (a subunit of cohesin) binding at the single-locus (Fig. 1*B*) and genome-wide scales, with 90.7% of RAD21 peaks overlapping CTCF binding sites (*SI Appendix*, Fig. S1*A*). While a computational method identified ∼25,000 more CTCF binding sites than RAD21 binding sites, an enrichment of RAD21 ChIP-seq signal was readily visible at these additional CTCF sites (*SI Appendix*, Fig. S1 *B* and *C*). We thus combined CTCF and RAD21 peaks into a composite set of binding sites and classified them as bound by both CTCF and RAD21 or bound by CTCF but not RAD21 or vice versa if the difference in tag density between the two factors was >threefold. The majority of sites was occupied by both CTCF and RAD21 (92.2%) (Fig. 1*C*), with only a minority of sites (4.8%) displaying significant RAD21 ChIP-seq signal with low levels of CTCF binding, or CTCF binding with little RAD21 ChIP-seq signal (3%) (Fig. 1 *B* and *C*). Similar observations were made in three additional cell lines, namely human hepatocellular carcinoma cells (HepG2), mouse embryonic stem cells (mESCs), and mouse B cell lymphoma cells (CH12), with cooccupancy of CTCF and RAD21 at a majority of sites (93.6 to 95.4%) observed in each case, and relatively low numbers of sites bound by CTCF but not RAD21 and vice versa (Fig. 1*D*). Between cell types, CTCF and cohesin (CAC) sites were the most reproducible, followed by CTCF sites without RAD21 enrichment, while there was very little overlap of CNC (*SI Appendix*, Fig. S1*D*).

We next set out to identify factors bound to CNC sites. Consistent with reports that the NIPBL protein promotes the loading of cohesin onto chromatin (27, 28), the CNC sites are enriched for NIPBL (Fig. 1*E*) in MCF7, HepG2, mESC, and CH12 cells, supporting a role for NIBPL in CTCF-independent cohesin loading (Fig. 1*F* and *SI Appendix*, Fig. S2). In contrast, ESR1, CEBPA, and OCT4, previously reported to promote cohesin retention in MCF7, HepG2, and mESC, respectively, showed insignificant occupancy at CNC sites (*SI Appendix*, Fig. S2).

We hypothesized that the discrepancies between this study and the previous reports of a large number of CNC sites might be due to the use of different antibodies against CTCF. We thus performed ChIP-seq experiments with a custom mixture of nine monoclonal antibodies against the CTCF N terminus and a custom mixture of seven commercial polyclonal antibodies against the CTCF C terminus. We also compared the CTCF binding sites with the RAD21 binding sites in the K562 cells using datasets generated by the ENCODE consortium (29). While the majority of RAD21 sites (88%) was occupied by CTCF in all six ChIP-seq experiments, only monoclonal antibodies against the N terminus of CTCF and a mixture of seven polyclonal CTCF antibodies were able to detect CTCF occupancy at the residual 12% of RAD21 peaks (*SI Appendix*, Fig. S3 *A* and *B*). The number of CTCF peaks detected with each antibody was also variable, ranging from 58,302 to 96,154 (*SI Appendix*, Fig. S3*C*). We also found that studies employing a single antibody against the CTCF C terminus did not detect a substantial number of sites identified using an N-terminal antibody in HepG2 cells (*SI Appendix*, Fig. S3 *D* and *E*). Thus, previous studies of CTCF and cohesin overlap may have been confounded by incomplete mapping of CTCF binding sites due to epitope masking.

### Cohesin Occupancy at CTCF Binding Sites Depends on CTCF.

**Fig. 2.**
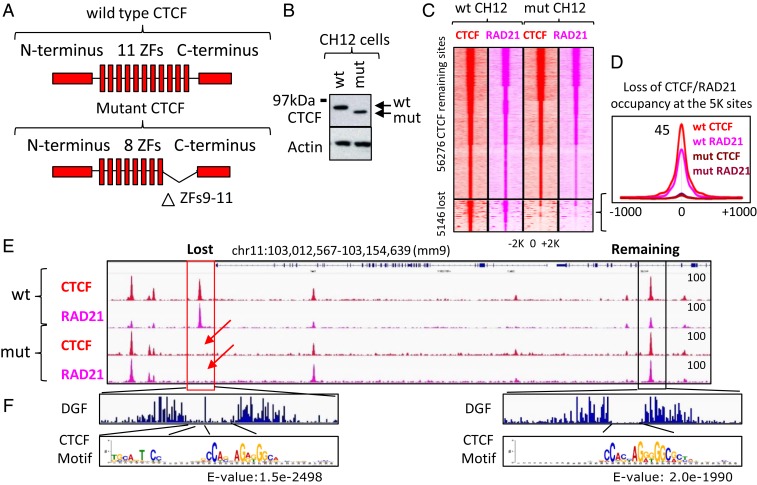
Loss of CTCF binding results in loss of cohesin occupancy. (*A*) Schematic representations of WT and mut CTCF proteins in WT and mut CH12 cells, respectively. The mutant CTCF has a homozygous deletion of ZFs 9 to 11. (*B*) Western blot of WT and mut CTCF proteins in CH12 cells. (*C*) Heatmap showing CTCF (red) and RAD21 (pink) occupancy in WT and mut CH12 cells at CTCF sites mapped in WT CH12 cells. The 5,146 CTCF sites lost CTCF occupancy in mut CH12 cells compared to WT CH12 cells. Loss of CTCF was accompanied by loss of cohesin occupancy. (*D*) Average profile of CTCF and RAD21 occupancy at the 5K lost CTCF binding sites from *C*. (*E*) Genome browser view of CTCF and RAD21 ChIP-seq data in WT and mut CH12 cells. Lost and preserved CTCF and RAD21 binding sites are highlighted by red and black boxes, respectively. Red arrows show loss of CTCF and RAD21 binding as a consequence of CTCF mutation. (*F*) DGF data under lost and preserved CTCF binding sites from *E*. Below the DGF plots are sequence logos from de novo motif analysis under the summit of 1,000 lost and preserved CTCF sites.

Our results thus far indicate that cohesin occupancy closely tracks CTCF binding genome-wide (Fig. 1). These data are wholly consistent with the results from the previous studies using CTCF auxin-inducible degron system and with CTCF knockout mouse fibroblasts where, in the absence of CTCF, cohesin is no longer able to occupy CTCF binding sites (30, 31). In order to experimentally test the hypothesis that cohesin occupancy at a given site is CTCF-dependent, we used a mouse CH12 B cell lymphoma cell line in which endogenous CTCF has been homozygously mutated (14). In this cell line, the first half of CTCF zinc finger 9 (ZF9) is fused to the second half of ZF11 and a BioTag coding sequence is inserted at the C terminus, thus resulting in a mutated (mut) form of CTCF with only the first eight ZFs being functional (Fig. 2*A* and *SI Appendix*, Fig. S4). Western blotting of WT and mut CH12 cells with antibodies raised against the N terminus of CTCF confirmed the presence of a homozygous mutation of CTCF, as well as the expression of both proteins at comparable levels (Fig. 2*B*). ChIP-seq mapping of CTCF and RAD21 occupancy in WT and mut CH12 cells showed that the deletion of the last three ZFs of CTCF resulted in either complete loss or in a dramatic decrease of CTCF occupancy at 5,146 binding sites (8%), while the remaining 56,276 loci were still occupied by the mut CTCF at levels comparable with the WT CTCF (Fig. 2 *C*–*E*). Notably, following either loss or reduction of CTCF occupancy, RAD21 occupancy was also proportionally diminished at these 5,146 (5K) sites (Fig. 2 *C*–*E*).

To investigate what sequences are associated with the lost and preserved CTCF sites, we performed de novo motif enrichment analysis using the sequence of corresponding peaks. Sites that lost CTCF binding in mut CH12 cells displayed enrichment of an upstream module of the CTCF motif (Fig. 2 *E* and *F*), consistent with the previous report that this sequence is bound by ZF9 to -11 of CTCF (32). Moreover, analysis of digital genomic footprinting (DGF) data (ENCODE) at the lost and remaining CTCF sites demonstrated a longer footprint under the lost CTCF peaks, in agreement with a presence of long CTCF motif under the same CTCF peaks (Fig. 2*F*). Beyond this difference, the 5K lost CTCF binding sites were very similar to all other CTCF binding sites mapped in WT CH12 cells in terms of genomic distribution, colocalization with other transcription factors, and chromatin modifications (*SI Appendix*, Fig. S5).

To determine whether loss of the 5K CTCF/cohesin binding sites affects gene expression, we performed RNA-sequencing (RNA-seq) experiments in WT and mut CH12 cells. We observed that 1,489 genes were deregulated (957 up-regulated and 532 down-regulated, *P* < 0.005) in mut CH12 cells compared to WT (*SI Appendix*, Fig. S6 *A* and *B*). The main pathways deregulated in mut CH12 cells were significantly associated with B cell biology (*SI Appendix*, Fig. S6*C*), which is related to the origin (B cell lymphoma) of these cells. Moreover, the functional annotation of deregulated genes showed a significant enrichment of genes involved in B cell development, function, and proliferation (for example, *Tnfsf13b*, *Tnfsf15*, *Tlr6*, *Aicda*, *Fas*, *Lck*, *Irf8*, *Fos*, *Tlr4*, and *Tnf*) (*SI Appendix*, Fig. S6*D*). These data confirm that the 5K lost CTCF sites are not associated with some specific genomic regions, but rather equally distributed in the genome. The majority (60%) of deregulated genes had a lost CTCF peak within 100 kb of the gene (*SI Appendix*, Fig. S6*B*), with a tendency for down-regulated genes to show a decrease of CTCF occupancy in the promoter region (*SI Appendix*, Fig. S6*E*). For example, *App*, the expression of which was previously shown to be positively regulated by CTCF (33), was severely down-regulated in mut CH12 cells and displayed a loss of CTCF occupancy at its CpG-rich promoter (*SI Appendix*, Fig. S6*F*).

In summary, the analysis of CTCF and cohesin occupancy in WT and mut CH12 cells confirms the prevalent dependence of cohesin occupancy on CTCF and reveals a valuable model to study the interaction of two architectural proteins in chromatin organization and gene expression in a relatively stable setting on a genome-wide scale.

### The N Terminus of CTCF Is Required for Cohesin Occupancy at CTCF Binding Sites.

While a nearly perfect correlation between CTCF and somatic cohesin occupancies is evident from our and other data, the mechanism by which cohesin localizes at CTCF binding sites remain obscures. One of the prevalent theories postulates that CTCF directly binds the SA2 subunit of cohesin via its C terminus to retain or stabilize cohesin positioning (34). The model is based on the immunoprecipitation of SA2 with a specific region (amino acids 575 to 611) of the CTCF C terminus and is indirectly supported by the loss of CTCF-mediated insulation upon expression of C-terminally truncated CTCF mutants (34). However, precise deletion of the same peptide sequence from the C terminus of CTCF does not disrupt the interaction of CTCF with cohesin, as shown in two independent studies (35, 36). Furthermore, a high-resolution analysis of ChIP-seq data based on the orientation of CTCF consensus sequences showed that cohesin occupancy at CTCF binding sites is usually shifted toward the N terminus rather than the C terminus of CTCF (26, 37).

**Fig. 3.**
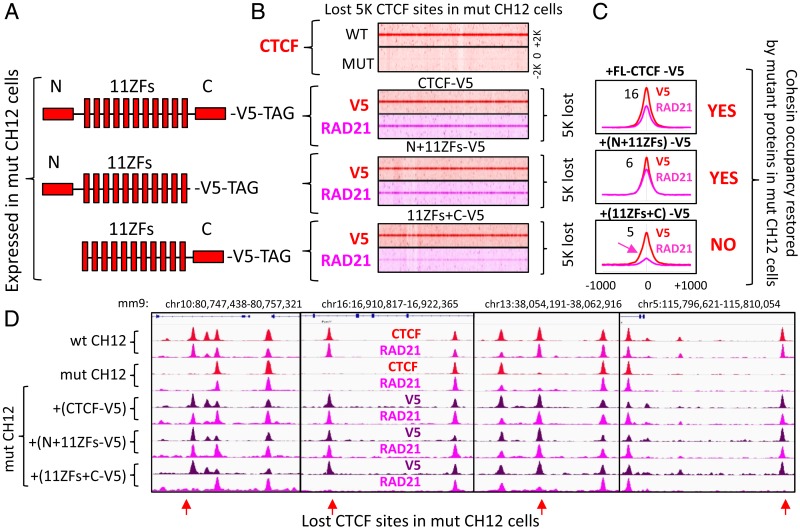
The CTCF N terminus is necessary for cohesin retention on DNA. (*A*) Schematic representation of the three CTCF constructs stably expressed in mut CH12 cells. (*B*) (*Top* heatmap) CTCF occupancy at the 5K lost CTCF binding sites from Fig. 2*C* in WT and mut CH12 cells. Below are three heatmaps of V5-tag (red) and RAD21 (pink) occupancy at the 5K lost CTCF sites in mut CH12 cells expressing the corresponding proteins from *A*. (*C*) Average profile of V5-tagged CTCF constructs and RAD21 occupancy at the 5K lost CTCF binding sites from *B*. The summary of cohesin occupancy induced by the expressed proteins is indicated by YES or NO. (*D*) Genome browser view of CTCF (red), V5 (purple), and RAD21 (pink) ChIP-seq data in WT and mut CH12 cells expressing the CTCF constructs from *A*. The lost CTCF binding sites are indicated by red arrows.

To map the CTCF domain responsible for cohesin binding in vivo, we employed the mut CH12 cell system described above (Fig. 2). As the 5K binding sites lost CTCF occupancy in mut CH12 cells due to the deletion of ZFs 9 to 11, reexpression of N- or C-terminally truncated CTCF mutants with the intact 11ZF DNA binding domain could potentially restore CTCF occupancy at the lost sites and simultaneously reveal which domain of CTCF protein is necessary for cohesin retention. First, we tested the capacity of an exogenous full-length CTCF (FL-CTCF) to restore CTCF occupancy at the lost sites by expressing V5-tagged WT CTCF and performing ChIP-seq with both V5 and CTCF antibodies. The ectopic V5-tagged CTCF, expressed at a level comparable to mut CTCF (*SI Appendix*, Fig. S7*A*), recapitulated the CTCF binding pattern observed for WT CH12 cells, including the reestablishment of occupancy at the 5K lost CTCF binding sites in the mut CH12 cells (Fig. 3 *A* and *B* and *SI Appendix*, Fig. S8*A*). Of note, the ectopic expression of CTCF does not affect proliferation of mut CH12 cells (*SI Appendix*, Fig. S7*B*). Furthermore, the restoration of CTCF binding at these 5K sites was accompanied by increased RAD21 occupancy (Fig. 3 *B* and *C*). A more detailed analysis of V5-tag density enrichment at the lost CTCF sites showed that 188 sites failed to reestablish CTCF binding (*SI Appendix*, Fig. S8*B*). A telling example of such a “permanently lost” CTCF site was within the CpG rich *App* promoter, which was accompanied by silencing of *App* expression in mut CH12 cells (*SI Appendix*, Figs. S6*F* and S8*D*). This could be potentially attributed to alterations in chromatin state. Indeed, it has been shown previously that CTCF binding protects certain sequences from the repressive histone marks and CpG methylation (38).

Having validated our ectopic FL-CTCF system in the mutant cells, we proceeded to investigate the contribution of the N- and C-terminal regions of CTCF to the stabilization of cohesin positioning (Fig. 3*A*). The N- or C-terminally truncated mutants of CTCF with an intact 11ZF DNA binding domain were stably expressed in mut CH12 cells and analyzed by ChIP-seq with both CTCF and V5 tag antibodies (*SI Appendix*, Figs. S7*A* and S8*C*). In the case of either N-terminus or C-terminus truncations, we reciprocally used a C terminal- or N terminal-specific anti-CTCF antibody. The binding pattern of truncated mutants in mut CH12 cells generally reproduced that of FL-CTCF (*SI Appendix*, Fig. S8*C*), confirming that only the 11ZF DNA-binding domain of CTCF is involved in binding to the genome. However, CTCF lacking its N terminus was unable to restore RAD21 occupancy at the ∼5K sites lost in mut CH12 cells, while C-terminally truncated CTCF effectively replenished RAD21 at these loci (Fig. 3 *B*–*D*). Therefore, it is apparent that the N terminus of CTCF is critical for cohesin retention at the specific position on DNA, in contrast to the published model of C terminus-dependent recruitment.

### The N but Not the C Terminus of CTCF Is Necessary for Chromatin Loop Formation.

**Fig. 4.**
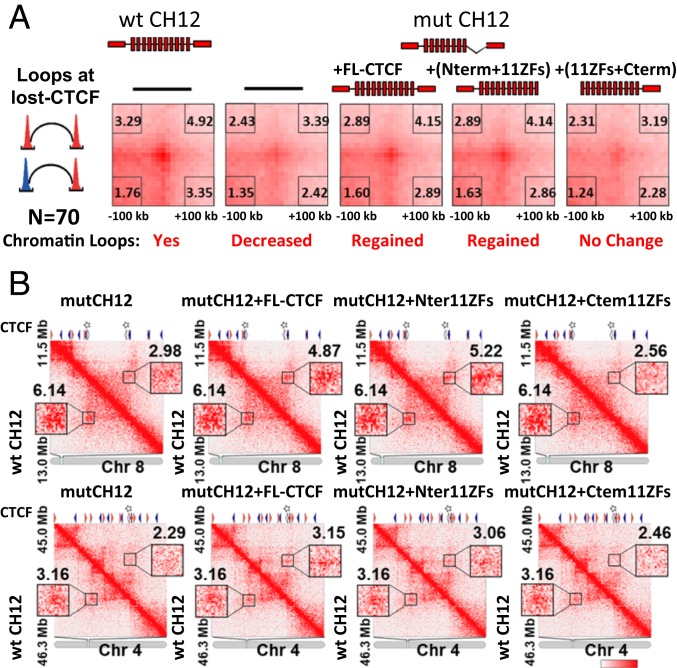
The CTCF N terminus is necessary for chromatin loop formation. (*A*) Chromatin loops were analyzed by Hi-C in the five types of cells (WT CH12, mut CH12, and mut CH12 cells stably expressing either of three CTCF constructs: FL-CTCF, N terminus+11ZFs, and C terminus+11ZFs). APA was performed on chromatin loops affected in mut CH12 cells compared to WT CH12 cells. Seventy loops that associated with at least one lost CTCF site (>300-kb looping range) were selected for the analysis in the five types of cells. Peak signal strength is indicated as the ratio of the central pixel to the lower left pixels in each heatmap. (*B*) Hi-C contact matrices showing examples of chromatin loops at the lost CTCF sites. Lower triangles show Hi-C signal from WT CH12 and upper triangles show Hi-C data from cells expressing the indicated form of exogenous CTCF. The orientation of CTCF sites is shown by orange (+ strand) or blue (− strand) triangles and the lost CTCF sites are indicated by stars.

According to a current hypothetical model, cohesin extrudes chromatin loops until it encounters a CTCF dimer formed by two molecules of CTCF bound to the convergent binding sites (17, 39). However, it is not known which domains of CTCF are involved in chromatin loop formation. As we have shown here, the N terminus of CTCF is necessary for cohesin retention at CTCF target sites (Fig. 3). In addition, the C terminus has been shown to be required for CTCF dimerization (36, 40). This raises the interesting question of whether both CTCF domains are necessary for anchoring of chromatin loops. To address this question, we first analyzed the chromatin contacts at the 5K lost CTCF sites by performing Hi-C experiments on WT and mut CH12 cells. To this end, we selected 70 Hi-C chromatin loops that overlapped with the 5K lost CTCF sites at one or both anchors (see the selection process of 70 loops in *SI Appendix*, Fig. S9). Next, aggregate peak analysis (APA) demonstrated decreased signal at these chromatin loops in mut CH12 cells compared to WT CH12 cells (Fig. 4*A*), confirming that the loss of CTCF and cohesin binding leads to the loss of chromatin contacts. Subsequently, we performed Hi-C analysis in mut CH12 cells stably expressing either FL-CTCF, C-terminally truncated (N terminus+11ZFs), or N-terminally truncated (11ZFs+C terminus) CTCF proteins. APA on the above 70 chromatin loops showed that long-range looping was generally restored by the expression of either FL-CTCF or N terminus+11ZFs, but not by the 11ZFs+C terminus CTCF (Fig. 4*A*). A sample of individual chromatin loops overlapping with one or two lost CTCF sites also showed clear changes in peak signal (Fig. 4*B*). These results strongly suggest that the N terminus of CTCF, but not its C terminus, is necessary for chromatin loop formation.

We next asked whether the CTCF sites without cohesin enrichment are involved in chromatin loop formation. To address this question, we analyzed CTCF sites either enriched or depleted of cohesin (Fig. 1*D*) with respect to their involvement in chromatin loop anchoring. We divided the CTCF sites into two equally numbered groups, highly and lowly enriched with cohesin, and compared them to previously reported chromatin loops mapped by chromatin interaction analysis with paired-end tag and Hi-C in WT CH12 cells (14). Only the CTCF sites highly enriched with cohesin were present at the anchors of loops mapped by both methods (*SI Appendix*, Fig. S10). This suggests that cohesin occupancy at CTCF binding sites is accompanied by chromatin loop formation between these regions, but without cohesin CTCF is not sufficient to anchor chromatin loops. The latter is largely consistent with the published results where cohesin depletion was followed by a loss of chromatin loops (41–44).

### The N Terminus of CTCF Is Not Sufficient for Stable Cohesin Occupancy on DNA.

**Fig. 5.**
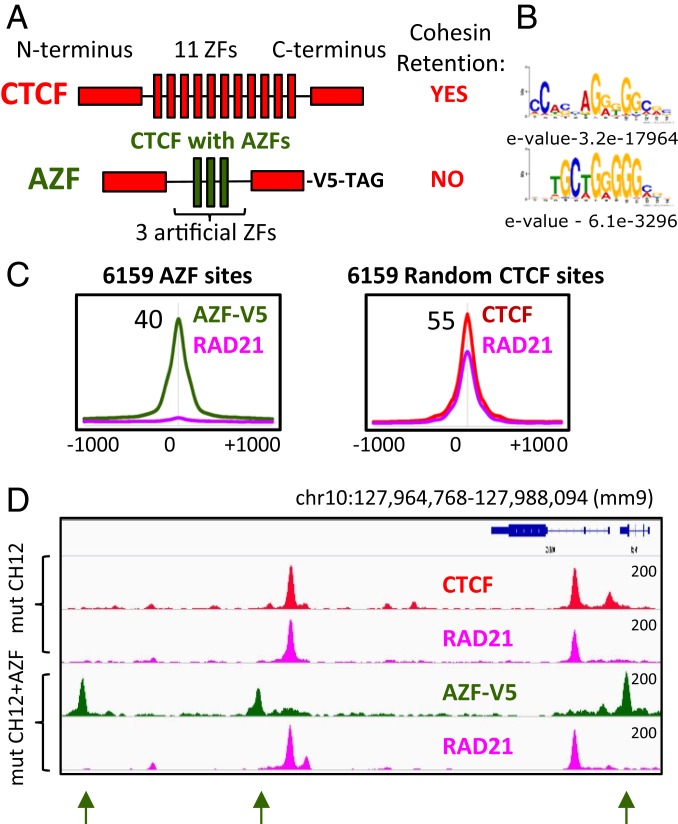
The N terminus of CTCF is not sufficient to redirect cohesin to non-CTCF binding sites. (*A*) A schematic representations of WT CTCF and a chimeric construct with three AZF flanked by the N and C termini of CTCF and tagged with V5 at the C terminus. (*B*) De novo DNA-binding motifs identified with the indicated E*-*value for the top CTCF and AZF binding sites. (*C*, *Left*) Average profile of AZF-V5 (green) and RAD21(pink) occupancy at the 6,159 sites bound by AZF in mut CH12 cells. (*Right*) Average profile of CTCF (red) and RAD21 (pink) occupancy at the 6,159 sites randomly selected from the CTCF binding sites in mut CH12 cells. (*D*) Genome browser view of CTCF (red), RAD21 (pink), and AZF-V5 (green) occupancy in mut CH12 cells. The AZF binding sites are indicated by green arrows. The absence of RAD21 occupancy at AZF binding sites is indicated by green arrows.

Would the N terminus of CTCF coupled with different DNA binding domain be sufficient for cohesin retention? To address this question, we constructed a V5 epitope-tagged chimeric construct consisting of the N and C termini of CTCF fused to three artificial ZFs (AZF) (Fig. 5*A*). For generation of this chimeric construct we used the AZF domain, which is known to bind the promoter of the vascular endothelial growth factor-A (VEGF-A) gene (45) and recognizes sequences distinct from the CTCF motif (Fig. 5*B*). The chimeric construct was ectopically expressed in mut CH12 cells, resulting in 28,329 AZF sites genome-wide, mapped by V5-Tag ChIP-seq. The AZF binding sites that were either overlapping or at least 3 kb proximal to CTCF binding sites were excluded from further analysis, leaving 6,159 CTCF-free AZF peaks (Fig. 5*C* and *SI Appendix*, Fig. S11*A*). De novo motif analysis of these AZF peaks revealed a consensus sequence highly similar to the published motif and distinct from that of CTCF (Fig. 5*B*). Next, we mapped RAD21 occupancy in these mut CH12 cells stably expressing the chimeric protein and compared its profile with the RAD21 binding pattern in the parental mut CH12 cells. In contrast to CTCF peaks, robust AZF peaks were not associated with a high cohesin occupancy, which was established by an average plot analysis (Fig. 5*C*), by heatmap of ChIP-seq data (*SI Appendix*, Fig. S11*A*), and by visualization of several individual peaks (Fig. 5*D*). Strikingly, we observed a slight enrichment above background for RAD21 occupancy alongside AZF-V5 binding (*SI Appendix*, Fig. S11*A*). These data suggest that the AZF-V5 protein may slow down sliding of cohesin along the chromatin fiber as a steric obstacle, but that it is not sufficient to stably retain in contrast to CTCF binding sites. Interestingly, the same slight enrichment of cohesin occupancy could be observed at binding sites for the transcription factors Ets1, Hcfc1, and CoRest, which have never been shown to be involved in cohesin retention (*SI Appendix*, Fig. S11 *B*–*D*), suggesting that other proteins may slow down the cohesin movement along the chromatin fiber, but do not anchor it as CTCF does. These data (*SI Appendix*, Fig. S11) may also explain the inconsistent number of cohesin non-CTCF binding sites described in the current literature (Fig. 1*A*), as some studies may use more relaxed parameters for ChIP-seq peak calling, therefore counting a slight enrichment of cohesin as a bona fide binding site. Based on the data described above, we conclude that the N terminus of CTCF is unlikely to act as an autonomously functioning domain in CTCF responsible for cohesin positioning through a direct protein–protein interaction (PPI) between CTCF and cohesin, as it is insufficient outside of CTCF target sites in this regard.

### BORIS, the CTCF Paralog, Is Not Able to Anchor Cohesin onto Chromatin.

**Fig. 6.**
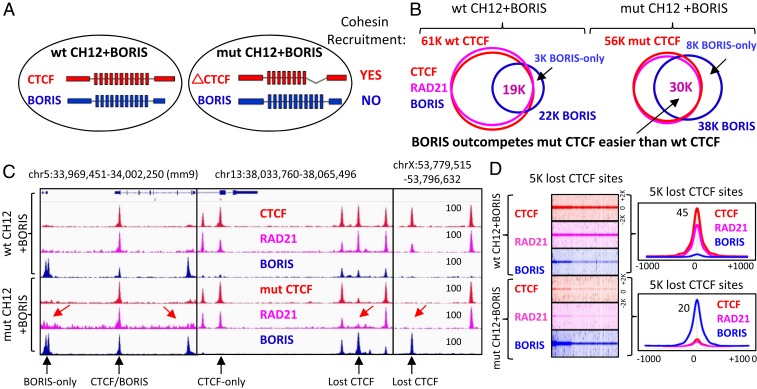
BORIS is not able to recruit cohesin onto chromatin. (*A*) Schematic representation of CTCF and BORIS expression in WT and mut CH12 cells. CTCF and BORIS proteins are highlighted by red and blue, respectively. On the right, the summary of cohesin recruitment by paralogous proteins. (*B*) Venn diagrams illustrating the overlap of CTCF (red), RAD21 (pink), and BORIS (blue) ChIP-seq peaks mapped in WT and mut CH12 cells stably expressing ectopic BORIS. (*C*) Genome browser view of CTCF (red), RAD21 (pink), and BORIS (blue) binding in WT and mut CH12 cells stably expressing ectopic BORIS. Selective CTCF and BORIS occupancies are indicated by black arrows. The absence of cohesin at BORIS-only and the lost CTCF binding sites are indicated by red arrows. (*D*, *Left*) Heatmaps of CTCF, RAD21, and BORIS binding at the 5K lost CTCF binding sites in WT and mut CH12 cells. (*Right*) Average profiles for the heatmap data.

To narrow down the regions of CTCF necessary and sufficient for retention of cohesin on chromatin, we took advantage of the CTCF germ cell-specific paralog BORIS, which contains an 11ZF DNA binding domain (DBD) that is virtually identical to that of CTCF but distinct N and C termini (Fig. 6*A* and *SI Appendix*, Fig. S12*A*) (46). BORIS selectively binds to a small subset of CTCF binding regions as a CTCF/BORIS heterodimer or as a BORIS homodimer (BORIS-only sites) (47). These binding regions frequently consist of clustered CTCF target sites (2xCTSes). Our previous work established that regions bound by BORIS alone in cancer cell lines were not occupied by cohesin subunits RAD21 and SMC3, indirectly suggesting that BORIS is not able to retain cohesin (47). To directly address the possibility of cohesin retention by BORIS we stably expressed BORIS in WT CH12 cells (*SI Appendix*, Fig. S12*B*), where BORIS is not expressed, and mapped CTCF, BORIS, and RAD21 occupancy by ChIP-seq. Similar to our previous data (47), we observed a selective pattern of BORIS occupancy: BORIS binds to a minority of CTCF binding regions (19,145, 30%, CTCF&BORIS sites), while the majority of CTCF sites (42,315, 70%, CTCF-only sites) was not stably occupied by BORIS (Fig. 6 *B* and *C* and *SI Appendix*, Fig. S12 *C* and *D*). More importantly, almost all CTCF sites in wt CH12 cells, whether occupied by BORIS or not, were bound by RAD21 as well, while BORIS-only sites showed a complete depletion of cohesin occupancy (Fig. 6 *B* and *C* and *SI Appendix*, Fig. S12*E*). Thus, we confirmed on a genome-wide level that ectopically expressed BORIS is not able to retain cohesin at BORIS-only sites, similar to endogenously expressed BORIS in cancer cell lines (47).

Having concluded that BORIS-only target sites are depleted of cohesin, it remained to be determined whether BORIS-only sites repel cohesin or if BORIS itself is not able to retain cohesin. To address these issues, we stably expressed BORIS in mut CH12 cells with the idea that, in the absence of CTCF, BORIS will bind the 5K lost CTCF sites. In a dramatic contrast to the WT CH12 cells, we mapped almost twice as many BORIS binding sites in mut CH12 cells (22,765 [WT] versus 38,744 [mut]) (Fig. 6*B*). One of the likely reasons for this is that mutant CTCF with deleted ZFs 9 to 11 is not able to compete with BORIS as effectively as WT CTCF in WT CH12 cells. As a consequence of this, BORIS effectively competes with mutant CTCF at the majority of CTCF sites, increasing the number of robustly occupied BORIS binding sites in mut CH12 cells (Fig. 6 *B*–*D* and *SI Appendix*, Fig. S13). Moreover, in the absence of CTCF occupancy, BORIS effectively binds thousands of lost CTCF sites, which explains why the number of BORIS-only binding sites increased from 3,336 in WT CH12 cells to 8,435 in mut CH12 cells (Fig. 6*B* and *SI Appendix*, Fig. S13). These data suggest that BORIS is able to compete with CTCF at the majority of genomic regions and in the presence of mutant CTCF has a competitive binding advantage (Fig. 6 *B*–*D*). Nevertheless, the stronger BORIS occupancy does not result in cohesin retention at the lost CTCF sites (Fig. 6 *C* and *D*), confirming our preliminary conclusion that BORIS is unable to retain cohesin not only at BORIS-only sites, but at CTCF sites as well.

### The Specific Interplay between CTCF ZFs and Its Target Sites Is Also Essential for Cohesin Retention.

**Fig. 7.**
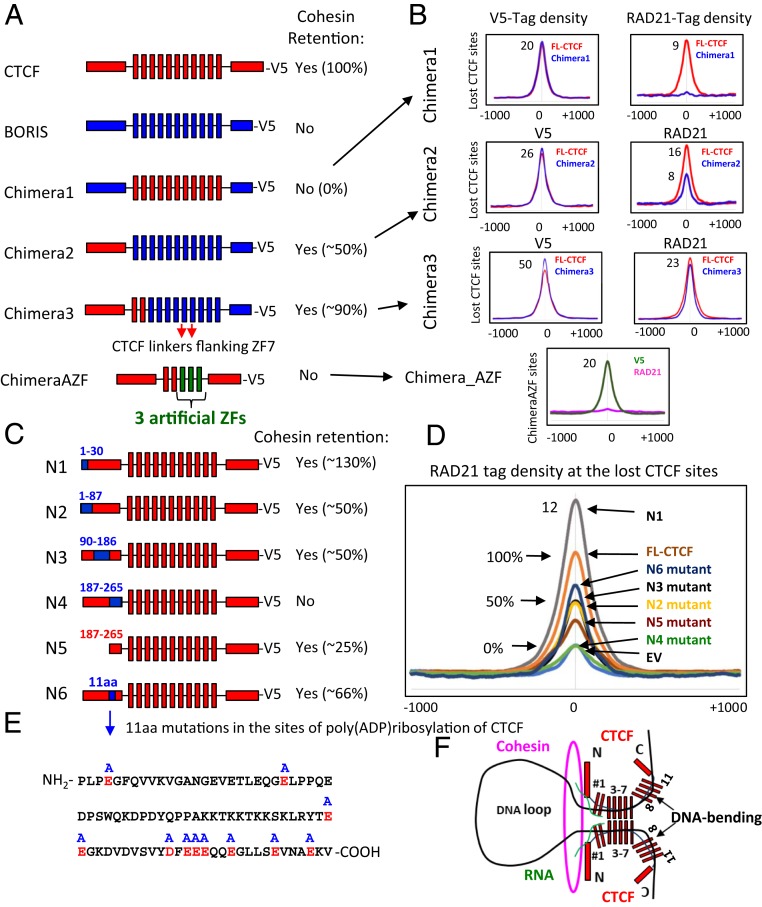
The N terminus and 11ZF domain of CTCF and its target sequence are required for cohesin retention on DNA. (*A*) Schematic representations of CTCF, BORIS, and chimeric proteins expressed in mut CH12 cells. On the right, the summary of cohesin occupancy mediated by expressed proteins at the lost CTCF sites. (*B*) Average profiles of V5 and RAD21 occupancy at the CTCF lost sites in mut CH12 cells stably expressing the proteins shown in *A*. The lost CTCF sites were selected for each chimera to be bound equally by both FL-CTCF and chimeric protein. The connections between *A* and *B* are shown by black arrows. (*C*) Schematic representation of the CTCF N-terminal mutants where CTCF (red) sequences (amino acid positions are indicated by blue numbers) were replaced with the corresponding sequence of BORIS (blue). On the right, the summary of cohesin occupancy mediated by mutant proteins at the 5K lost CTCF sites. (*D*) Average profile of RAD21 occupancy at the lost CTCF binding sites in mut CH12 cells stably expressing the mutant proteins from *C* compared to FL-CTCF. (*E*) The sequence of N6 mutant shows the 11 sites for poly(ADP ribosyl)ation of CTCF (highlighted by red color), which were mutated to Alanine (blue A at the top of the sequence) in mutant protein from *C* (shown by blue arrow). (*F*) The proposed model: CTCF retains cohesin at the anchors of chromatin loop by the 3D structure formed between the N terminus of CTCF and the first 2 ZFs of CTCF at CTCF target site bound by the central CTCF ZFs (3–7). The first CTCF ZF (#1) is more involved in RNA binding (highlighted by green color) than in DNA binding, which may contribute to the formation of 3D cohesin blocking structure. CTCF is known to bend DNA between the two groups of 11 ZFs (1–7 and 8–11), which also may contribute to blocking of cohesin extrusion.

So far, we have shown that BORIS, in contrast to CTCF, is not able to retain cohesin at either BORIS-only or the lost CTCF sites (Fig. 6). To address the question of what region of CTCF is necessary and sufficient for cohesin positioning at CTSes, we attempted to convert BORIS into CTCF with respect to cohesin retention. First, we replaced BORIS ZFs with CTCF ZFs (Chimera1) to test if the CTCF ZFs in combination with BORIS termini could retain cohesin (Fig. 7*A*). To enable determination of a genome-binding profile of the chimeric protein, a V5-tag was introduced at the C terminus of protein. The overall genomic distribution of Chimera1 in mut CH12 cells was well-correlated with those of CTCF (Pearson correlation = 0.73) and BORIS (Pearson correlation = 0.78); however, analysis of specific subsets of bound loci revealed notable differences (*SI Appendix*, Fig. S14). In particular, Chimera1 was not able to occupy most BORIS-only sites and, at the same time, was able to bind a subset of CTCF-only sites that were depleted of BORIS occupancy in mut CH12+BORIS cells, suggesting a moderate degree of divergence in the sequence specificity of CTCF and BORIS ZFs (*SI Appendix*, Fig. S14 *B*–*D*). At the same time, a higher Pearson correlation between Chimera1 and BORIS versus Chimera1 and CTCF (*SI Appendix*, Fig. S14*A*) suggests that, although the N and C termini of BORIS are not involved in direct DNA binding, they contribute to protein occupancy, perhaps by interaction with specific protein cofactors. We then compared ChIP-seq data for cohesin (RAD21) occupancy in mut CH12 cells stably expressing either Chimera1, BORIS, or FL-CTCF (*SI Appendix*, Fig. S14*E*). While Chimera1 binds the 5K lost CTCF sites in mut CH12 cells, similar to FL-CTCF, it was unable to retain cohesin (Fig. 7*B* and *SI Appendix*, Fig. S14*E*). Thus, we demonstrated by several approaches that without the N terminus of CTCF, the CTCF ZFs bound to CTCF target sites are not sufficient for cohesin retention.

In a further attempt to convert BORIS into CTCF-like with respect to cohesin retention, we generated another chimeric protein by replacing the N terminus of BORIS with the N terminus of CTCF (Chimera2), tagged this chimera with V5, and expressed it in mut CH12 cells (Fig. 7*A*). The binding pattern of Chimera2 was more similar to that of BORIS (Pearson correlation = 0.88) than of CTCF (Pearson correlation = 0.76) in CH12 mutant cells (*SI Appendix*, Fig. S14*A*). Similar to BORIS, Chimera2 was able to bind a majority of BORIS-only sites and avoid some CTCF-only sites, further suggesting small differences in CTCF and BORIS ZF sequence-recognition properties (*SI Appendix*, Fig. S15). The occupancy by cohesin (RAD21) in mut CH12 cells stably expressing either Chimera2 or FL-CTCF proteins was then analyzed for the 5K lost CTCF sites. We observed an overall gain of RAD21 occupancy following the gain of Chimera2 occupancy, albeit to a lesser extent than with FL-CTCF stably expressed in mut CH12 cells (Fig. 7*B* and *SI Appendix*, Fig. S15*D*). *K*-means–ranked clustering of ChIP-seq data along the 5K lost CTCF sites and examination of peaks at a sample of these sites showed that only some of them were enriched with cohesin reflecting Chimera2 occupancy, and almost all of them were occupied by cohesin following FL-CTCF occupancy (*SI Appendix*, Fig. S15*D*).

As we showed here that CTCF and BORIS ZFs have some differences in sequence preference (*SI Appendix*, Figs. S14 and S15), we then focused on the lost CTCF sites that were occupied by both proteins at relatively similar levels (Fig. 7*B* and *SI Appendix*, Fig. S15*D*) in order to directly compare the retention of cohesin by Chimera2 and FL-CTCF. The comparison of RAD21 occupancy at the sites equally enriched for both CTCF and BORIS ZFs showed that Chimera2 is able to retain cohesin, albeit with ∼50% lower efficiency than the FL-CTCF (Fig. 7*B*).

Having established that Chimera2 is able to partially retain cohesin, it was even more striking to observe that Chimera2 was not able to retain cohesin at the BORIS-only sites and some of the lost CTCF sites (*SI Appendix*, Fig. S15 *C* and *D*). Taken together, these observations suggest that cohesin occupancy not only depends on the N terminus of CTCF but also on the sequence of target sites and whether a given site is bound by either CTCF or BORIS ZFs combined with the N terminus of CTCF. Apparently, the interaction of the CTCF 11ZF DBD and its target sequence and dimerization of CTCF through its 11ZFs are also essential for cohesin retention. We speculate that the 3D structure of either CTCF or BORIS proteins bound to chromatin will depend on how their ZFs interact with each binding site. Cohesin retention may thus depend not only on the N terminus of CTCF but also on the 3D structure of the proteins in complex with DNA, explaining why Chimera2 with BORIS ZFs binds to CTCF target sequences but, in contrast to CTCF, is not able to retain cohesin correctly. In line with this idea, the replacement of CTCF ZFs with BORIS ZFs in CTCF impaired cohesin retention by the chimeric construct (Chimera4) (*SI Appendix*, Fig. S16), suggesting that CTCF ZFs are also involved in the retention of cohesin.

Having established that the CTCF ZFs are also essential to cohesin retention, we asked what specific ZFs are engaged in this function and why BORIS ZFs do not function similarly. Based on the data presented above, where we showed that mut CTCF consisting of ZFs 1 to 8 was able to retain cohesin at the remaining CTCF sites (Fig. 2) and to form chromatin loops in mut CH12 cells (Fig. 4), we assume that ZFs 9 to 11 are not essential for CTCF retention of cohesin. With respect to sequence homology in ZFs 1 to 8, CTCF and BORIS are relatively similar (80% homology level) (*SI Appendix*, Fig. S17). However, a more detailed analysis of the CTCF and BORIS ZF amino acid sequence alignment showed that there are several amino acid substitutions in the first two ZFs and in the noncanonical linkers flanking ZF7 of CTCF (*SI Appendix*, Fig. S17). To test if these amino acid sequence differences account for the ability of CTCF but not Chimera2 to position cohesin at the target sites, we generated Chimera3 by replacing the first 2 BORIS ZFs and the linkers flanking ZF7 in Chimera2 with the corresponding sequences of CTCF (Fig. 7*A*). Subsequent analysis of RAD21 occupancy in mut CH12 cells stably expressing the chimeric protein showed that, overall, Chimera3 was much more effective in retention of cohesin than Chimera2, but still less effective compared to FL-CTCF (90% for Chimera3 vs. FL-CTCF) (Fig. 7*B* and *SI Appendix*, Fig. S18). In addition, cohesin occupancy followed Chimera3 occupancy at some but not all of the lost CTCF target sites in mut CH12 cells (*SI Appendix*, Fig. S18*B*).

Apparently, the closer we modify BORIS ZFs to CTCF ZFs, the higher cohesin enrichment at CTCF sites by chimeric proteins becomes (*SI Appendix*, Fig. S18*D*). In line with this, the deletion of the first two ZFs of CTCF severely affected cohesin retention at the 5K lost CTCF sites compared to FL-CTCF expressed in mut CH12 cells, suggesting that these two ZFs are also essential for cohesin positioning at CTCF target sites (*SI Appendix*, Fig. S19). To see if a combination of the N terminus of CTCF with the first two CTCF ZFs would be sufficient to retain cohesin on DNA, we generated ChimeraAZF by replacing the ZFs 3 to 11 in CTCF with the three artificial ZFs mentioned in Fig. 5. Subsequent analysis of RAD21 occupancy in mut CH12 cells showed that ChimeraAZF protein is unable to retain cohesin at the AZF-specific binding sites (Fig. 7 *A* and *B*). Thus, we suggest that not only the N terminus of CTCF and the first two CTCF ZFs are involved in cohesin retention, but also the 3D conformation of the CTCF–DNA complex mediated by a specific interplay between CTCF ZFs and its target DNA.

### A 79-aa Region in the N Terminus of CTCF Is Necessary for Cohesin Occupancy at CTCF Binding Sites.

The N terminus of CTCF has been reported to interact with multiple protein partners and contains a number of sites for posttranslational modifications, such as acetylation, SUMOylation, phosphorylation, and poly(ADP ribosyl)ation (48–51). As some of these posttranslational modifications have been reported to be essential for CTCF insulator function (49, 50), we explored the role of the CTCF N terminus in cohesin retention by generating a set of four different mutant forms. To this end, we replaced each CTCF amino acid sequence with the corresponding BORIS amino acid sequence (Fig. 7*C* and *SI Appendix*, Fig. S20): N1 mutant (amino acids 1 to 30 replaced), N2 mutant (1 to 87 aa), N3 mutant (90 to 186 aa), and N4 mutant (187 to 265 aa). These chimeric constructs were also tagged with V5 at the C terminus and stably expressed in mut CH12 cells (*SI Appendix*, Fig. S21*A*). Next, we analyzed chimeric protein binding profiles (V5-tag) and cohesin (RAD21) occupancy in mut CH12 cells (Fig. 7*D* and *SI Appendix*, Figs. S21*B* and S22*A*). As seen in Fig. 7*D*, all of the N terminus mutants had some impact on cohesin retention at the lost CTCF binding sites. The replacement of the first 30 aa in CTCF resulted in higher cohesin occupancy (130%) at the 5K lost CTCF sites followed by N1 mutant binding compared to FL-CTCF. The replacement of 1 to 87 aa in the N terminus, the sequence containing sites for SUMOylation, reduced cohesin retention twofold compared to FL-CTCF. The same outcome was observed for the N3 mutant (Fig. 7*D*). Most importantly, only the N4 mutant (replacement of 186 to 265 aa) completely abolished the ability of CTCF to anchor cohesin, as defined by the absence of cohesin enrichment following N4 mutant occupancy at the 5K lost CTCF sites (Fig. 7*D* and *SI Appendix*, Fig. S22*A*).

To elucidate whether the amino acid sequence replaced in the N4 mutant is sufficient to retain cohesin, we generated a truncated form of CTCF with only the 79 most C-terminal amino acids of the N terminus remaining (N5 mutant) and stably expressed this construct in mut CH12 cells. ChIP-seq analysis of the N5 mutant and RAD21 occupancy at the 5K lost CTCF binding sites confirmed that this sequence is indeed necessary but not sufficient for full cohesin retention, as it restored cohesin occupancy to ∼25% of that imposed by FL-CTCF expression (Fig. 7*D*). Incidentally, the N4 sequence was shown to include multiple putative sites for poly(ADP ribosyl)ation, the posttranslational modification that is considered indispensable for CTCF functions (49). To test whether the poly(ADP ribosyl)ation of CTCF is a key for cohesin retention, we generated a new mutant (N6) by replacing 11 aa in the N terminus of CTCF that were previously mapped by mass spectrophotometry as the sites for PARlytion of CTCF by poly(ADP ribose) polymerase (PARP)1 (52) (Fig. 7 *C* and *E*). Indeed, the mutations of 11 aa involved in this CTCF posttranslational modification affected cohesin positioning at CTCF sites, but did not eliminate it as completely as with the N4 mutant, suggesting that poly(ADP ribosyl)ation of CTCF could be involved in chromatin loop formation but is not the only factor necessary for cohesin retention. Consistent with this hypothesis, treatment of WT CH12 cells with a PARP inhibitor (3ABA) for 3 d only moderately affected CTCF and RAD21 occupancy (*SI Appendix*, Fig. S22 *B*–*D*). Thus, we narrowed down a 79-aa sequence in the N terminus of CTCF that is essential for the cohesin positioning, although other portions of the N terminus are also necessary.

## Discussion

Understanding how 3D genome organization is established has been of great interest for decades. Although it has been widely reported that CTCF and cohesin complex are the key players in the establishment of higher-order chromatin organization in mammalian cells, the nature and mechanism of their interactions are still unclear (53). In this study, we examined the dependence of cohesin occupancy on CTCF binding and delineated protein domains of CTCF involved in cohesin retention.

A key finding of our study is that CTCF and cohesin binding sites nearly fully coincide in the human and mouse genomes, contradicting previous studies reporting highly variable proportions of nonoverlapping binding sites of the two factors (20–22). Indeed, only a small minority of cohesin binding sites was depleted of CTCF, and those were occupied by the cohesin-loading factor NIPBL (Fig. 1). These results are consistent with the known interaction of cohesin with both factors (10, 11, 28). One of the explanations of the fluctuating number of CNC sites (Fig. 1) could be a nonspecific slowing down of cohesin by multiple transcription factors bound to chromatin, and thus creating a steric obstacle on the way of cohesin sliding along the chromatin fiber (as shown in *SI Appendix*, Fig. S11). As a complement to our genome-wide comparisons of CTCF and cohesin binding, we performed a thorough analysis of CTCF antibody performance in ChIP-seq, finding that a number of antibodies fail to detect the full spectrum of CTCF sites (*SI Appendix*, Fig. S3). This observation suggests that the high prevalence of CNC sites in previous studies may be due to reduced sensitivity of antibodies in mapping of CTCF. We also show that, in contrast to previous work, cell type-specific transcription factors (e.g., ESR1 in MCF7 cells, CEBPA in HepG2 cells, and pluripotency factors in mESCs) (20–22, 26, 54) showed no preference for the strong colocalization with cohesin binding sites (*SI Appendix*, Fig. S2) but instead show the slight nonspecific enrichment of cohesin occupancy observed as well with other transcription factors and the AZF chimeric protein (*SI Appendix*, Fig. S11).

Second, and perhaps most surprisingly, we discovered that cohesin occupancy at CTCF binding sites depends on the N terminus of CTCF. We employed a mut CH12 cell line in which, due to a homozygous deletion of ZFs 9 to 11, CTCF occupancy is lost at the 5K sites, but remains the same at the 56K CTCF sites with a concomitant loss and no change of cohesin occupancy at these same loci, respectively (Fig. 2). We found that the CTCF occupancy at the 5K lost sites in mut CH12 cells could be restored by either FL-CTCF and chimeric or mutant proteins as long as the protein has 11 ZFs of CTCF, thus providing a valuable system to study CTCF and cohesin interactions in vivo. The 5K lost and the 56K remaining CTCF sites are very similar with respect to the positioning of cohesin at genomic loci in CTCF-dependent manner (Fig. 2 *C* and *E*), as well as to their involvement in 3D genome organization (Fig. 4), genomic distribution (*SI Appendix*, Fig. S5*A*), and association with epigenetic marks and transcription factors (*SI Appendix*, Fig. S5*B*). Therefore, the study of CTCF and cohesin interactions at the 5K lost CTCF sites could be extrapolated to the genome-wide level. Using this system, we found that while the forms of CTCF lacking either the N or C terminus efficiently restored CTCF occupancy at the 5K lost binding sites, only the C-terminally truncated CTCF was able to restore both cohesin occupancy and chromatin loop formation, indicating the N terminus essentiality for these related phenomena (Figs. 3 and 4). In contrast, the C terminus of CTCF was dispensable for cohesin occupancy and loop formation, challenging previous work reporting that the C terminus of CTCF directly interacts with SA2, an external subunit of cohesin ring, and thus provides a bridge between CTCF and the cohesin complex (34). A role for the N terminus of CTCF in cohesin retention is consistent with high-resolution ChIP-seq analysis showing that cohesin binding tends to be shifted toward the N terminus of CTCF (37). Besides, an alternative form of CTCF missing its N terminus was shown to be unable to retain cohesin at CTCF binding sites (55). Furthermore, we narrowed down a subdomain of CTCF necessary for full cohesin retention (i.e., a 79-aa segment of the CTCF N terminus adjacent to the first ZF). The N terminus of CTCF is, nevertheless, insufficient for full cohesin retention outside of CTCF target sites (Figs. 5 and 7).

Third, we showed that the CTCF paralog BORIS is unable to retain cohesin on DNA. BORIS possesses a highly similar DBD but distinct N and C termini compared to CTCF (46, 56). As a result, the two proteins are able to bind to essentially the same DNA sequences in vivo but interact with distinct protein partners (57). We previously found that BORIS preferentially binds to a specific subset of genomic regions consisting of clustered CTCF binding motifs (termed 2xCTSes) as putative BORIS homodimers (BORIS-only sites) or as a heterodimer with CTCF (CTCF&BORIS sites) (47, 58). In cancer cells where BORIS expression is aberrantly activated, BORIS-only sites were depleted of cohesin occupancy, while CTCF&BORIS sites were enriched with cohesin, indirectly suggesting that BORIS alone is not able to retain cohesin (47). Indeed, we found that expression of BORIS in mut CH12 cells was insufficient to restore cohesin occupancy at the 5K lost CTCF sites (Fig. 6). Thus, CTCF and BORIS evolutionary paths diverged dramatically, with CTCF possessing an ability to retain cohesin and form chromatin loops. As BORIS expression is strictly restricted to germ cells in normal development, the inability of BORIS to interact with somatic cohesin may be required during spermatogenesis to disrupt chromatin loops formed by CTCF in progenitor cells or to form a distinct chromatin architecture in collaboration with meiosis-specific cohesin subunits. We were also able to address the long-standing question of the sequence specificities of CTCF and BORIS in the genome, long presumed to be identical due to their highly conserved 11ZF DNA binding domains (59). We showed that there is a moderate degree of divergence in sequence specificity of CTCF and BORIS ZFs, as the replacement of BORIS ZFs with CTCF ZFs in BORIS resulted in a DNA binding pattern similar to CTCF (*SI Appendix*, Fig. S14). As BORIS was not able to retain cohesin, we attempted to delineate the regions of CTCF that are necessary and sufficient for cohesin retention by converting BORIS into CTCF in respect to this function. Replacing different sequences of BORIS with the corresponding sequences of CTCF, we found that a chimera consisting of the N terminus of CTCF combined with the first two CTCF ZFs followed by the rest of the BORIS sequence is necessary and sufficient for cohesin retention at CTCF binding sites.

Our results also shed light into the molecular mechanism of CTCF-mediated retention of cohesin at chromatin loop anchors, for which two hypotheses have been previously proposed. According to the first, CTCF directly recruits cohesin at its binding sites through PPIs (34). Alternatively, CTCF dimerization was proposed to prevent the translocation of cohesin ring along the chromatin fiber, not specifying with or without a specific PPI (16, 17). In the context of these two hypotheses, our data suggest that CTCF blocks cohesin translocation through the 3D structure formed by the N terminus and the first 2 ZFs of CTCF at CTCF target sites without a direct PPI between CTCF and the cohesin complex. In support of this idea, we showed that, while the N terminus of CTCF is necessary for cohesin retention, it is not sufficient outside of CTCF binding sites. Indeed, the N terminus of CTCF fused with either AZF or the first two CTCF ZFs plus AZF is not able to immobilize cohesin on chromatin, suggesting a lack of an autonomously functioning domain in CTCF responsible for cohesin positioning. Furthermore, the chimeric construct, essentially representing BORIS with the N terminus of CTCF, is able to anchor cohesin at some CTCF binding sites, but not others; the latter includes BORIS-only sites, suggesting that cohesin retention at CTCF target sites has a sequence-specific component. There are several other lines of evidence against a stable complex between CTCF and cohesin in the absence of DNA bridging. First, we were unable to coimmunoprecipitate CTCF with any cohesin subunits (*SI Appendix*, Fig. S23) in the presence of ethidium bromide, which ensures detection of only direct PPIs without bridging by nucleic acid (60). Second, an EMSA with size-fractionated nuclear extracts failed to detect a stable complex of CTCF with cohesin while readily detecting a CTCF–BORIS interaction (*SI Appendix*, Fig. S24). Third, a recently published liquid chromatin Hi-C study provides direct evidence for a lack of stable CTCF-cohesin PPI, as cohesin easily slides off from DNA ends in unfixed chromatin fragments (61). Finally, all N-terminal mutants of CTCF affect cohesin retention on chromatin to some degree, suggesting that there is no particular amino acid sequence in the N terminus of CTCF responsible for a physical interaction with cohesin (Fig. 7*D*).

How does CTCF stall cohesin movement on chromatin? A simple consideration that cohesin existed before CTCF has emerged in evolution suggests that there must be a more general mechanism to position the cohesin complex, particularly by a structural hindrance impeding its translocation (62, 63). Therefore, it is highly plausible that CTCF participates in a similar roadblock mechanism. Taking into account the fact that the two essential cohesin retention regions, the 79 aa of the N terminus of CTCF and the first two ZFs, are directly adjacent to each other, we speculate that these two regions may form a 3D structure that protrudes from chromatin and blocks cohesin sliding along chromatin during loop extrusion (Fig. 7*F*). The enrichment in stretches of negatively charged aspartates and glutamates in the 79-aa sequence may facilitate the formation of such a structure via the repulsion of the N terminus of CTCF from negatively charged DNA. Moreover, the first two CTCF ZFs are not significantly involved in DNA binding (32), but instead have been shown to be involved in RNA binding (64, 65). Interestingly, CTCF interactions with RNA have been shown to be essential for 3D chromatin organization (36, 66). Thus, RNA-binding by the first CTCF ZF may also hypothetically contribute to the formation of such a cohesin-blocking structure by creating additional steric constraints for cohesin ring sliding (Fig. 7*F*). As the N terminus of CTCF combined with the first 2 ZFs attached to AZF is not able to retain cohesin outside of CTCF binding sites (ChimeraAZF) (Fig. 7 *A* and *B*), it is likely that the central CTCF ZFs bound to CTCF target site also contribute to cohesin retention, perhaps through bending of DNA by CTCF ZFs (67, 68). In addition, we cannot exclude that 3D conformation of CTCF may also block translocation of cohesin by inhibiting its ATPase activity. Taken together, our results substantiate and provide mechanistic details on the close cooperation between CTCF and cohesin in shaping the 3D architecture of eukaryotic genomes and provide detailed insight into the mechanism of cohesin retention by CTCF.

## Materials and Methods

Comprehensive experimental details are provided in *SI Appendix*, *Materials and Methods*, including detailed description of cell culture, sources of antibodies, plasmids, reagents, and detailed methodological descriptions. For data availability, next-generation data have been deposited in the Gene Expression Omnibus (GEO) repository with accession numbers GSE136122 and GSE137216.

## Supplementary Material

Supplementary File
