# Supplementary material for: CTCF mediates chromatin looping via N-terminal domain-dependent cohesin retention
Source: Proc Natl Acad Sci U S A. 2020 Jan 14;117(4):2020–31. doi: 10.1073/pnas.1911708117 (PMC6995019; doi:10.1073/pnas.1911708117)
Supplement: Supplementary File [file pnas.1911708117.sapp.pdf]

PNAS
www.pnas.org

Supplementary Information for

CTCF mediates chromatin looping via N-terminal domain-dependent cohesin retention.

Elena M. Pugacheva, Naoki Kubo, Dmitri Loukinov, Md Tajmul, Sungyun Kang, Alexander L. Kovalchuk, Alexander V. Strunnikov, Gabriel E. Zentner, Bing Ren, Victor V. Lobanenkov

Elena Pugacheva, Victor Lobanenkov
epugacheva@niaid.nih.gov; vlobanenkov@niaid.nih.gov;

This PDF file includes:

Figures S1 to S25
Supplementary text
SI Materials and Methods

www.pnas.org/cgi/doi/10.1073/pnas.1911708117

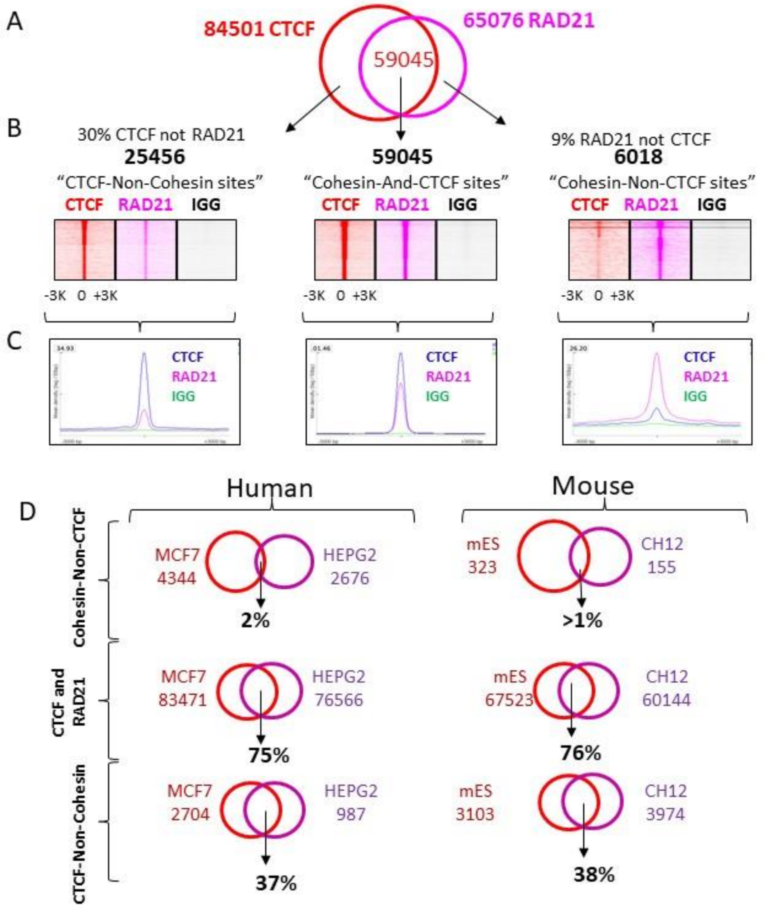

Fig. S1. Overlap of CTCF and cohesin occupancy in multiple cell lines. (A) Venn diagram representing overlap of CTCF and RAD21 ChIP-seq binding regions mapped in MCF7 cells. **(B)** Heatmaps of CTCF (red), RAD21 (pink) and IgG (black) occupancy at genomic regions bound either by CTCF or RAD21 or both in MCF7 cells demonstrate that both CTCF and RAD21 peaks not overlapping with each other show some enrichment of RAD21 and CTCF occupancy, respectively. The heatmaps correspond to the overlapping CTCF and RAD21 binding sites in panel (A), with the connection between two panels shown by black arrows. **(C)** Average profiles of CTCF (blue), RAD21 (pink), and IgG (green) occupancy at the binding sites determined in panel A confirm the enrichment of RAD21 and CTCF occupancy at CTCF and RAD21 peaks not overlapping with each other, respectively. The connection between two panels (B and C) is shown by black brackets. **(D)** Based on the enrichment of CTCF and RAD21 occupancies mapped in human (MCF7 and HEPG2) and mouse (mES and CH12) cells, the three classes of CTCF and RAD21 sites were identified (labelled on the left). Venn diagrams illustrate the overlap of these three classes between the two human and mouse cell types. The percentages show how many of the sites from the cell type with a smaller number of sites overlap with the cell type with the larger number of sites. CTCF and RAD21 sites were the most reproducible, followed by CTCF sites depleted of RAD21 enrichment, while CNC sites were more cell type-specific.

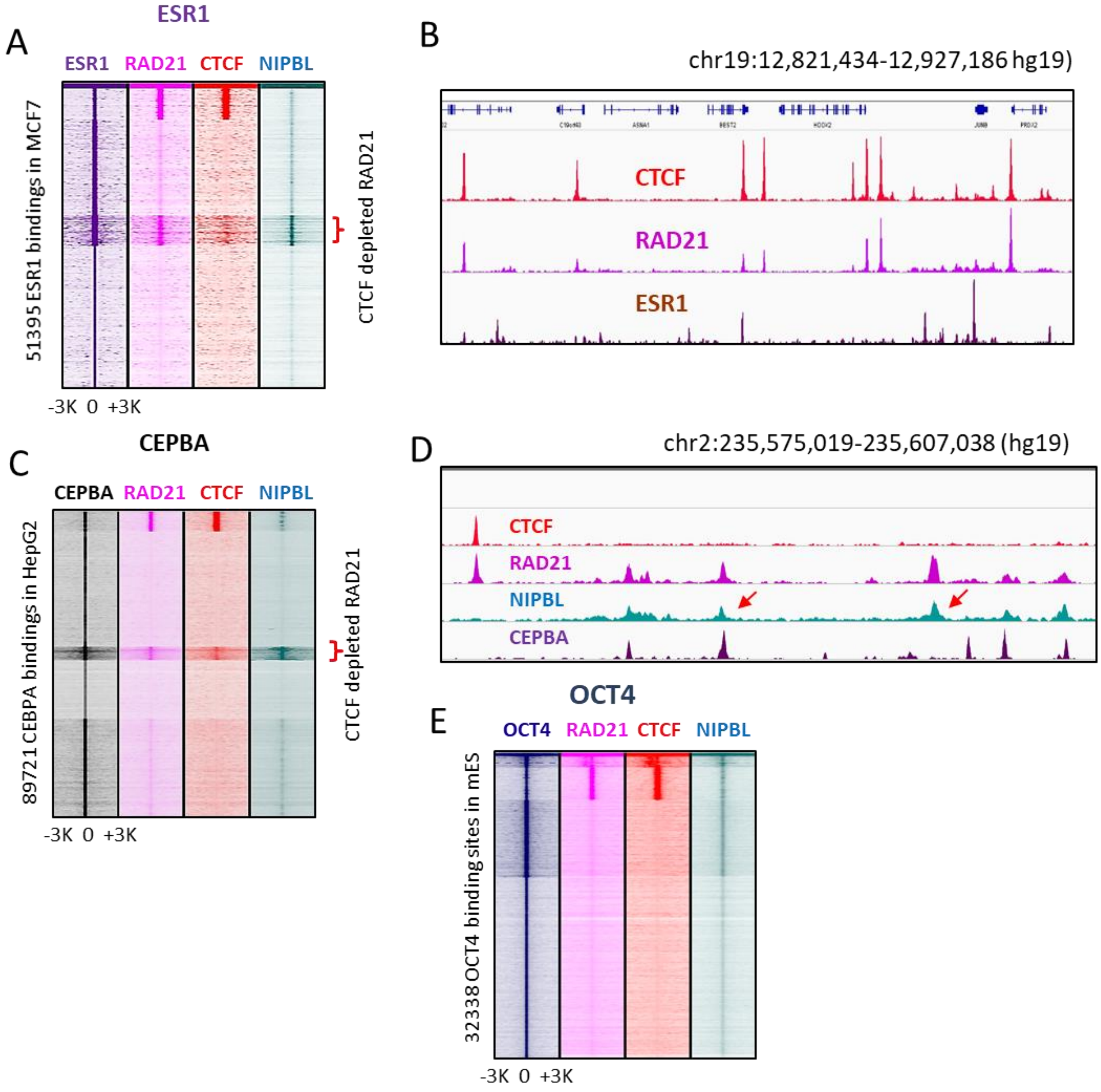

Fig. S2. The cohesin loading factor NIPBL is sufficient to explain CTCF-independent cohesin occupancy in different mouse and human cell lines, while the tissue-specific transcription factors ESR1 (MCF7), CEBPA (HepG2), OCT4 (mESC) do not generally overlap with cohesin (RAD21). (A) Heatmaps of ESR1 (purple), CTCF (red), RAD21 (pink) and NIPBL (green) occupancy at 51,395 ESR1 binding sites mapped in MCF7 cells. **(B)** Genome browser view of CTCF, RAD21 and ESR1 ChIP-seq data in MCF7 cells confirms that ESR1 binding sites generally do not coincide with cohesin occupancy. **(C)** Heatmaps of CEPBA (black), CTCF (red), RAD21 (pink), and NIPBL (green) occupancy at 89,721 CEBPA binding sites mapped in HepG2 cells demonstrate that CEPBA binding sites are generally do not coincide with CTCF-depleted cohesin binding sites. **(D)** Genome browser view of CTCF, RAD21, NIPBL, and CEBPA occupancy in HepG2 cells shows that RAD21 sites depleted of CTCF correspond better to NIPBL binding sites than CEPBA sites, highlighted by red arrows. **(E)** Heatmaps of OCT4 (blue), CTCF (red), RAD21 (pink) and NIPBL (green) occupancy at 32,338 OCT4 binding sites mapped in mESCs.

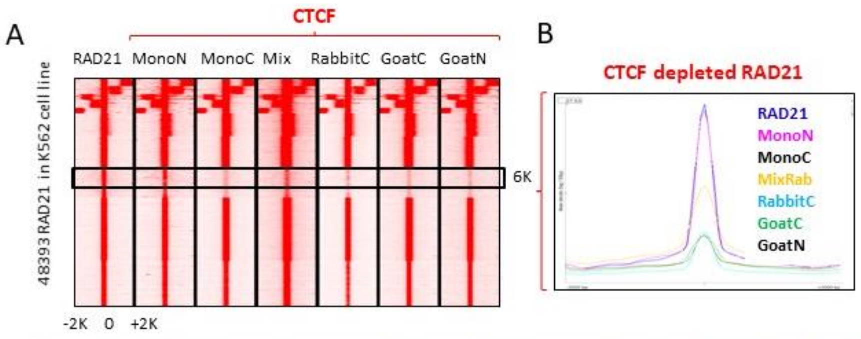

C

CTCF Abs (Name)	CTCF Abs Description	Unique reads, Million	Number of peaks
CTCF_SC-15914 (GoatC)	Goat polyclonal, C-terminus of CTCF	44.1	75326
CTCF_Millipore 07-729 (RabbitC)	Rabbit polyclonal, C-terminus of CTCF	19.0	58302
CTCF_SC-5916 (GoatN)	Goat polyclonal, N-terminus of CTCF	24.2	63534
CTCF_MonoN (MonoN)	Custom made mix of 9 N-terminus mouse monoclonal	15.6	75323
CTCF_ SC-271514 (MonoC)	Mouse monoclonal, C-terminus of CTCF	15.7	68587
CTCF_PolyC (MixRab)	Mix of 7 rabbit polyclonal Abs(*-Info in Fig.Legend)	25.1	96154

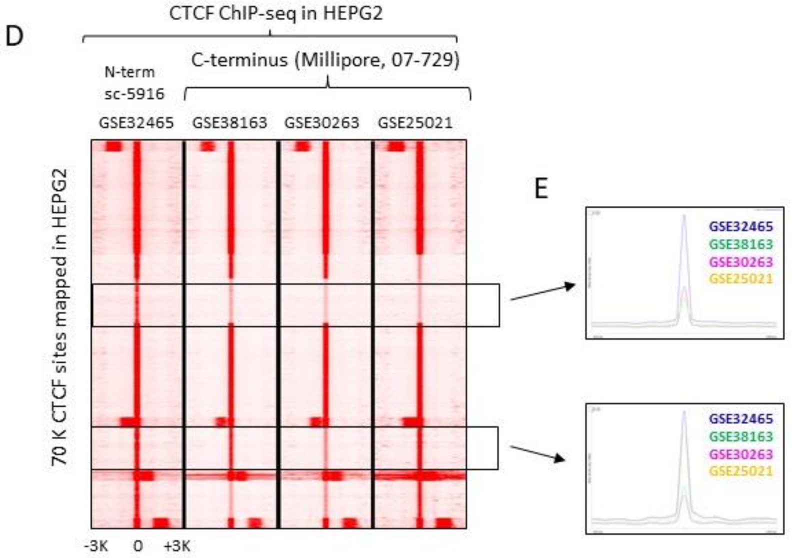

Fig. S3. Under-mapping of CTCF binding sites may explain the wide range of reported CNC site proportions. (A) Heatmaps showing RAD21 and CTCF occupancy at 48,393 RAD21 binding sites mapped by ENCODE in K562 cells. CTCF occupancy was mapped with 6 different sets of CTCF Abs, labelled at the top of heatmap and described in the table shown in **(C)**. A common set of ~6K sites not detected by several antibodies is bracketed. **(B)** Average profile of CTCF occupancy mapped with different Abs at the 6K RAD21 binding sites with variable CTCF occupancy. **(C)** Information regarding CTCF Abs used in ChIP-seq to map CTCF occupancy in K562 cells. * Mix of rabbit polyclonal (Novus Biologicals: NB100-56494, NB100-947, NB500-194, Abcam: ab70303; Bethyl Laboratories: A300-543A, A300-544A, A300-542A. **(D)** Heatmaps showing CTCF occupancy at 70K CTCF binding sites mapped by ChIP-seq in HepG2 cells. Two clusters with variable CTCF occupancies mapped by different studies are marked by black frames. **(E).** Averages plot of CTCF occupancy at the two clusters with variable CTCF occupancies mapped in panel **(D)**.

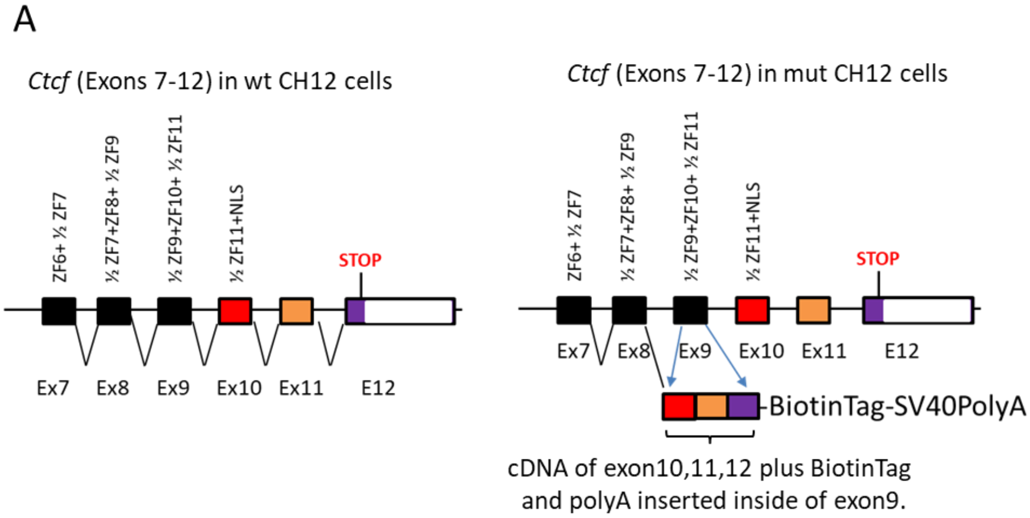

B

Alignment of wt CTCF (1-736aa) and mut CTCF (1-675aa) proteins starting from aa 300

```
                 ZF2                    ZF3                  ZF4
wtCTCF    RAFRTVTLLRNHLNTHTGTRPHKCPDCDMAFVTSGELVRHRRYKHTHEKPFKCSMCDYAS 360
mutCTCF   RAFRTVTLLRNHLNTHTGTRPHKCPDCDMAFVTSGELVRHRRYKHTHEKPFKCSMCDYAS 360
          ************************************************************
                                ZF5                  ZF6
wtCTCF    VEVSKLKRHIRSHTGERPFQCSLCSYASRDTYKLKRHMRTHSGEKPYECYICHARFTQSG 420
mutCTCF   VEVSKLKRHIRSHTGERPFQCSLCSYASRDTYKLKRHMRTHSGEKPYECYICHARFTQSG 420
          ************************************************************
                                ZF7                  ZF8
wtCTCF    TMKMHILQKHTENVAKFHCPHCDTVIARKSDLGVHLRKQHSYIEQGKKCRYCDAVFHERY 480
mutCTCF   TMKMHILQKHTENVAKFHCPHCDTVIARKSDLGVHLRKQHSYIEQGKKCRYCDAVFHERY 480
          ************************************************************
                               ZF9                   ZF10
wtCTCF    ALIQHQKSHKNEKRFKCDQCDYACRQERHMIMHKRTHTGEKPYACSHCDKTFRQKQLLDM 540
mutCTCF   ALIQHQKSHKNEKRFKCDQCDYACRQ---------------------------------- 506
          **************************
                               ZF11            C-terminus
wtCTCF    HFKRYHDPNFVPAAFVCSKCGKTFTRRNTMARHADNCAGPDGVEGENGGETKKSKRGRKR 600
mutCTCF   ---------------------------NTMARHADNCAGPDGVEGENGGETKKSKRGRKR 539
                                     *********************************
                               C-terminus
wtCTCF    KMRSKKEDSSDSEENAEPDLDDNEEEEEPAVEIEPEPEPQPQPPPPPQPVAPAPPPAKKR 660
mutCTCF   KMRSKKEDSSDSEENAEPDLDDNEEEEEPAVEIEPEPEPQPQPPPPPQPVAPAPPPAKKR 599
          ************************************************************
                               C-terminus
wtCTCF    RGRPPGRTNQPKQNQPTAIIQVEDQNTGAIENIIVEVKKEPDAEPAEGEEEEAQAATTDA 720
mutCTCF   RGRPPGRTNQPKQNQPTAIIQVEDQNTGAIENIIVEVKKEPDAEPAEGEEEEAQAATTDA 659
          ************************************************************

wtCTCF    PNGDLTPEMILSMMDR 736
mutCTCF   PNGDLTPEMILSMMDR 675
          ****************
```

Fig. S4. Genomic and sequence differences in *Ctcf* locus in wt and mut CH12 cells. (A) cDNA that encodes exons 10, 11, 12 of *Ctcf* followed by BiotinTag, Stop signal, and SV40-polyA sequences was homozygously inserted into exon 9 by CRISPR technology, thus creating mut CTCF with only 8 ZFs compared to wt CTCF. (B) Alignment of wt and mut CTCF proteins starting from aa #300. CTCF ZFs are labelled by red color and lines.

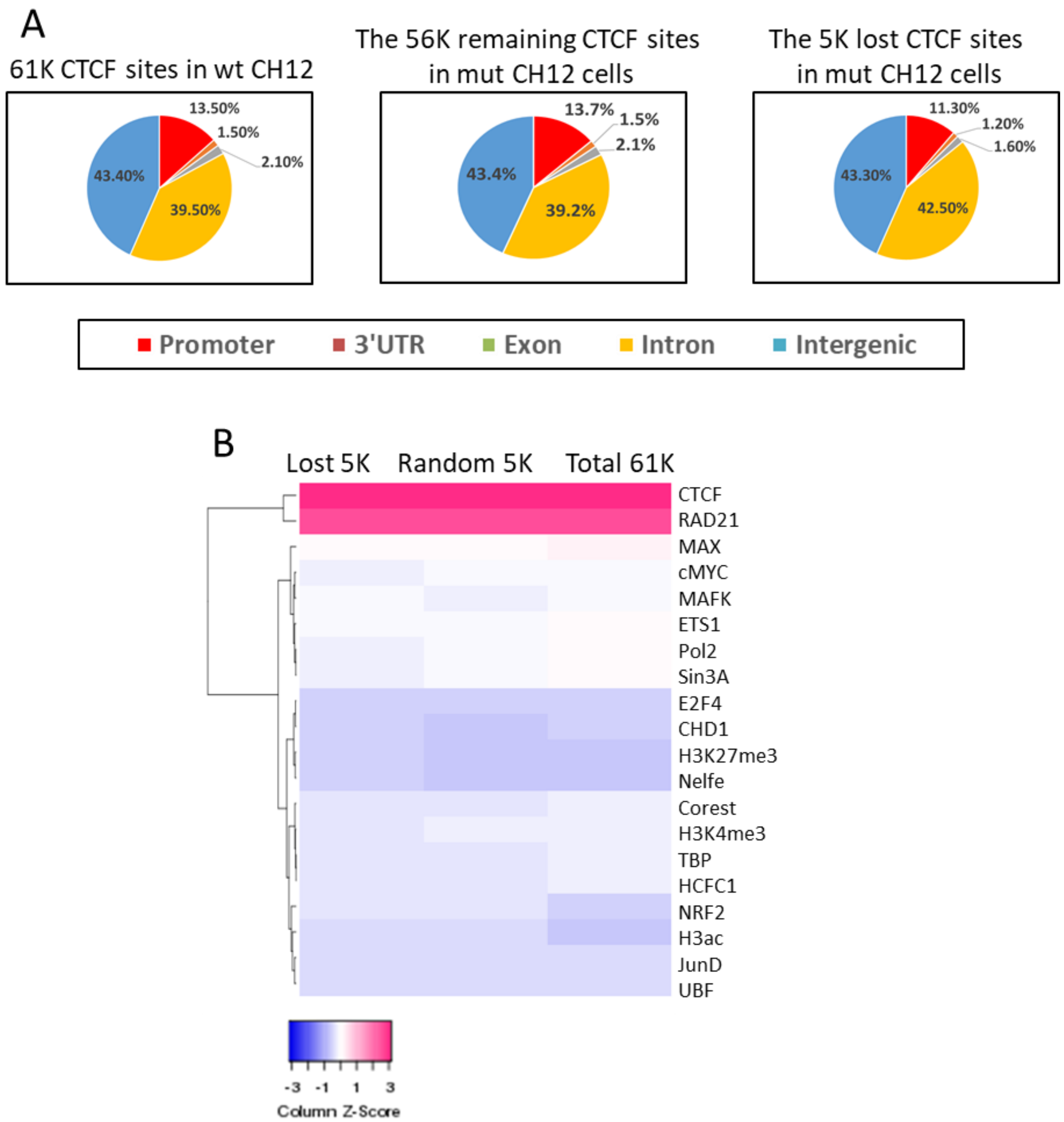

Fig. S5. Comparison of the 5K lost CTCF sites with all CTCF sites mapped in CH12 cells with respect to their genomic distribution and to their association with epigenetic marks and transcription factors (A) Genomic distribution of the remaining (56K) and the lost (5K) CTCF ChIP-seq peaks in mut CH12 cells in comparison with the genomic distribution of all CTCF peaks (61K) mapped in wt CH12 cells (left). The lost 5K sites and the remaining 56K CTCF sites showed a similar distribution with respect to genomic context. **(B)** A heatmap showing row z-scores of overlapping ChIP-seq data for multiple transcription factors and histone modifications (labeled at the right of the heatmap) with the 5K lost CTCF sites (Lost 5K), with the 5K sites randomly selected from a total number of 61K CTCF sites mapped in wt CH12 cells (Random 5K), and with the total number of all 61K CTCF sites mapped in wt CH12 cells (Total 61K).

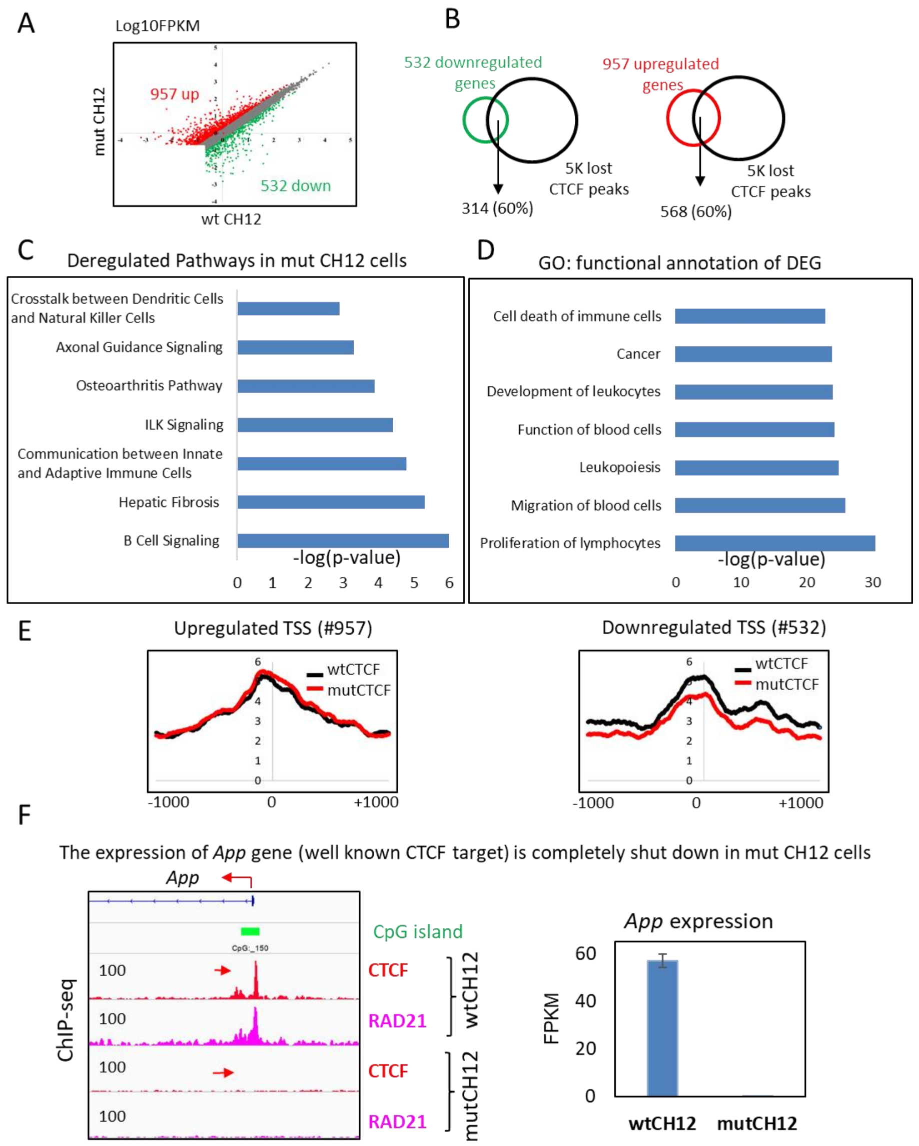

Fig. S6. The loss of the 5K CTCF sites in mut CH12 cells results in a significant deregulation of 1489 genes compared to wt CH12 cells. (A) Scatter plot of transcripts significantly ($p<0.005$) upregulated (red dots) and downregulated (green dots) in CH12 mutant cells compared to wt CH12 cells. **(B)** Venn diagrams representing overlap of CTCF ChIP-seq data with RNA-seq data in wt and mut CH12 cells. The genes that significantly changed expression upon deletion of ZFs 9-11 in CTCF **(A)** overlapped with CTCF peaks lost in mut CH12 cells. Gene coordinates were extended 100 kb up- and downstream of their transcription start and end sites. The majority (60%) of deregulated genes had a lost CTCF peak within 100 kb, suggesting that they might be direct targets of CTCF. **(C)** The major pathways that are significantly deregulated in mut CH12 cells compared to wt CH12 cells. **(D)** GO functions annotation of 1489 Differentially Expressed Genes (DEG) in mut CH12 cells. **(E)** Average plot of CTCF occupancy at TSSs (2kb) of upregulated genes (left panel) and downregulated genes (right panel) in CH12 wild type (black) and mutant (red) cells. **(F)** Loss of CTCF binding to the promoter region of *App* gene results in loss of *App* expression. Left panel: Genome browser view showing CTCF and RAD21 occupancy by ChIP-seq for wt and mut CH12 cells. The red arrows show loss of CTCF occupancy at the promoter of *App* gene in mut CH12 cells compared to wt CH12 cells. Right panel: *App* expression determined by RNA-seq data in wt CH12 and mut CH12 cells, FPKM (Fragments Per Kilobase Million).

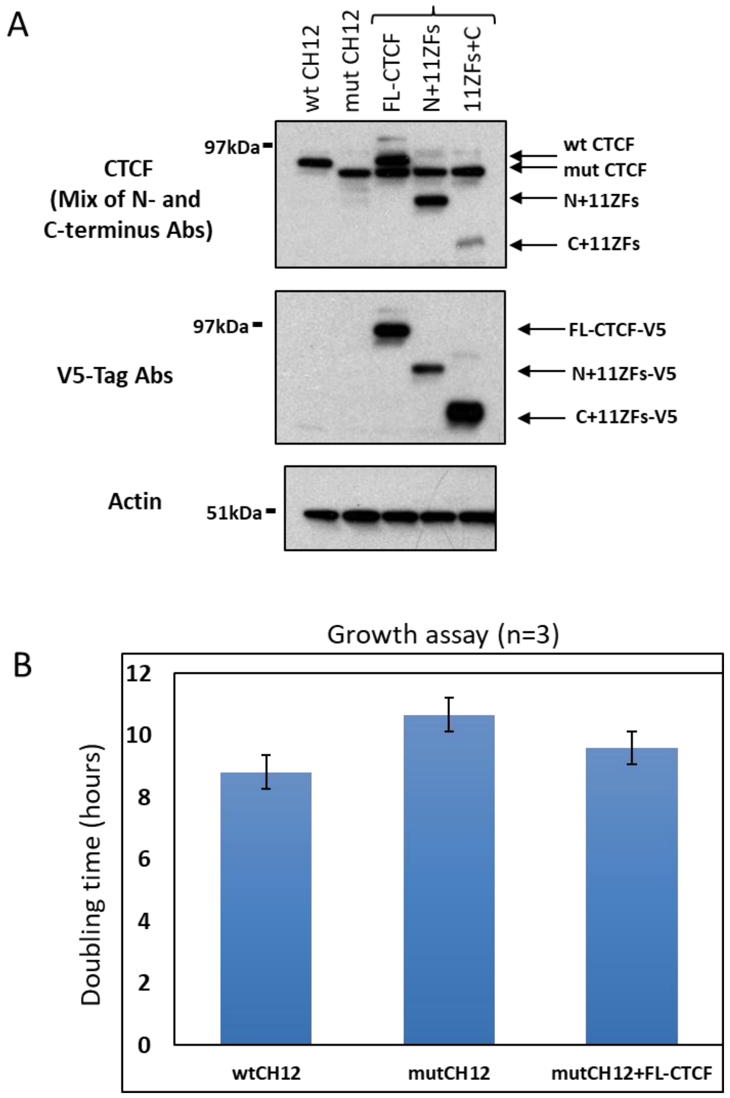

Fig. S7. Stable ectopic expression of different CTCF constructs tagged with V5 in mut CH12 cells. (A) Expression of Full-Length (FL) CTCF and CTCF with either C- (N+11ZFs) or N- (11ZFs+C) terminal truncations in mut CH12 cells was confirmed by Western blot with CTCF and V5-Tag Abs. **(B)** Growth assay for wt CH12, mut CH12, and mut CH12+FL-CTCF cells, error bars indicate mean of three independent experiments.

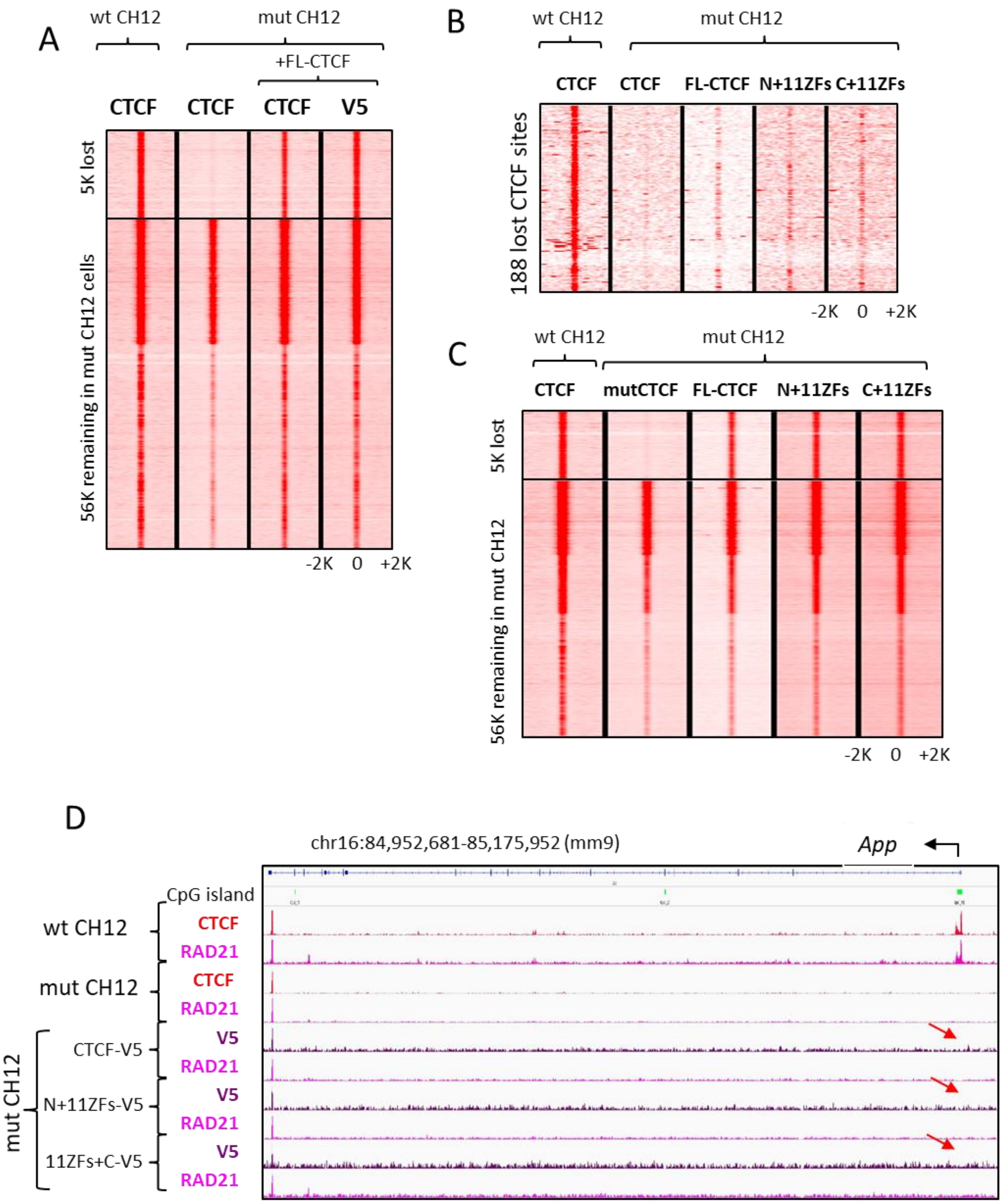

Fig. S8. Ectopically expressed CTCF constructs restore CTCF occupancy at the majority of CTCF sites lost in mut CH12 cells. (A) Heatmaps demonstrating that V5-tagged full-length (FL) CTCF restores CTCF occupancy at the 5K lost sites in mut CH12 cells**.** FL-CTCF was mapped by ChIP-seq with both CTCF and V5-tag Abs, shown at the top of the heatmap in comparison with CTCF occupancy in both wt and mut CH12 cells. **(B)** Heatmaps showing 188 lost CTCF sites that do not restore occupancy upon ectopic expression of CTCF in mut CH12 cells. **(C)** Heatmaps demonstrating that the binding pattern of FL-CTCF and truncated mutants in mut CH12 cells generally reproduced that of full-length CTCF, including the occupancy at the 5K lost CTCF sites. **(D)** Genome browser view of CTCF, V5, and RAD21 ChIP-seq data mapped in wt and mut CH12 cells. The *App* promoter, residing in a CpG island (green track), contains one of the 188 “permanently” lost CTCF sites **(B)** (shown by red arrows).

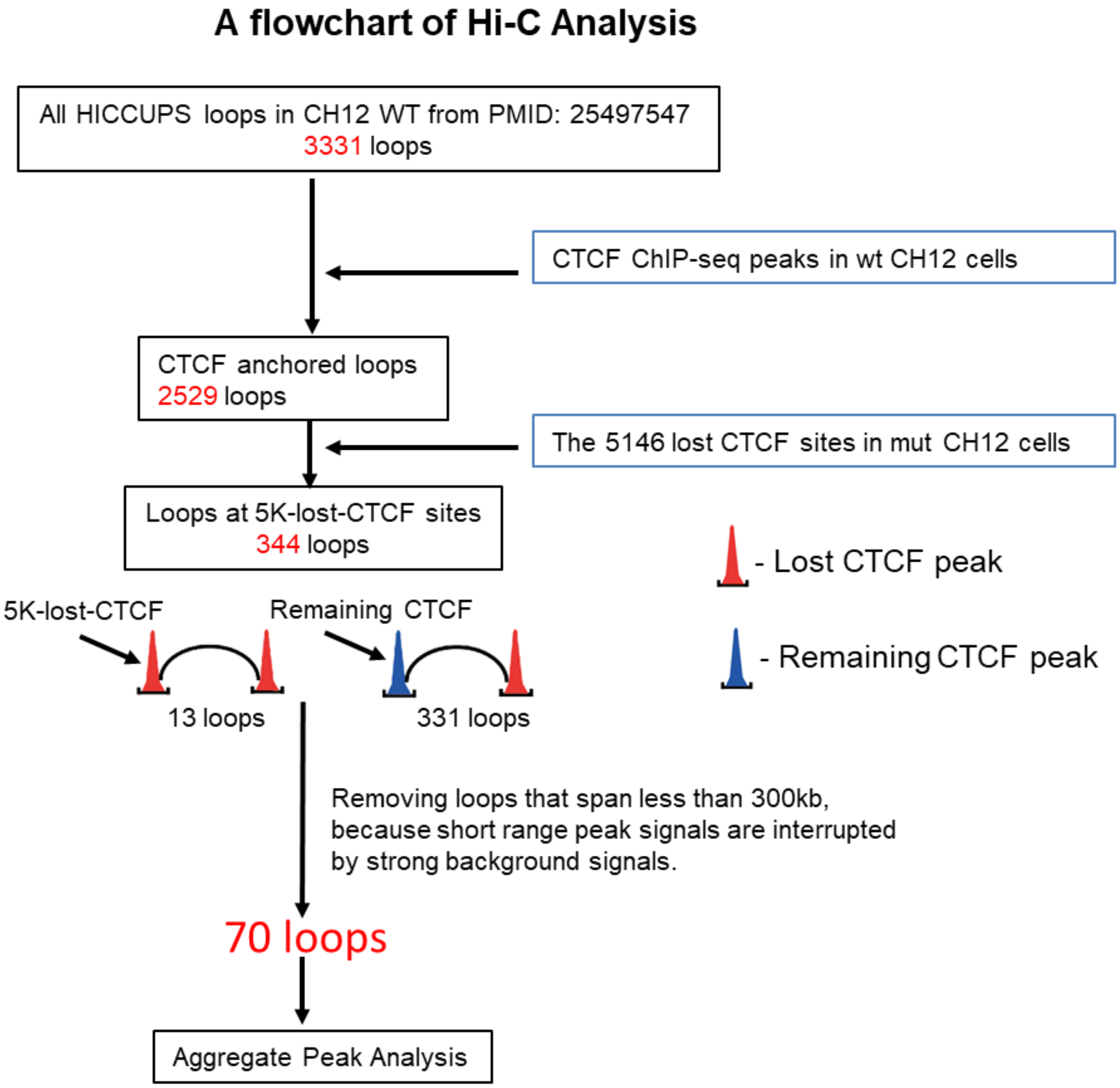

Fig. S9. Schematic representation of the chromatin loop selection for Hi-C analysis for the main Fig.4. First, we selected 2529 CTCF anchored chromatin loops by overlapping CTCF ChIP-seq data with a deeply sequenced Hi-C dataset where 3331 chromatin loops were identified in wt CH12 cells (PMID: 25497547). Second, we selected 344 loops that overlapped with the 5K lost CTCF sites at one or both anchors. Third, we sorted out 70 loops for Hi-C analysis by removing the short-range loops (those that span less than 300kb).

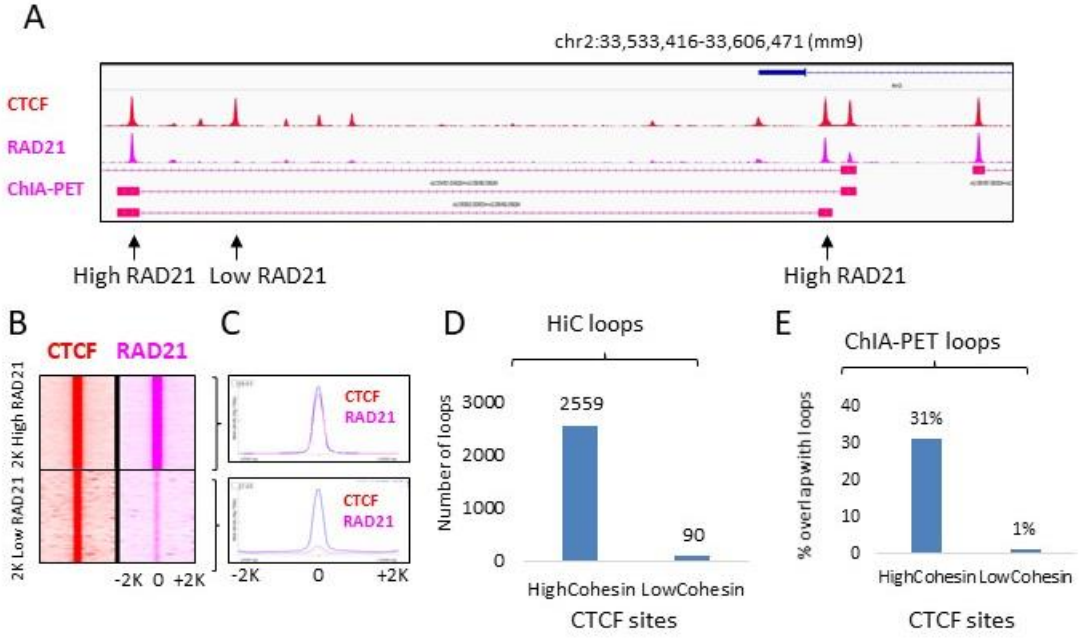

Fig. S10. CTCF sites depleted of cohesin occupancy are not generally involved in anchoring of chromatin loops. (A) Genome browser view of CTCF (red) and RAD21 (pink) ChIP-seq occupancy shown in combination with ChIA-PET anchors mapped with CTCF Abs in wt CH12 cells. The CTCF sites involved in anchoring chromatin loops are associated with robust cohesin peaks (shown by black arrows – High RAD21), while CTCF sites depleted of cohesin occupancy (Low RAD21) are not involved in chromatin loop formation. **(B)** Heatmaps showing two classes of CTCF sites with high and low enrichment of cohesin (RAD21) in wt CH12 cells. **(C)** Average CTCF and RAD21 ChIP-seq tag density at the two classes of CTCF sites from **(B)**. **(D)** Bar plot showing the number of Hi-C loops overlapping with the same number (2K) of CTCF sites with high and low cohesin occupancy (from panel B). **(E)** Bar plot showing the percent overlap between CTCF sites with high and low cohesin occupancy and anchors of chromatin loops mapped by CTCF ChIA-PET.

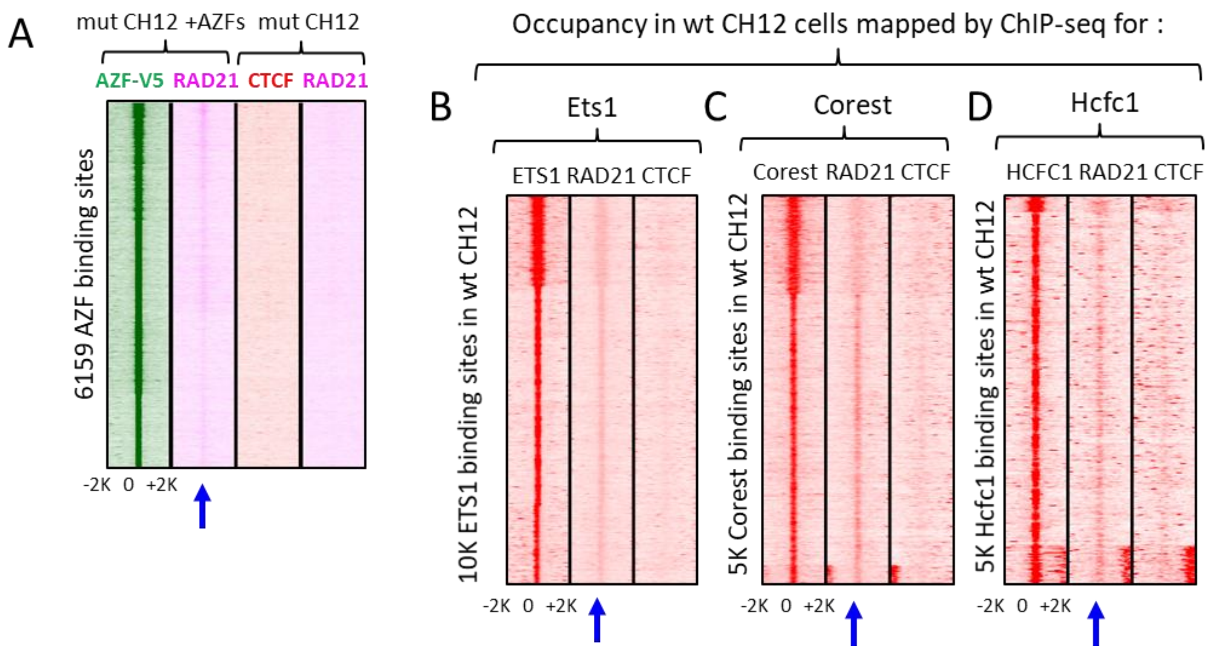

Fig. S11. A slight enrichment of cohesin (RAD21) occupancy at the binding sites of factors that are not involved in cohesin retention onto chromatin. **(A)** Heatmap of AZF, CTCF, and RAD21 occupancy at 6,159 AZF binding sites that do not overlap with CTCF binding sites. The blue arrow indicates the slight enrichment of RAD21 occupancy at the new AZF binding sites that is not present in parental mut CH12 cells. **(B-D)** Heatmap of CTCF and RAD21 occupancy at the binding sites of Ets1 **(B)**, CoRest **(C)** and Hcfc1**(D)** transcription factors that do not overlap with CTCF binding sites in CH12 cells. The blue arrow indicates the slight enrichment of RAD21 occupancy at the transcription factor binding sites.

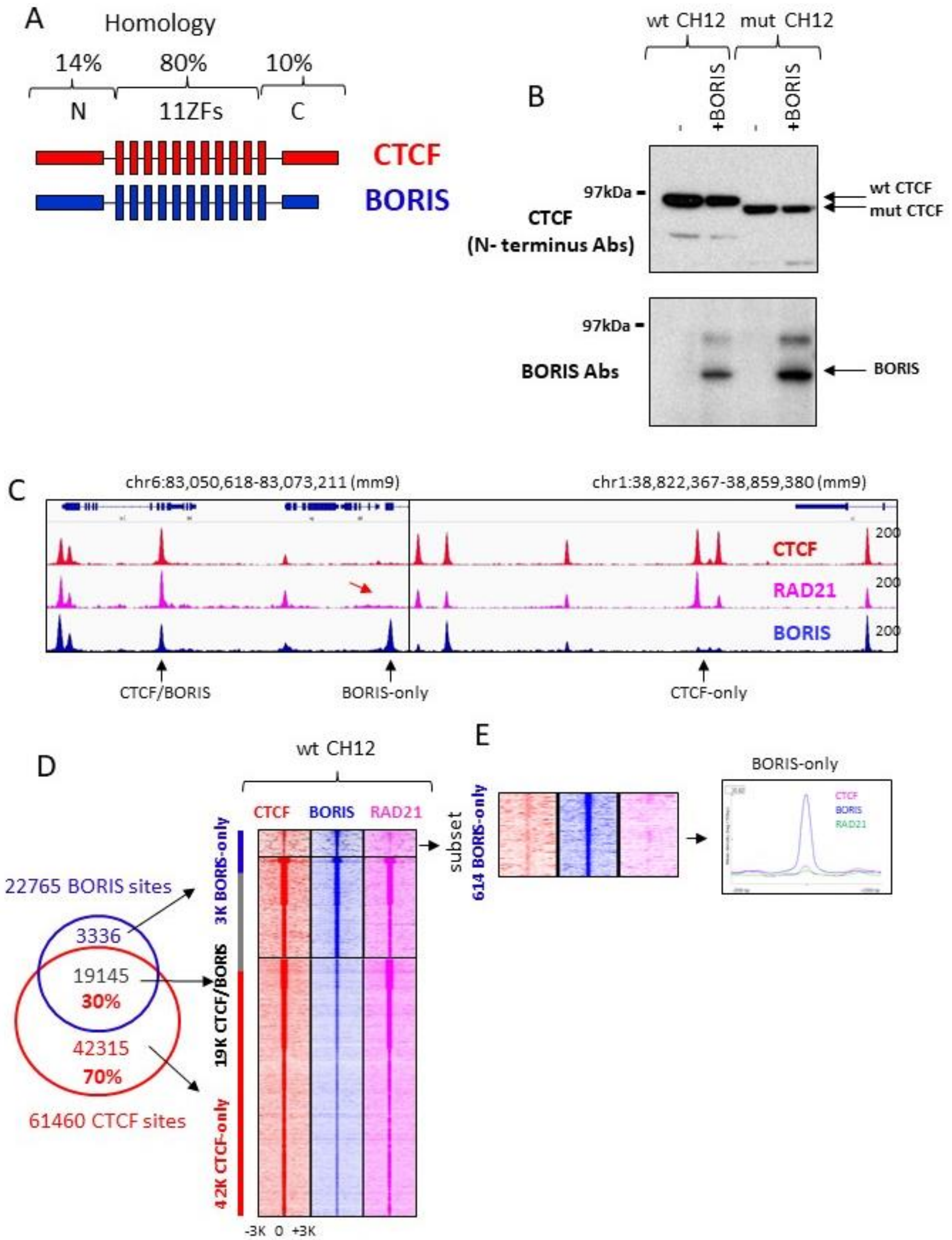

Fig. S12. BORIS-only sites are depleted of cohesin. (A) Schematic representation CTCF and BORIS proteins. The percent homology between the two proteins within the main domains (N-terminus, 11ZFs, C-terminus) is indicated. **(B)** Western blot showing stable ectopic BORIS expression in wt and mutant CH12 cells. **(C)** Genome browser view of CTCF (red), RAD21 (pink), and BORIS (blue) binding in wt CH12 cells stably expressing ectopic BORIS. Selective CTCF and BORIS occupancy is indicated by black arrows. The absence of cohesin at a BORIS-only site is shown by a red arrow. **(D)** Venn diagram and heatmaps of CTCF (red), BORIS (blue) and RAD21 (pink) occupancy in wt CH12 cells. **(E)** Heatmap and average plot showing that BORIS-only sites are generally depleted of cohesin occupancy. As relatively low CTCF and RAD21 occupancies could be detected at some of BORIS-only sites, we selected 614 sites completely depleted of CTCF occupancy (a subset of BORIS-only sites) and additionally analyzed them for cohesin occupancy.

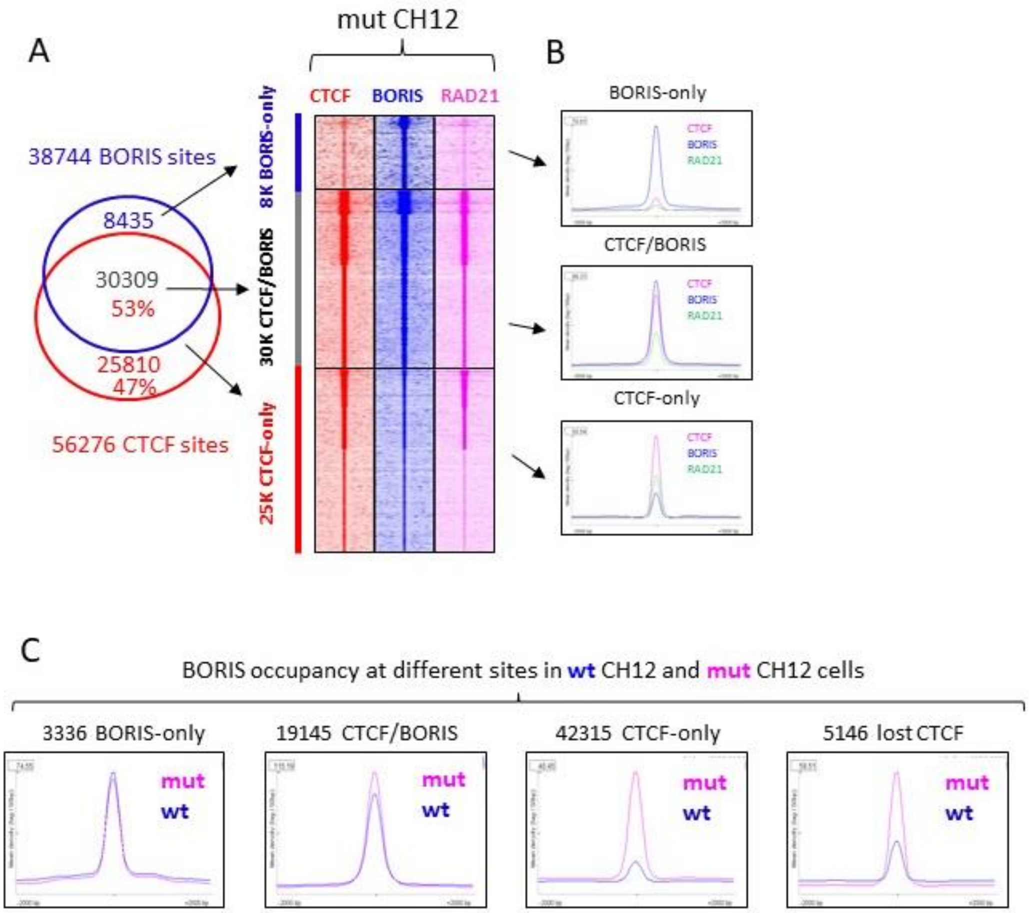

Fig. S13. BORIS outcompetes mutant CTCF in mut CH12 cells more efficiently than wild type CTCF in wt CH12 cells. (A) Venn diagram showing overlap of CTCF (red) and BORIS (blue) occupancy in mut CH12 cells. Heatmap shows CTCF (red), BORIS (blue) and RAD21 (pink) occupancy at regions selectively bound by CTCF and BORIS. **(B)** Average plots show that RAD21 occupancy follows CTCF but not BORIS occupancy, as BORIS-only sites are completely depleted of cohesin occupancy. The connections between panels A and B are shown by black arrows. **(C)** Average plots of BORIS occupancy at different classes of sites in wt and mut CH12 cells demonstrate an increase in BORIS occupancy at the majority of sites in mut CH12 cells.

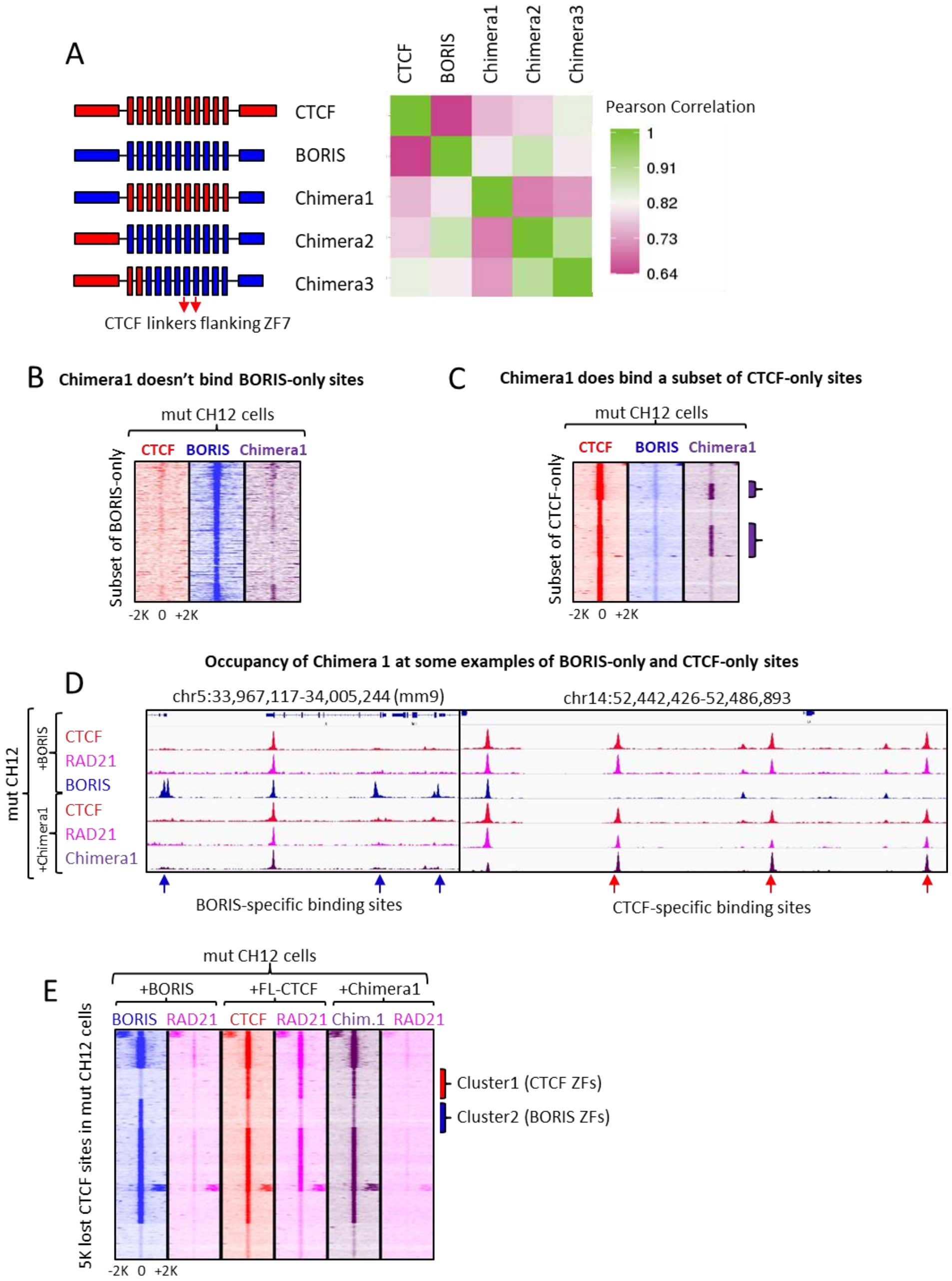

Fig. S14. Without the N-terminus of CTCF, the CTCF ZFs bound to CTCF target sites are not sufficient for cohesin retention. (**A**) Left: schematic representations of CTCF, BORIS, and chimeric proteins expressed in mut CH12 cells. Right: heatmap representing Pearson's correlations of CTCF, BORIS and Chimeric protein occupancy at a combined set of CTCF and BORIS target sites mapped in mut CH12 cells. **(B, C)** Heatmaps demonstrating a moderate degree of divergence in the sequence specificity of CTCF and BORIS ZFs. In particular, BORIS with CTCF ZFs (Chimera1) lost the ability to bind BORIS-only sites **(B)** and gained the ability to bind some CTCF-only sites **(C)**, respectively. **(D)** Genome browser view of Chimera1 binding in comparison to BORIS occupancy in mut CH12 cells. **(E)** Heatmap of BORIS (blue), CTCF (red), RAD21 (pink), and Chimera1 (purple) occupancy at the 5K lost CTCF sites in mut CH12 cells demonstrates an inability of Chimera1 to recruit cohesin (RAD21). Two clusters of CTCF binding sites differentially occupied by CTCF and BORIS ZFs are highlighted by brackets.

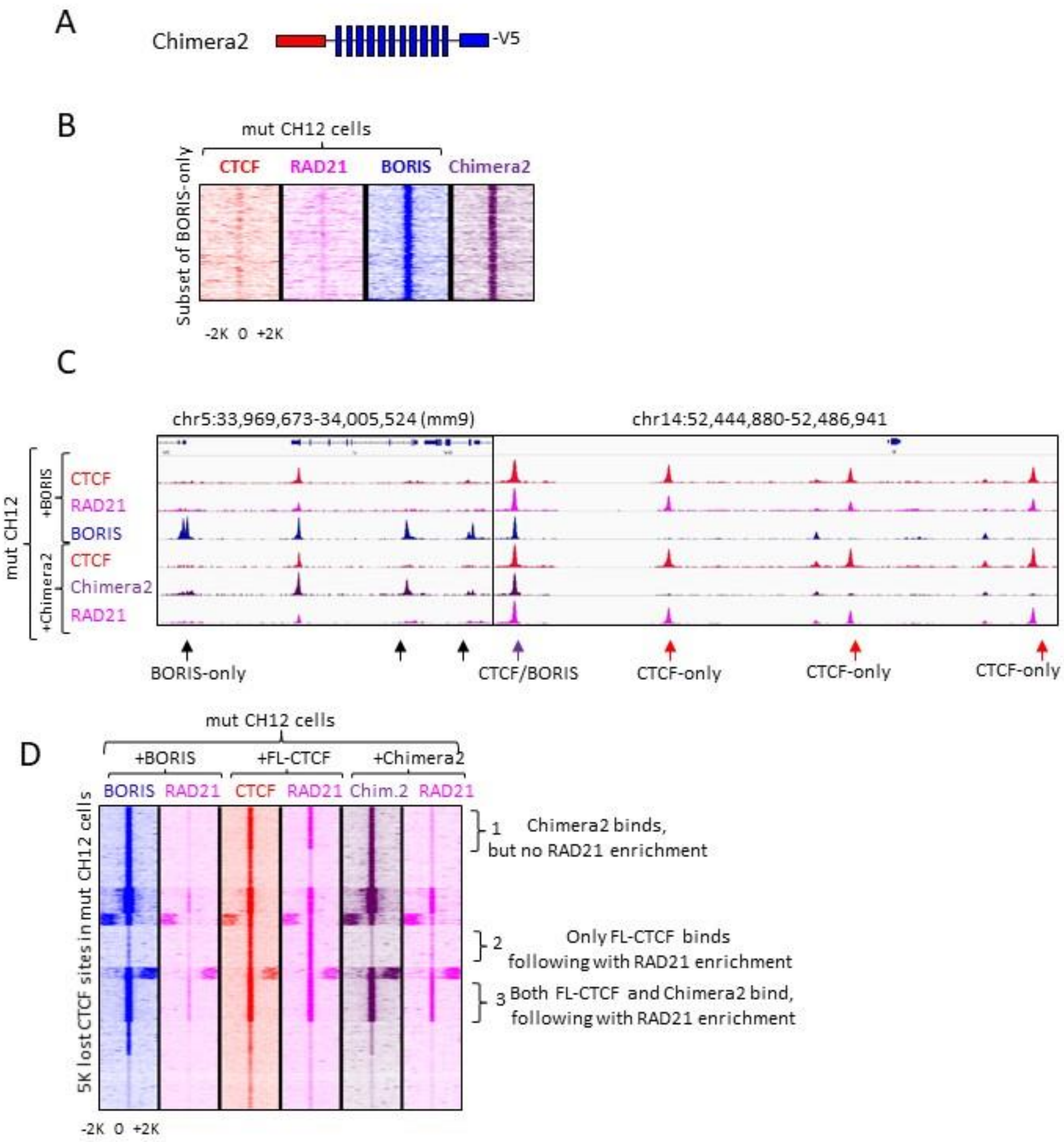

Fig. S15. BORIS with the N-terminus of CTCF (Chimera2) is able to retain cohesin, but less efficiently than FL-CTCF. (A) A schematic representation of Chimera2. **(B)** Heatmaps of BORIS (blue), CTCF (red), RAD21 (pink), and Chimera2 (purple) enrichment show that Chimera2 binds BORIS-only binding sites but does not induce cohesin retention. **(C)** Genome browser occupancy of Chimera2 demonstrates a binding pattern more similar to that of BORIS than CTCF. **(D)** Heatmaps of BORIS (blue), CTCF (red), RAD21 (pink), and Chimera2 (purple) occupancy at the 5K lost CTCF sites demonstrates an overall gain of cohesin occupancy following the gain of Chimera2 occupancy, albeit to a lower extent than with FL-CTCF stably expressed in mut CH12 cells. K-means ranked clustering of ChIP-seq data along the 5K lost CTCF sites shows that only some of them were enriched with cohesin, reflecting Chimera2 occupancy, while the majority of the lost CTCF sites were occupied by cohesin following FL-CTCF occupancy. Clusters shown on the right side of heatmap explain the observed patterns.

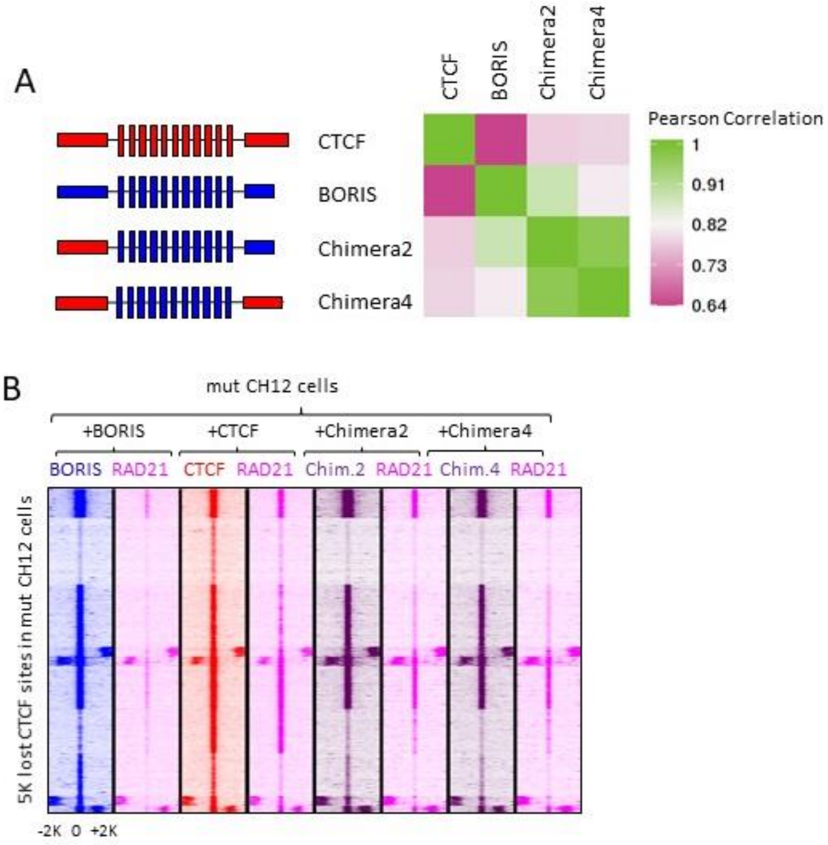

Fig. S16. Replacement of CTCF ZFs with BORIS ZFs (Chimera4) reduces the ability of CTCF to recruit cohesin. (A) Left: a schematic representation of Chimera2 and Chimera4 in comparison with CTCF and BORIS. Right: heatmap representing Pearson's correlation of CTCF, BORIS, Chimera2, and Chimera4 occupancy at a combined set of CTCF and BORIS target sites mapped in mut CH12 cells. **(B)** Heatmaps showing that Chimera2 and Chimera4 are very similar with respect to their binding profile and cohesin retention capability.

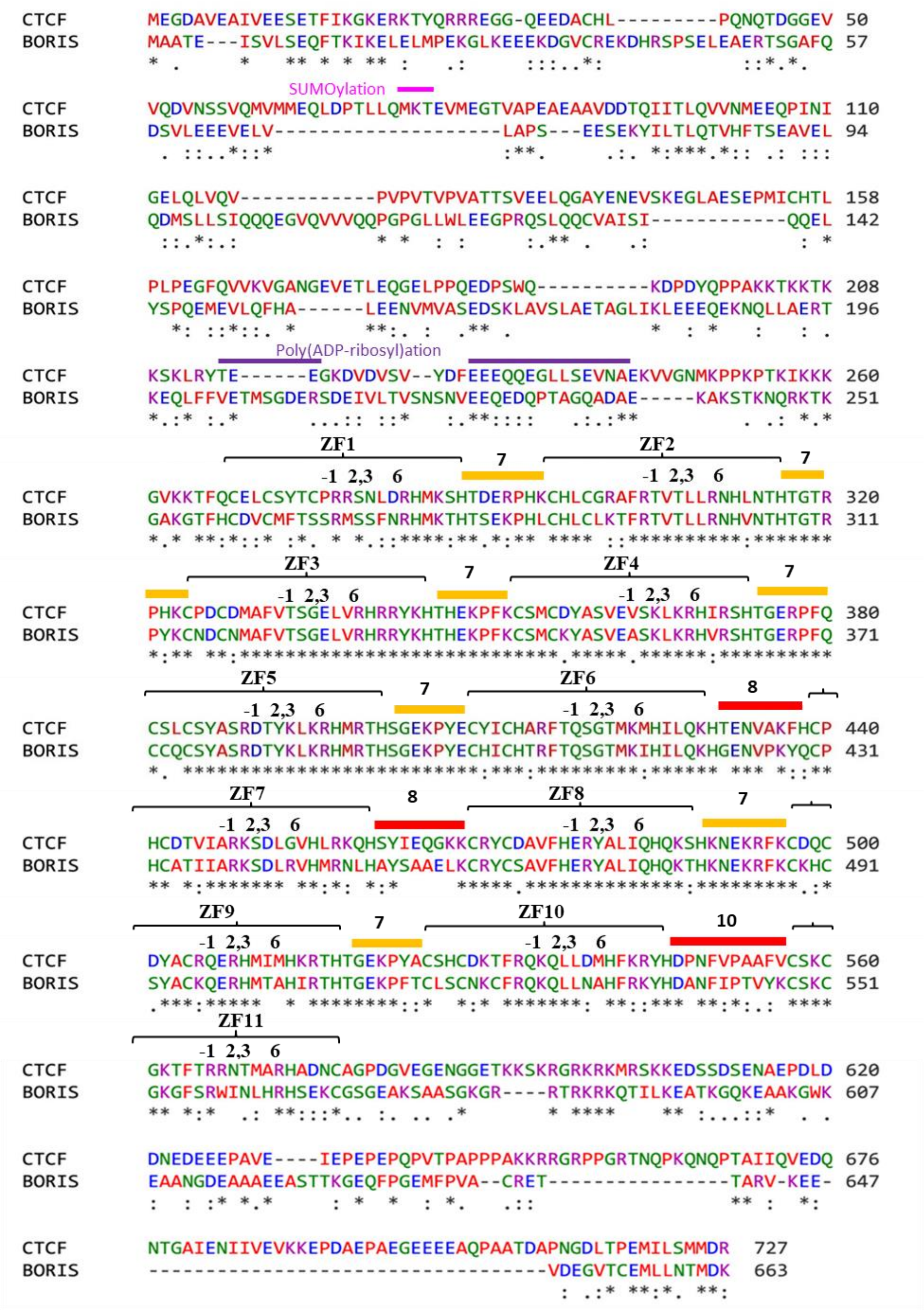

Fig. S17. ClustalW alignment of CTCF and BORIS amino acid sequences. '* ' indicates the residues in that column are identical in the alignment, ':' indicates conserved substitutions, and '.' indicates semi-conserved substitutions. Red- (Small, Hydrophobic, Aromatic (not-Y)), Blue- (Acidic), Magenta-(Basic), Green- (Hydroxyl, Amine, Basic-Q). The yellow and red blocks represent canonical and noncanonical linkers, respectively. The numbers at the top of blocks between ZFs show linker length. The numbers (-1,2,3,6 position of the alpha-helical region of the zinc-finger domain) show the four essential amino acids involved in DNA recognition for each ZF. The regions of SUMOylation and Poly(ADP-ribosyl)ation, (reported in PMID: 19029252 and 15361875) are labelled at the top of the alignment.

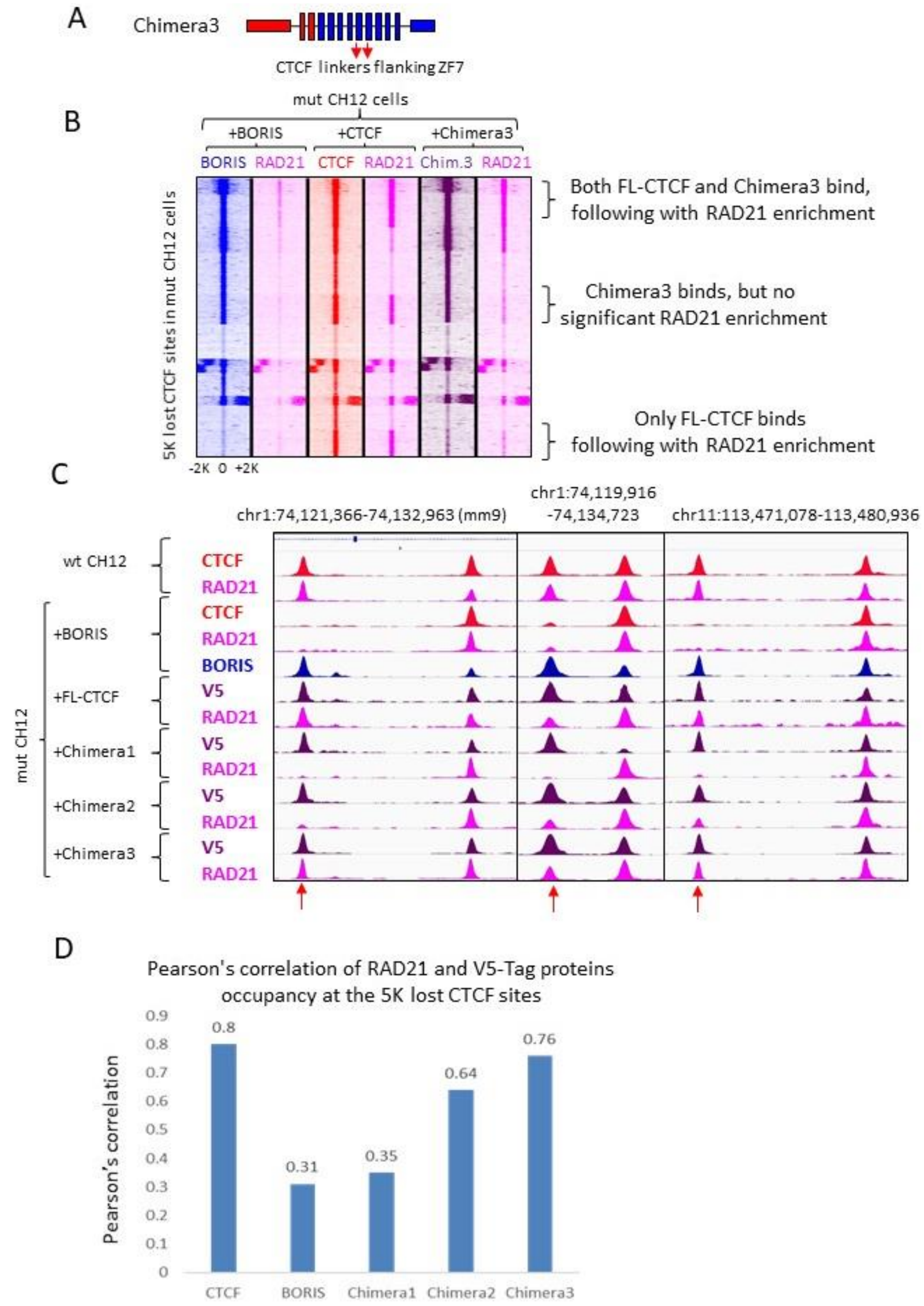

Fig. S18. Chimera3 is able to retain cohesin but less efficiently than FL-CTCF. (**A)** A schematic representation of Chimera3. **(B)** Heatmaps of BORIS (blue), CTCF (red), RAD21 (pink), and Chimera3 (purple) occupancy at the 5K lost CTCF sites demonstrate an overall gain of cohesin occupancy following the gain of Chimera3 occupancy. K-means ranked clustering of ChIP-seq data along the 5K lost CTCF sites shows different clusters of Chimera3 and RAD21 occupancy. Clusters shown on the right side of heatmap explain the observed patterns**. (C)** Genome browser view of CTCF (red), RAD21 (pink), BORIS (blue), and chimeric protein (purple) binding in wt and mut CH12 cells. Red arrows indicate occupancy at lost CTCF sites. **(D)** Pearson's correlation of RAD21 and chimeric protein occupancy at the 5K lost CTCF sites.

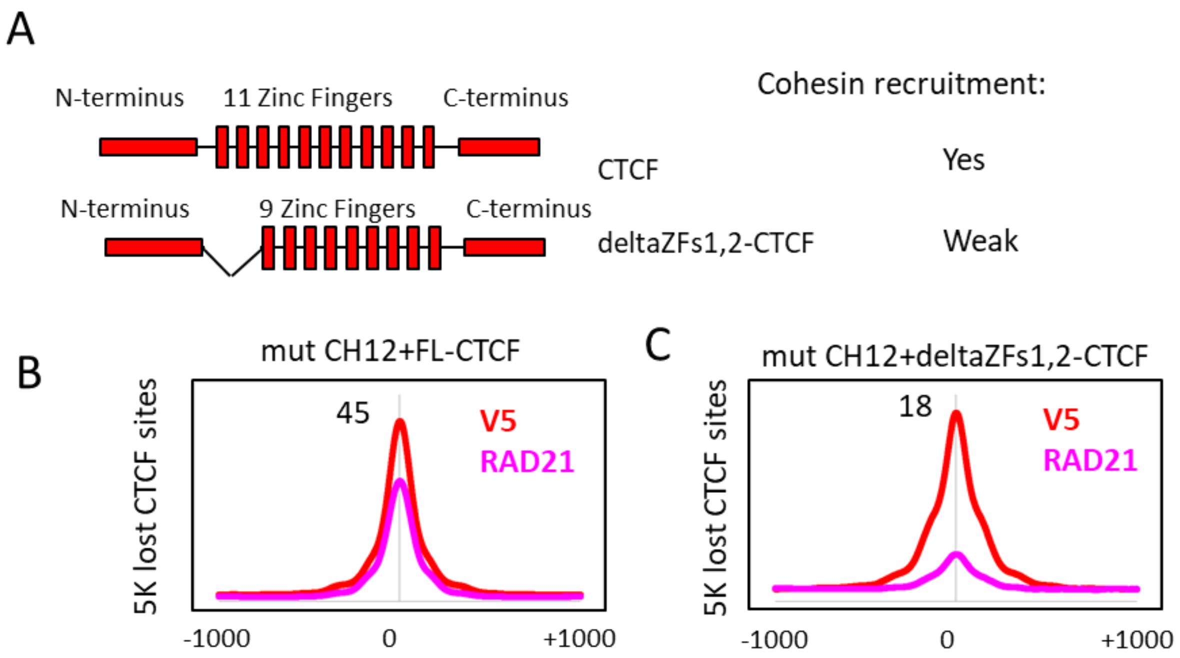

Fig. S19. The first two CTCF ZFs are involved in cohesin retention (A) Left: a schematic representation of wt CTCF and CTCF with the first two ZFs deleted. Right: a summary of cohesin recruitment by corresponding proteins from the left. **(B, C)** Average profiles of V5 and RAD21 occupancy at the 5K lost CTCF binding sites mapped in mut CH12 cells stably expressing either FL-CTCF **(B)** or CTCF with the first 2 ZFs deleted (deltaZFs1,2-CTCF). **(C)** Deletion of the first two ZFs in CTCF severely affects cohesin retention at the 5K lost CTCF sites as compared to FL-CTCF expressed in mut CH12 cells.

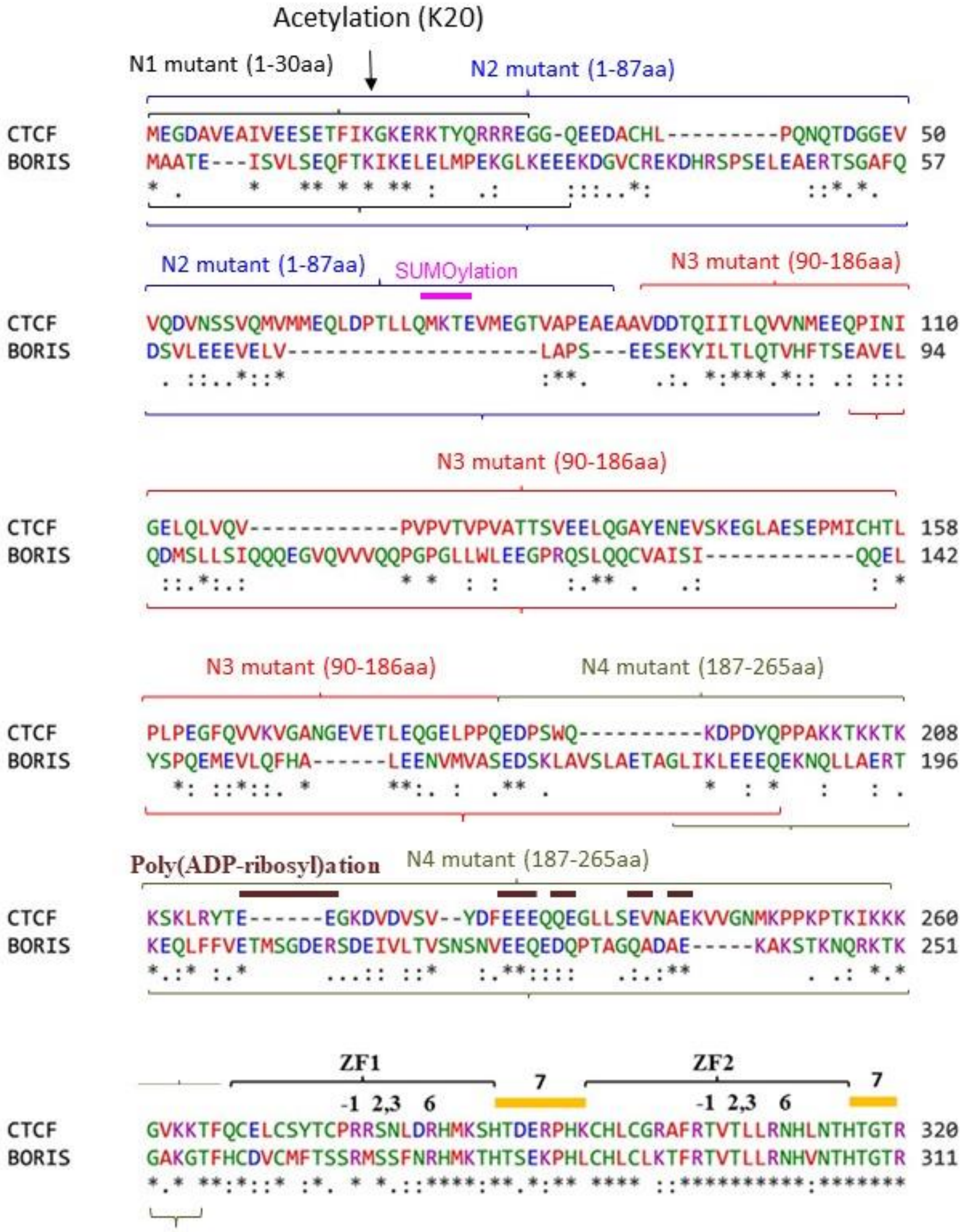

Fig. S20. Strategy for replacement of CTCF aa sequences with corresponding BORIS aa sequences to generate four N-terminal CTCF mutants. Alignment of the N-termini of CTCF and BORIS proteins. '* ' indicates the residues identical in the alignment, ':' indicates conserved substitutions, '.' indicates semi-conserved substitutions. Red-(Small, Hydrophobic, Aromatic (not-Y)), Blue-(Acidic), Magenta-(Basic), Green-(Hydroxyl, Amine, Basic-Q). The known posttranslational modifications of the N-terminus of CTCF are marked. The N-terminal substitutions of aa sequences of CTCF by aa sequences of BORIS are shown by differently colored brackets. For example, in N1 mutant the 30aa sequence of CTCF (shown by black bracket on the top of CTCF sequence) is substituted with the corresponding 30aa sequence of BORIS (shown by the black bracket on the bottom of BORIS sequence). The same strategy was applied to generate the N2 mutant (blue), N3 mutant (red), and N4 mutant (Green). The positions of acetylation, SUMOylation and Poly(ADP-ribosyl)ation (reported in PMID: 19029252, 15361875) are labelled at the top of the alignment.

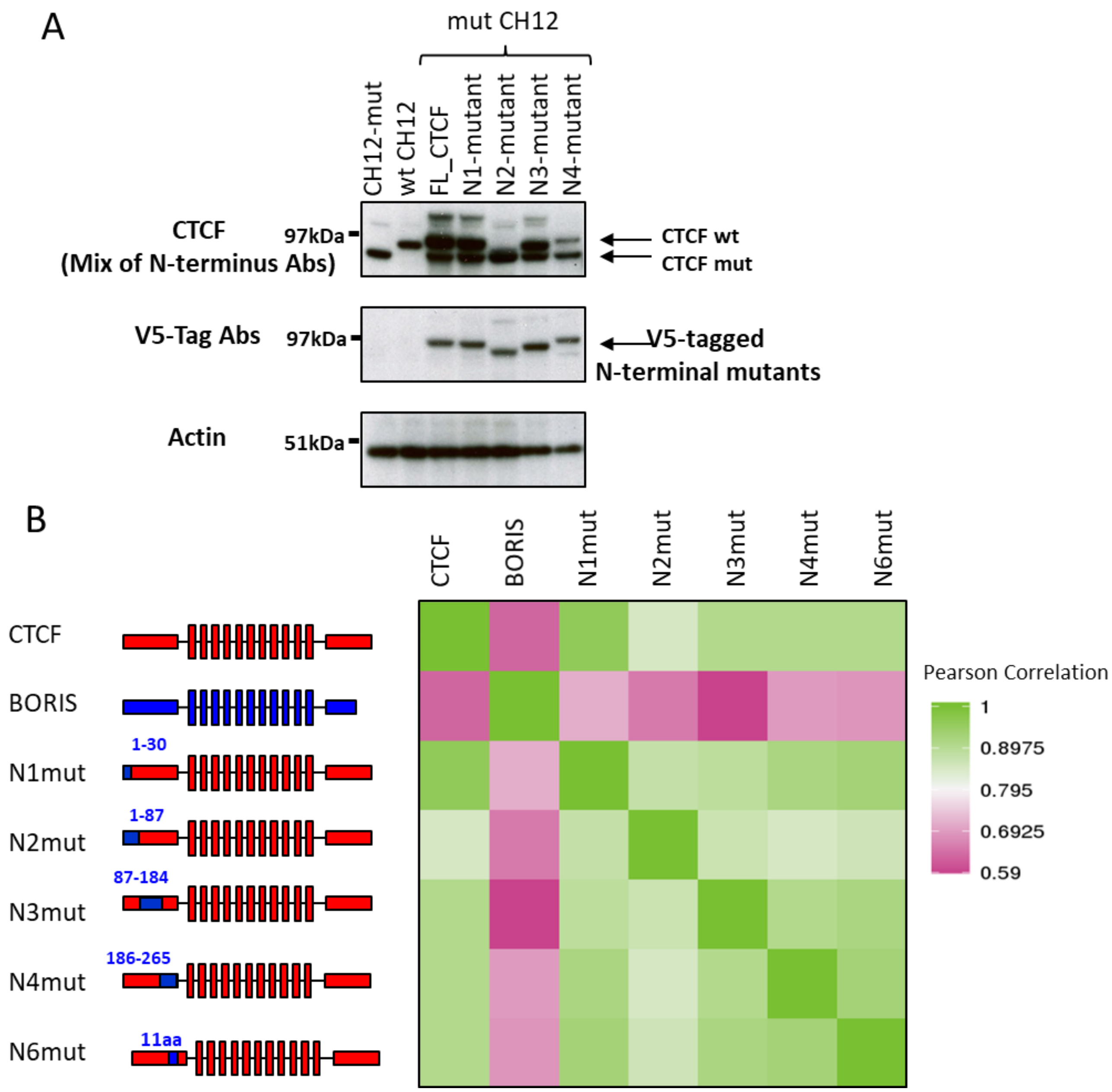

Fig. S21. Stable ectopic expression of N-terminal CTCF mutants in mut CH12 cells. (A) Western blot showing expression of N-terminal CTCF mutants in mut CH12 cells compared to wild type CTCF in wt CH12 cells. **(B)** Heatmap representing Pearson's correlation of CTCF, BORIS, and N-terminal CTCF mutants' occupancy at a combined set of CTCF and BORIS target sites mapped in mut CH12 cells.

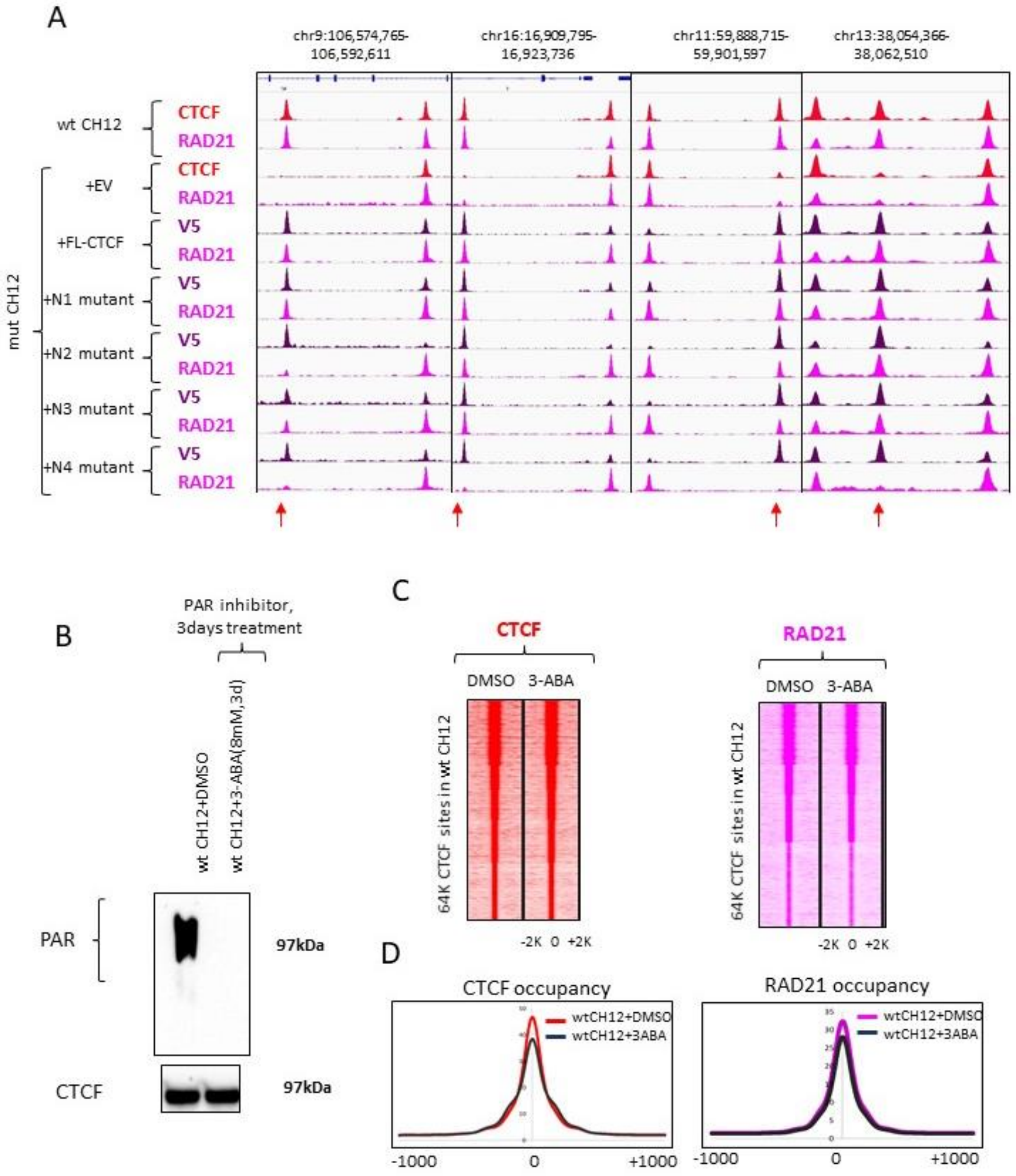

Fig. S22. 79 amino acids in the N-terminus of CTCF (N4-mutant) are necessary for cohesin retention at CTCF target sites. (A) Genome browser view of CTCF (red), RAD21 (pink) and N-terminally V5-tagged mutant protein (purple) binding in wt and mut CH12 cells. Arrows indicate occupancy at lost CTCF sites in mut CH12 cells. **(B)** wt CH12 cells were cultured with either DMSO (Negative control) or with the PARP-1 inhibitor 3-aminobenzamide (3-ABA, 8 mM) for 3 days, with a change of media every day. After 3 days of culture, cells were harvested, and PAR levels were analyzed by immunoblot using mouse monoclonal anti-PAR Abs (clone 10H, Abcam, ab14459). Verification of equivalent protein levels was provided by the immunoblot detection of CTCF. **(C)** Heatmaps show CTCF (red) and RAD21 (pink) occupancies at CTCF binding sites in wt CH12 cells treated either with DMSO or 3-ABA. We observed a slight decrease of CTCF occupancy followed by a slight decrease of RAD21 occupancy at CTCF sites in the cells treated with the PARP-1 inhibitor (3-ABA) compared to DMSO. (D) Average plots of CTCF and RAD21 occupancy in wt CH12 cells treated either with DMSO or 3-ABA corresponding to the heatmaps in (C).

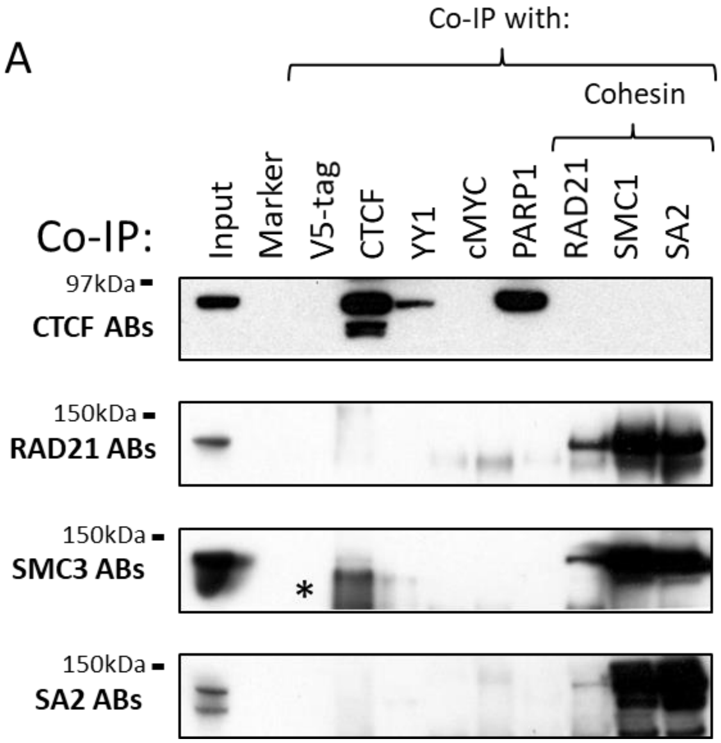

Fig. S23. Co-immunoprecipitation (IP) studies of CTCF and cohesin in wt CH12 cells. No co-immunoprecipitation of CTCF with any cohesin subunits was detected when DNA-assisted protein interactions were inhibited by ethidium bromide. V5-tag and cMYC Abs were used as a negative control. YY1 and PARP1 Abs were used as a positive control for CTCF. All four cohesin subunits (RAD21, SMC1, SMC3, and SA2) are co-immunoprecipitated together. The asterisk shows a nonspecific band, not corresponding to the molecular weight of SMC3 protein.

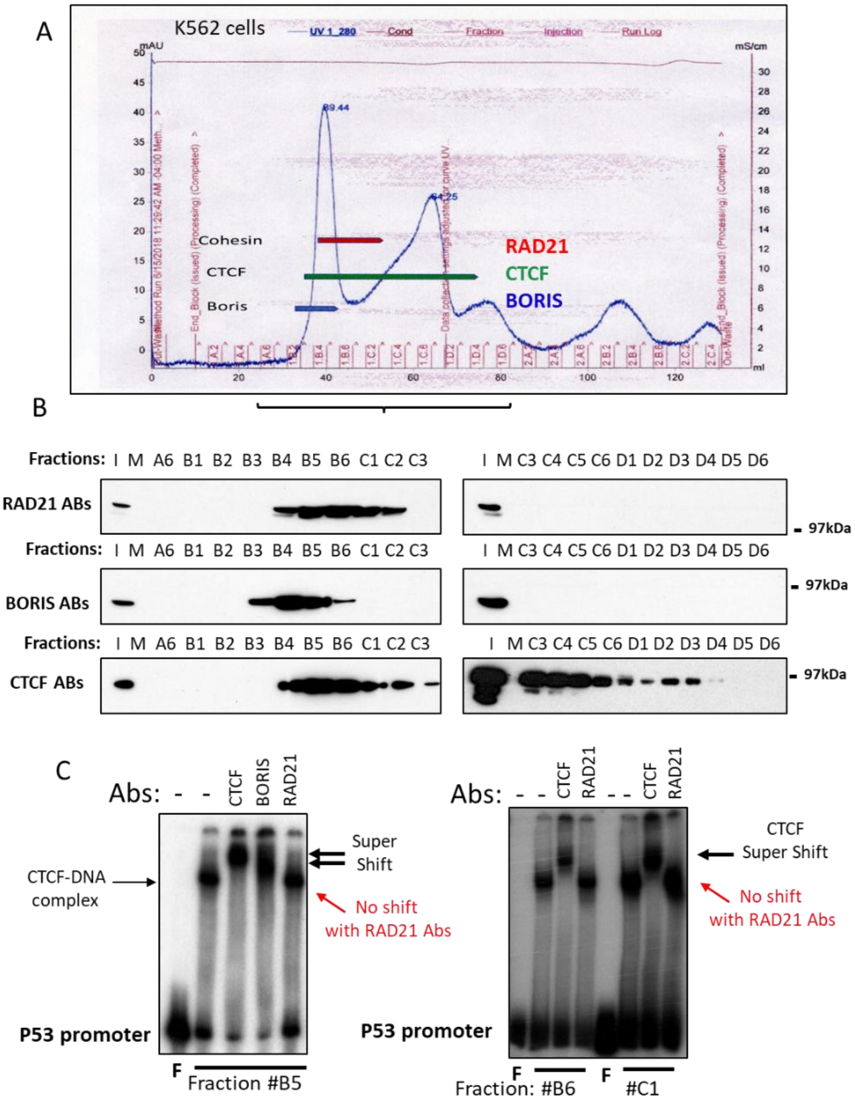

Fig. S24. CTCF and cohesin are not present in one complex in nuclear extracts. **(A)** K562 nuclear extracts (NUN) were separated by size exclusion chromatography on Sephacryl 300 column, producing a total of 44 fractions. The OD_{280} profile shown with the fraction numbers. The red (RAD21), green (CTCF) and blue (BORIS) lines show the summary of panel **(B)**: the presence of the corresponding proteins in the corresponding nuclear fractions. **(B)** Western blot analysis of RAD21, BORIS and CTCF proteins in NUN fractions after size exclusion chromatography. (I) – Input, (M) - Marker, the numbers (A6-D6) correspond to the fractions in **(A).** While CTCF protein was present in 14 high molecular weight fractions, cohesin protein (RAD21) was present only in 5 of these fractions. If cohesin forms the long-lasting complexes with CTCF, one should expect to detect a full overlap of CTCF-cohesin complexes in all 14 fractions. This data suggests the absence of stable CTCF-cohesin interactions without DNA assistance. **(C)** Electrophoretic Mobility Shift Assay (EMSA) with some of CTCF-cohesin overlapping nuclear fractions (B5, B6, C1) to see if the proteins form a stable complex that can be supershifted with both CTCF and RAD21 Abs. EMSA with CTCF-cohesin overlapping nuclear extract fractions demonstrated that the labelled DNA-protein complexes could be super-shifted with antibodies against CTCF and BORIS, a known interacting partner of CTCF, but not with antibodies against cohesin subunit RAD21, thus confirming the absence of CTCF-cohesin complexes in the nuclear extracts, consistent with our co-IP results (Fig. S23). The P^{32}-labelled p53 promoter probe, described in (PMID: 26268681), was used in EMSA assay. The black arrows show the supershift with both CTCF and BORIS Abs, but not with RAD21 Abs (red arrows).

Open Reading Frames (ORFs) of all ectopically expressed constructs in this study

1. FL-CTCF-V5:
MEGDAVEAIVEESETFIKGKERKTYQRRREGGQEEDACHLPQNQTDGGEVVQDVNSSVQM
VMMEQLDPTLLQMKTEVMEGTVAPEAEAAVDDTQIITLQVVNMEEQPINIGELQLVQVPVP
VTVPVATTSVEELQGAYENEVSKEGLAESEPMICHTLPLPEGFQVVKVGANGEVETLEQGEL
PPQEDPSWQKDPDYQPPAKKTKKTKKSKLRYTEEGKDVDVSVYDFEEEQQEGLLSEVNAEK
VVGNMKPPKPTKIKKKGVKKTFQCELCSYTCPRRSNLDRHMKSHTDERPHKCHLCGRAFRTVT
LLRNHLNTHTGTRPHKCPDCDMAFVTSGELVRHRRYKHTHEKPFKCSMCDYASVEVSKLKRHIRS
HTGERPFQCSLCSYASRDTYKLKRHMRTHSGEKPYECYICHARFTQSGTMKMHILQKHTENVAKF
HCPHCDTVIARKSDLGVHLRKQHSYIEQGKKCRYCDAVFHERYALIQHQKSHKNEKRFKCDQCD
YACRQERHMIMHKRTHTGEKPYACSHCDKTFRQKQLLDMHFKRYHDPNFVPAAFVCSKCGKTFT
RRNTMARHADNCAGPDGVEGENGGETKKSKRGRKRKMRSKKEDSSDSENAEPDLDDNEDEEEP
AVEIEPEPEPQPVTPAPPPAKKRRGRPPGRTNQPKQNQPTAIIQVEDQNTGAIENIIVEVKKEPDAEP
AEGEEEEAQPAATDAPNGDLTPEMILSMMDRGSGSGS**GKPIPNPLLGLDST**

2. N-terminus+11ZFs-V5 (C-terminus deleted CTCF):
MEGDAVEAIVEESETFIKGKERKTYQRRREGGQEEDACHLPQNQTDGGEVVQDVNSSVQM
VMMEQLDPTLLQMKTEVMEGTVAPEAEAAVDDTQIITLQVVNMEEQPINIGELQLVQVPVP
VTVPVATTSVEELQGAYENEVSKEGLAESEPMICHTLPLPEGFQVVKVGANGEVETLEQGEL
PPQEDPSWQKDPDYQPPAKKTKKTKKSKLRYTEEGKDVDVSVYDFEEEQQEGLLSEVNAEK
VVGNMKPPKPTKIKKKGVKKTFQCELCSYTCPRRSNLDRHMKSHTDERPHKCHLCGRAFRTVT
LLRNHLNTHTGTRPHKCPDCDMAFVTSGELVRHRRYKHTHEKPFKCSMCDYASVEVSKLKRHIRS
HTGERPFQCSLCSYASRDTYKLKRHMRTHSGEKPYECYICHARFTQSGTMKMHILQKHTENVAKF
HCPHCDTVIARKSDLGVHLRKQHSYIEQGKKCRYCDAVFHERYALIQHQKSHKNEKRFKCDQCD
YACRQERHMIMHKRTHTGEKPYACSHCDKTFRQKQLLDMHFKRYHDPNFVPAAFVCSKCGKTFT
RRNTMARHADNCAGGSGSGS**GKPIPNPLLGLDST**

3. 11ZFs+C-terminus-V5 (N-terminus deleted CTCF)
MEGFQCELCSYTCPRRSNLDRHMKSHTDERPHKCHLCGRAFRTVTLLRNHLNTHTGTRPHKCPD
CDMAFVTSGELVRHRRYKHTHEKPFKCSMCDYASVEVSKLKRHIRSHTGERPFQCSLCSYASRDT
YKLKRHMRTHSGEKPYECYICHARFTQSGTMKMHILQKHTENVAKFHCPHCDTVIARKSDLGVH
LRKQHSYIEQGKKCRYCDAVFHERYALIQHQKSHKNEKRFKCDQCDYACRQERHMIMHKRTHTG
EKPYACSHCDKTFRQKQLLDMHFKRYHDPNFVPAAFVCSKCGKTFTRRNTMARHADNCAGPDG
VEGENGGETKKSKRGRKRKMRSKKEDSSDSENAEPDLDDNEDEEEPAVEIEPEPEPQPVTPAPPPA
KKRRGRPPGRTNQPKQNQPTAIIQVEDQNTGAIENIIVEVKKEPDAEPAEGEEEEAQPAATDAPNG
DLTPEMILSMMDRGSGSGS**GKPIPNPLLGLDST**

4. CTCF with AZFs-V5 (CTCF ZFs replaced with Artificial Zinc Fingers (AZF))
MEGDAVEAIVEESETFIKGKERKTYQRRREGGQEEDACHLPQNQTDGGEVVQDVNSSVQM
VMMEQLDPTLLQMKTEVMEGTVAPEAEAAVDDTQIITLQVVNMEEQPINIGELQLVQVPVP
VTVPVATTSVEELQGAYENEVSKEGLAESEPMICHTLPLPEGFQVVKVGANGEVETLEQGEL
PPQEDPSWQKDPDYQPPAKKTKKTKKSKLRYTEEGKDVDVSVYDFEEEQQEGLLSEVNAEK
VVGNMKPPKPTKIKKKGVKKTYACPVESCDRRFSDRSHLTRHIRIHTGQKPFQCRICMRNFS
RSDHLTRHIRTHTGEKPFACDICGRKFAQSSDLTRHTKIHLRPDGVEGENGGETKKSKRGRKR
KMRSKKEDSSDSENAEPDLDDNEDEEEPAVEIEPEPEPQPVTPAPPPAKKRRGRPPGRTNQPKQNQ
PTAIIQVEDQNTGAIENIIVEVKKEPDAEPAEGEEEEAQPAATDAPNGDLTPEMILSMMDRGSGSGS
GKPIPNPLLGLDST

5. BORIS-V5
MAATEISVLSEQFTKIKELELMPEKGLKEEEKDGVCREKDHRSPSELEAERTSGAFQDSVLE
EEVELVLAPSEESEKYILTLQTVHFTSEAVELQDMSLLSIQQQEGVQVVVQQPGPGLLWLEE
GPRQSLQQCVAISIQQELYSPQEMEVLQFHALEENVMVASEDSKLAVSLAETTGLIKLEEEQE
KNQLLAERTKEQLFFVETMSGDERSDEIVLTVSNSNVEEQEDQPTAGQADAEKAKSTKNQR
KTKGAKGTFHCDVCMFTSSRMSSFNRHMKTHTSEKPHLCHLCLKTFRTVTLLRNHVNTHTGTRP
YKCNDCNMAFVTSGELVRHRRYKHTHEKPFKCSMCKYASVEASKLKRHVRSHTGERPFQCCQCS
YASRDTYKLKRHMRTHSGEKPYECHICHTRFTQSGTMKIHILQKHGENVPKYQCPHCATIIARKSD
LRVHMRNLHAYSAAELKCRYCSAVFHERYALIQHQKTHKNEKRFKCKHCSYACKQERHMTAHIR
THTGEKPFTCLSCNKCFRQKQLLNAHFRKYHDANFIPTVYKCSKCGKGFSRWINLHRHSEKCGSG
EAKSAASGKGRRTRKRKQTILKEATKGQKEAAKGWKEAANGDEAAAEEASTTKGEQFPGEMFPV
ACRETTARVKEEVDEGVTCEMLLNTMDKGSGSGS**GKPIPNPLLGLDST**

6. BORIS with CTCF ZFs-V5 (Chimera1)
MAATEISVLSEQFTKIKELELMPEKGLKEEEKDGVCREKDHRSPSELEAERTSGAFQDSVLE
EEVELVLAPSEESEKYILTLQTVHFTSEAVELQDMSLLSIQQQEGVQVVVQQPGPGLLWLEE
GPRQSLQQCVAISIQQELYSPQEMEVLQFHALEENVMVASEDSKLAVSLAETTGLIKLEEEQE
KNQLLAERTKEQLFFVETMSGDERSDEIVLTVSNSNVEEQEDQPTAGQADAEKAKSTKNQR
KTKGAKGTFQCELCSYTCPRRSNLDRHMKSHTDERPHKCHLCGRAFRTVTLLRNHLNTHTGTRP
HKCPDCDMAFVTSGELVRHRRYKHTHEKPFKCSMCDYASVEVSKLKRHIRSHTGERPFQCSLCSY
ASRDTYKLKRHMRTHSGEKPYECYICHARFTQSGTMKMHILQKHTENVAKFHCPHCDTVIARKS
DLGVHLRKQHSYIEQGKKCRYCDAVFHERYALIQHQKSHKNEKRFKCDQCDYACRQERHMIMH
KRTHTGEKPYACSHCDKTFRQKQLLDMHFKRYHDPNFVPAAFVCSKCGKTFTRRNTMARHADN
CAGGEAKSAASGKGRRTRKRKQTILKEATKGQKEAAKGWKEAANGDEAAAEEASTTKGEQFPG
EMFPVACRETTARVKEEVDEGVTCEMLLNTMDKGSGSGS**GKPIPNPLLGLDST**

7. BORIS with the N-terminus of CTCF-V5 (Chimera2):
MEGDAVEAIVEESETFIKGKERKTYQRRREGGQEEDACHLPQNQTDGGEVVQDVNSSVQM
VMMEQLDPTLLQMKTEVMEGTVAPEAEAAVDDTQIITLQVVNMEEQPINIGELQLVQVPVP
VTVPVATTSVEELQGAYENEVSKEGLAESEPMICHTLPLPEGFQVVKVGANGEVETLEQGEL
PPQEDPSWQKDPDYQPPAKKTKKTKKSKLRYTEEGKDVDVSVYDFEEEQQEGLLSEVNAEK
VVGNMKPPKPTKIKKKGVKKTFQCDVCMFTSSRMSSFNRHMKTHTSEKPHLCHLCLKTFRTVT
LLRNHVNTHTGTRPYKCNDCNMAFVTSGELVRHRRYKHTHEKPFKCSMCKYASVEASKLKRHV
RSHTGERPFQCCQCSYASRDTYKLKRHMRTHSGEKPYECHICHTRFTQSGTMKIHILQKHGENVP
KYQCPHCATIIARKSDLRVHMRNLHAYSAAELKCRYCSAVFHERYALIQHQKTHKNEKRFKCKH
CSYACKQERHMTAHIRTHTGEKPFTCLSCNKCFRQKQLLNAHFRKYHDANFIPTVYKCSKCGKGF
SRWINLHRHSEKCGSGEAKSAASGKGRRTRKRKQTILKEATKGQKEAAKGWKEAANGDEAAAE
EASTTKGEQFPGEMFPVACRETTARVKEEVDEGVTCEMLLNTMDKGSGSGS**GKPIPNPLLGLDS**
T

8. BORIS with the N-terminus of CTCF plus the first 2 ZFs of CTCF and the linkers flanking ZF7 of CTCF (Chimera3):
MEGDAVEAIVEESETFIKGKERKTYQRRREGGQEEDACHLPQNQTDGGEVVQDVNSSVQM
VMMEQLDPTLLQMKTEVMEGTVAPEAEAAVDDTQIITLQVVNMEEQPINIGELQLVQVPVP
VTVPVATTSVEELQGAYENEVSKEGLAESEPMICHTLPLPEGFQVVKVGANGEVETLEQGEL
PPQEDPSWQKDPDYQPPAKKTKKTKKSKLRYTEEGKDVDVSVYDFEEEQQEGLLSEVNAEK
VVGNMKPPKPTKIKKKGVKKTFQCELCSYTCPRRSNLDRHMKSHTDERPHKCHLCGRAFRTVT
LLRNHVNTHTGTRPYKCNDCNMAFVTSGELVRHRRYKHTHEKPFKCSMCKYASVEASKLKRHV

RSHTGERPFQCCQCSYASRDTYKLKRHMRTHSGEKPYECHICHTRFTQSGTMKIHILQKHTENVA
KFHCPHCATIIARKSDLRVHMRNLHSYIEQGKKCRYCSAVFHERYALIQHQKTHKNEKRFKCKHC
SYACKQERHMTAHIRTHTGEKPFTCLSCNKCFRQKQLLNAHFRKYHDANFIPTVYKCSKCGKGFS
RWINLHRHSEKCGSGEAKSAASGKGRRTRKRKQTILKEATKGQKEAAKGWKEAANGDEAAAEE
ASTTKGEQFPGEMFPVACRETTARVKEEVDEGVTCEMLLNTMDK

9. CTCF with BORIS ZFs-V5 (Chimera4)
MEGDAVEAIVEESETFIKGKERKTYQRRREGGQEEDACHLPQNQTDGGEVVQDVNSSVQM
VMMEQLDPTLLQMKTEVMEGTVAPEAEAAVDDTQIITLQVVNMEEQPINIGELQLVQVPVP
VTVPVATTSVEELQGAYENEVSKEGLAESEPMICHTLPLPEGFQVVKVGANGEVETLEQGEL
PPQEDPSWQKDPDYQPPAKKTKKTKKSKLRYTEEGKDVDVSVYDFEEEQQEGLLSEVNAEK
VVGNMKPPKPTKIKKKGVKKTFQCDVCMFTSSRMSSFNRHMKTHTSEKPHLCHLCLKTFRTVT
LLRNHVNTHTGTRPYKCNDCNMAFVTSGELVRHRRYKHTHEKPFKCSMCKYASVEASKLKRHV
RSHTGERPFQCCQCSYASRDTYKLKRHMRTHSGEKPYECHICHTRFTQSGTMKIHILQKHGENVP
KYQCPHCATIIARKSDLRVHMRNLHAYSAAELKCRYCSAVFHERYALIQHQKTHKNEKRFKCKH
CSYACKQERHMTAHIRTHTGEKPFTCLSCNKCFRQKQLLNAHFRKYHDANFIPTVYKCSKCGKGF
SRWINLHRHSEKCGSPDGVEGENGGETKKSKRGRKRKMRSKKEDSSDSENAEPDLDDNEDEEEPA
VEIEPEPEPQPVTPAPPPAKKRRGRPPGRTNQPKQNQPTAIIQVEDQNTGAIENIIVEVKKEPDAEPA
EGEEEEAQPAATDAPNGDLTPEMILSMMDRGSGSGS**GKPIPNPLLGLDST**

10. CTCFdelta2ZFs-V5 (deltaZFs1,2-CTCF – CTCF with the two first 2 ZFs deleted)
MEGDAVEAIVEESETFIKGKERKTYQRRREGGQEEDACHLPQNQTDGGEVVQDVNSSVQM
VMMEQLDPTLLQMKTEVMEGTVAPEAEAAVDDTQIITLQVVNMEEQPINIGELQLVQVPVP
VTVPVATTSVEELQGAYENEVSKEGLAESEPMICHTLPLPEGFQVVKVGANGEVETLEQGEL
PPQEDPSWQKDPDYQPPAKKTKKTKKSKLRYTEEGKDVDVSVYDFEEEQQEGLLSEVNAEK
VVGNMKPPKPTKIKKKGVKKTFQCPDCDMAFVTSGELVRHRRYKHTHEKPFKCSMCDYASVE
VSKLKRHIRSHTGERPFQCSLCSYASRDTYKLKRHMRTHSGEKPYECYICHARFTQSGTMKMHIL
QKHTENVAKFHCPHCDTVIARKSDLGVHLRKQHSYIEQGKKCRYCDAVFHERYALIQHQKSHKN
EKRFKCDQCDYACRQERHMIMHKRTHTGEKPYACSHCDKTFRQKQLLDMHFKRYHDPNFVPAA
FVCSKCGKTFTRRNTMARHADNCAGPDGVEGENGGETKKSKRGRKRKMRSKKEDSSDSENAEP
DLDDNEDEEEPAVEIEPEPEPQPVTPAPPPAKKRRGRPPGRTNQPKQNQPTAIIQVEDQNTGAIENII
VEVKKEPDAEPAEGEEEEAQPAATDAPNGDLTPEMILSMMDRGSGSGS**GKPIPNPLLGLDST**

11. ChimeraAZF-V5 (CTCF ZFs 3-11 replaced with Artificial Zinc Fingers (AZF)
MEGDAVEAIVEESETFIKGKERKTYQRRREGGQEEDACHLPQNQTDGGEVVQDVNSSVQM
VMMEQLDPTLLQMKTEVMEGTVAPEAEAAVDDTQIITLQVVNMEEQPINIGELQLVQVPVP
VTVPVATTSVEELQGAYENEVSKEGLAESEPMICHTLPLPEGFQVVKVGANGEVETLEQGEL
PPQEDPSWQKDPDYQPPAKKTKKTKKSKLRYTEEGKDVDVSVYDFEEEQQEGLLSEVNAEK
VVGNMKPPKPTKIKKKGVKKTFQCELCSYTCPRRSNLDRHMKSHTDERPHKCHLCGRAFRTVT
LLRNHLNTHTGTRP**YACPVESCDRRFSDRSHLTRHIRIHTGQKPFQCRICMRNFSRSDHLTRHI**
RTHTGEKPFACDICGRKFAQSSDLTRHTKIHLRPDGVEGENGGETKKSKRGRKRKMRSKKEDS
SDSENAEPDLDDNEDEEEPAVEIEPEPEPQPVTPAPPPAKKRRGRPPGRTNQPKQNQPTAIIQVEDQ
NTGAIENIIVEVKKEPDAEPAEGEEEEAQPAATDAPNGDLTPEMILSMMDRGSGSGS**GKPIPNPLL**
GLDST

12. N1 mutant of CTCF (1-30aa replaced by BORIS aa sequence)
MAATEISVLSEQFTKIKELELMPEKGLKEEGGQEEDACHLPQNQTDGGEVVQDVNSSVQMV
MMEQLDPTLLQMKTEVMEGTVAPEAEAAVDDTQIITLQVVNMEEQPINIGELQLVQVPVPV
TVPVATTSVEELQGAYENEVSKEGLAESEPMICHTLPLPEGFQVVKVGANGEVETLEQGELP

PQEDPSWQKDPDYQPPAKKTKKTKKSKLRYTEEGKDVDVSVYDFEEEQQEGLLSEVNAEKV
VGNMKPPKPTKIKKKGVKKTFQCELCSYTCPRRSNLDRHMKSHTDERPHKCHLCGRAFRTVTL
LRNHLNTHTGTRPHKCPDCDMAFVTSGELVRHRRYKHTHEKPFKCSMCDYASVEVSKLKRHIRS
HTGERPFQCSLCSYASRDTYKLKRHMRTHSGEKPYECYICHARFTQSGTMKMHILQKHTENVAKF
HCPHCDTVIARKSDLGVHLRKQHSYIEQGKKCRYCDAVFHERYALIQHQKSHKNEKRFKCDQCD
YACRQERHMIMHKRTHTGEKPYACSHCDKTFRQKQLLDMHFKRYHDPNFVPAAFVCSKCGKTFT
RRNTMARHADNCAGPDGVEGENGGETKKSKRGRKRKMRSKKEDSSDSENAEPDLDDNEDEEEP
AVEIEPEPEPQPVTPAPPPAKKRRGRPPGRTNQPKQNQPTAIIQVEDQNTGAIENIIVEVKKEPDAEP
AEGEEEEAQPAATDAPNGDLTPEMILSMMDRGSGSGS**GKPIPNPLLGLDST**

13. N2 mutant of CTCF (1-87aa replaced by BORIS aa sequence)
MAATEISVLSEQFTKIKELELMPEKGLKEEEKDGVCREKDHRSPSELEAERTSGAFQDSVLE
EEVELVLAPSEESEKYILTLQTVHFAAVDDTQIITLQVVNMEEQPINIGELQLVQVPVPVTVPV
ATTSVEELQGAYENEVSKEGLAESEPMICHTLPLPEGFQVVKVGANGEVETLEQGELPPQED
PSWQKDPDYQPPAKKTKKTKKSKLRYTEEGKDVDVSVYDFEEEQQEGLLSEVNAEKVVGN
MKPPKPTKIKKKGVKKTFQCELCSYTCPRRSNLDRHMKSHTDERPHKCHLCGRAFRTVTLLRN
HLNTHTGTRPHKCPDCDMAFVTSGELVRHRRYKHTHEKPFKCSMCDYASVEVSKLKRHIRSHTG
ERPFQCSLCSYASRDTYKLKRHMRTHSGEKPYECYICHARFTQSGTMKMHILQKHTENVAKFHCP
HCDTVIARKSDLGVHLRKQHSYIEQGKKCRYCDAVFHERYALIQHQKSHKNEKRFKCDQCDYAC
RQERHMIMHKRTHTGEKPYACSHCDKTFRQKQLLDMHFKRYHDPNFVPAAFVCSKCGKTFTRRN
TMARHADNCAGPDGVEGENGGETKKSKRGRKRKMRSKKEDSSDSENAEPDLDDNEDEEEPAVEI
EPEPEPQPVTPAPPPAKKRRGRPPGRTNQPKQNQPTAIIQVEDQNTGAIENIIVEVKKEPDAEPAEGE
EEEAQPAATDAPNGDLTPEMILSMMDRGSGSGS**GKPIPNPLLGLDST**

14. N3 mutant of CTCF (90-186aa replaced by BORIS aa sequence)
MEGDAVEAIVEESETFIKGKERKTYQRRREGGQEEDACHLPQNQTDGGEVVQDVNSSVQM
VMMEQLDPTLLQMKTEVMEGTVAPEAEAAEAVELQDMSLLSIQQQEGVQVVVQQPGPGLL
WLEEGPRQSLQQCVAISIQQELYSPQEMEVLQFHALEENVMVASEDSKLAVSLAETTGLIKL
EEEQEDPSWQKDPDYQPPAKKTKKTKKSKLRYTEEGKDVDVSVYDFEEEQQEGLLSEVNAE
KVVGNMKPPKPTKIKKKGVKKTFQCELCSYTCPRRSNLDRHMKSHTDERPHKCHLCGRAFRTV
TLLRNHLNTHTGTRPHKCPDCDMAFVTSGELVRHRRYKHTHEKPFKCSMCDYASVEVSKLKRHI
RSHTGERPFQCSLCSYASRDTYKLKRHMRTHSGEKPYECYICHARFTQSGTMKMHILQKHTENVA
KFHCPHCDTVIARKSDLGVHLRKQHSYIEQGKKCRYCDAVFHERYALIQHQKSHKNEKRFKCDQ
CDYACRQERHMIMHKRTHTGEKPYACSHCDKTFRQKQLLDMHFKRYHDPNFVPAAFVCSKCGK
TFTRRNTMARHADNCAGPDGVEGENGGETKKSKRGRKRKMRSKKEDSSDSENAEPDLDDNEDE
EEPAVEIEPEPEPQPVTPAPPPAKKRRGRPPGRTNQPKQNQPTAIIQVEDQNTGAIENIIVEVKKEPD
AEPAEGEEEEAQPAATDAPNGDLTPEMILSMMDRGSGSGS**GKPIPNPLLGLDST**

15. N4 mutant of CTCF (187-265aa replaced by BORIS aa sequence)
MEGDAVEAIVEESETFIKGKERKTYQRRREGGQEEDACHLPQNQTDGGEVVQDVNSSVQM
VMMEQLDPTLLQMKTEVMEGTVAPEAEAAVDDTQIITLQVVNMEEQPINIGELQLVQVPVP
VTVPVATTSVEELQGAYENEVSKEGLAESEPMICHTLPLPEGFQVVKVGANGEVETLEQGEL
PPQGLIKLEEEQEKNQLLAERTKEQLFFVETMSGDERSDEIVLTVSNSNVEEQEDQPTAGQA
DAEKAKSTKNQRKTKGAKGTFQCELCSYTCPRRSNLDRHMKSHTDERPHKCHLCGRAFRTVTL
LRNHLNTHTGTRPHKCPDCDMAFVTSGELVRHRRYKHTHEKPFKCSMCDYASVEVSKLKRHIRS
HTGERPFQCSLCSYASRDTYKLKRHMRTHSGEKPYECYICHARFTQSGTMKMHILQKHTENVAKF
HCPHCDTVIARKSDLGVHLRKQHSYIEQGKKCRYCDAVFHERYALIQHQKSHKNEKRFKCDQCD
YACRQERHMIMHKRTHTGEKPYACSHCDKTFRQKQLLDMHFKRYHDPNFVPAAFVCSKCGKTFT

RRNTMARHADNCAGPDGVEGENGGETKKSKRGRKRKMRSKKEDSSDSENAEPDLDDNEDEEEP
AVEIEPEPEPQPVTPAPPPAKKRRGRPPGRTNQPKQNQPTAIIQVEDQNTGAIENIIVEVKKEPDAEP
AEGEEEEAQPAATDAPNGDLTPEMILSMMDRGSGSGS**GKPIPNPLLGLDST**

16. N5 mutant of CTCF (11aa mutations in the sites of poly(ADP)ribosylation replaced by BORIS aa sequence)
**MEGDAVEAIVEESETFIKGKERKTYQRRREGGQEEDACHLPQNQTDGGEVVQDVNSSVQM
VMMEQLDPTLLQMKTEVMEGTVAPEAEAAVDDTQIITLQVVNMEEQPINIGELQLVQVPVP
VTVPVATTSVEELQGAYENEVSKEGLAESEPMICHTLPLPAGFQVVKVGANGEVETLEQGAL
PPQEDPSWQKDPDYQPPAKKTKKTKKSKLRYTAAGKDVDVSVYAFAAAQQAGLLSAVNAA
KVVGNMKPPKPTKIKKKGVKKT**FQCELCSYTCPRRSNLDRHMKSHTDERPHKCHLCGRAFRTV
TLLRNHLNTHTGTRPHKCPDCDMAFVTSGELVRHRRYKHTHEKPFKCSMCDYASVEVSKLKRHI
RSHTGERPFQCSLCSYASRDTYKLKRHMRTHSGEKPYECYICHARFTQSGTKMHILQKHTENVAK
FHCPHCDTVIARKSDLGVHLRKQHSYIEQGKKCRYCDAVFHERYALIQHQKSHKNEKRFKCDQC
DYACRQERHMIMHKRTHTGEKPYACSHCDKTFRQKQLLDMHFKRYHDPNFVPAAFVCSKCGKT
FTRRNTMARHADNCAGPDGVEGENGGETKKSKRGRKRKMRSKKEDSSDSENAEPDLDDNEDEE
EPAVEIEPEPEPQPVTPAPPPAKKRRGRPPGRTNQPKQNQPTAIIQVEDQNTGAIENIIVEVKKEPDA
EPAEGEEEEAQPAATDAPNGDLTPEMILSMMDRGSGSGS**GKPIPNPLLGLDST**

17. N6 mutant of CTCF (the N-terminus of CTCF truncated to the 79aa)
**MEDPSWQKDPDYQPPAKKTKKTKKSKLRYTEEGKDVDVSVYDFEEEQQEGLLSEVNAEKV
VGNMKPPKPTKIKKKGVKKT**FQCELCSYTCPRRSNLDRHMKSHTDERPHKCHLCGRAFRTVTL
LRNHLNTHTGTRPHKCPDCDMAFVTSGELVRHRRYKHTHEKPFKCSMCDYASVEVSKLKRHIRS
HTGERPFQCSLCSYASRDTYKLKRHMRTHSGEKPYECYICHARFTQSGTMKMHILQKHTENVAKF
HCPHCDTVIARKSDLGVHLRKQHSYIEQGKKCRYCDAVFHERYALIQHQKSHKNEKRFKCDQCD
YACRQERHMIMHKRTHTGEKPYACSHCDKTFRQKQLLDMHFKRYHDPNFVPAAFVCSKCGKTFT
RRNTMARHADNCAGPDGVEGENGGETKKSKRGRKRKMRSKKEDSSDSENAEPDLDDNEDEEEP
AVEIEPEPEPQPVTPAPPPAKKRRGRPPGRTNQPKQNQPTAIIQVEDQNTGAIENIIVEVKKEPDAEP
AEGEEEEAQPAATDAPNGDLTPEMILSMMDRGSGSGS**GKPIPNPLLGLDST**

Fig. S25. Amino acid (aa) sequences of Open Reading Frames (ORFs) of all ectopically expressed constructs in this study (the amino acid sequences are numbered in order of their appearance in the study. Aa sequences highlighted in red, blue, green, black and purple belong to CTCF, BORIS, AZF, flexible linker and V5-tag peptides, respectively. CTCF and BORIS ZFs are underlined, the N-terminus of both proteins is in bold. In the sequence #16, the amino acids that shown to be poly(ADP)ribosylated are replaced by alanine (**A,** highlighted by black color and underlined).

SI Materials and Methods

Plasmid constructs

All ectopically-expressed constructs described in this study were generated on a backbone of retroviral vector: pMy-MouseCTCFbiotag-T2A-mOrange (Addgene plasmid # 50564; http://n2t.net/addgene:50564), first described in (1). Expression vectors were generated by subcloning of Open Reading Frames (ORFs) (listed in Fig.S25) into the EcoRI and XhoI sites of pMy-MouseCTCFbiotag-T2A-mOrange plasmid, replacing the mouse CTCF ORF. The amino acid sequence of ORFs for all constructs is presented in Fig. S25. The ORFs were synthetized commercially by GenScript (https://www.genscript.com). The sequence of Artificial Zinc Fingers (AZF) (Fig. S25) has been described previously in (2) and kindly provided by Dr. Crossley. All chimeric constructs were verified by both a restriction enzyme digestion and sequencing. All ORFs were expressed from the LTR promoter, contained the C-terminal glycine-serine flexible linker (amino acid sequence: GSGSGS) followed by a V5-tag (amino acid sequence: GKPIPNPLLGLDST) for immunoprecipitation with anti-V5 antibody, followed by T2A sequence (self-cleaving peptides), followed by ORF of Orange Fluorescent Protein (OFP).

Retroviral transduction of CH12 cells

Retroviral supernatants were obtained by transfection of the retroviral vectors into the Plat-E packaging cell line (3). First, PlatE cells were plated at a density of 3 x 10^6 cells per 150mm plate. 18 hours later the cells were transiently transfected with 15 μg of plasmid DNA by using jetPEI DNA transfection reagent (Polyplus), following the manufacturer's recommendation. Supernatants were collected twice (48 and 72hr after transfection) and processed by centrifugation at 1,000 g for 5 min at 4°C to remove cellular debris, followed by ultrafiltration using 0.45μm PVDF filters (Millipore, Catalog no. SLHVR25LS). 2mL of undiluted viral supernatants, supplemented with 4 μg/mL Polybrene (Sigma-Aldrich, MO), were mixed with 1x10^6 CH12 cells and spun down at 2500 rpm for 2 h at 32°C. After centrifugation, the cells were incubated for 24h and cultured with fresh medium. 4 days after first infection, CH12 cells were sorted using a BD Fluorescence-Activated Cell Sorting (FACS) Aria (Becton Dickinson). The percentage of OFP positive cells before FACS sorting was 30%–50%. After FACS sorting and two-three weeks of cell culturing, the percentage of OFP+ cells was estimated as 80-90%.

Cell lines used in this study

K562 and MCF7 cell lines (obtained from ATCC) were grown in Dulbecco's modified Eagle medium (DMEM) supplemented with 10 % fetal calf serum and penicillin-streptomycin. CH12 LX B lymphoma cell line was maintained in RPMI 1640 supplemented with 10% FBS, 1% penicillin/streptomycin and 55 μM 2-β mercaptoethanol. CH12 wild type (wt) and mutant (mut) cells were described previously in (4) and obtained as a gift from Dr. Rafael Casellas (NIH, NIAMS). A retrovirus packaging cell line named Platinum-E (Plat-E), derived from the 293T cell line (3), was purchased from Cell Biolabs. Plat-E cells were cultured in DMEM supplemented with 10% heat-inactivated fetal bovine serum, 100U/ml penicillin, 100 mg/mL streptomycin, 10μg/mL blasticidin, and 1μg/mL puromycin at 37°C in 5% CO2–95%. The homozygous mutation in mut CH12 cells compared to wt CH12 cells was confirmed on both cDNA and genomic DNA level with the following primers: cDNA amplification (forward: 5’-GAAGCGCTTCAAGTGTGAC-3’, reverse 5’- CTTTGTCTCCCCTCCATTTTCC); genomic DNA amplification (forward:

5'-GGAGGCCTTTCTGACAAGCAGTTCTG-3', reverse 5'-CATAACAGGAGCCTGAGCCTTACC-3").

ChIP-seq

ChIP-seq was performed as described in (5). Briefly, $2x10^6$ asynchronously growing cells were crosslinked with 1% formaldehyde for 10 min at room temperature followed by quenching with 125 mM glycine for 10 min, washed twice with 1× phosphate buffered saline (PBS), and resuspended in chromatin immunoprecipitation (ChIP) lysis buffer (150 mM NaCl, 1 % Triton X-100, 0.1 % SDS, 20 mM Tris–HCl pH8.0, 2 mM EDTA). Chromatin was sheared to 200–500 bp and immunoprecipitated with 5 μg of antibody. Immunoprecipitated DNA was purified with QIAquick columns (ZymaResearch). DNA concentration was assessed with a Qubit4 (ThermoFisher) and 5–10 ng was used to generate sequencing libraries using a TruSeq ChIP Sample Preparation Kit (Illumina, Inc., USA).

Antibodies used in ChIP-seq

Anti-CTCF antibodies used in ChIP-seq experiments: Novus Biologicals (NB100-56494, NB100-947, NB500-194), Abcam ab70303; Bethyl Laboratories (A300-543A, A300-544A, A300-542A); Santa Cruz (SC-271514); Millipore (07-729) and custom-made mouse monoclonal CTCF antibodies described in (6). Custom-made BORIS antibodies used in ChIP-seq were described previously (7). Anti-V5-Tag (# R960-25, ThermoFisher) and anti-RAD21 (ab992, Abcam) antibodies were used for both ChIP-seq and Western analysis. The anti-NIPBL (Bethyl, A301-779A) antibody was used in ChIP-seq.

Bioinformatic analysis of ChIP-seq data

Single-end sequences were generated by the Illumina genome analyzer (36-60 bp reads) were aligned against either the human (build hg19) or mouse (build mm9) genome using the Bowtie program with the default parameters (8), except the sequence tags that mapped to more than one location in the genome were excluded from the analysis using the –m1 option. Peaks were called using Model-based Analysis for ChIP-seq (MACS2) using default parameters (https://github.com/taoliu/MACS). The ChIP-seq data were visualized using the Integrative Genomics Viewer (IGV) (9). The peak overlaps between ChIP-seq data sets were determined with the BedTools Suite (10). We defined peaks as overlapping if at least 1 bp of of each peak overlapped. The normalized tag density profiles were generated using the BedTools coverage option from the BedTools Suite (10), normalized to the number of mapped reads, and plotted in Microsoft Excel. The heatmaps and the average profiles of ChIP-Seq tag densities for different clusters were generated using the seqMINER 1.3.3 platform (11). We used k-means ranked method for clustering normalization. Position weight matrices were calculated using Multiple EM for Motif Elicitation (MEME) software (12). The sequences under the summit of ChIP-seq peaks were extended 100 bp upstream and downstream for motif discovery. We ran MEME with parameters (−mod oops -revcomp -w 40 or -w 20) to identify the long and short CTCF motifs considering both DNA strands. Genomic distribution of CTCF ChIP-seq peaks relative to reference genes was performed using the Cis-regulatory Element Annotation System (CEAS) (13). To call the genomic regions bound either by CTCF or RAD21 or by both proteins in the four cell lines (Fig.1C-D, F), we calculated CTCF and RAD21 ChIP-seq tag densities at each binding region. For this we combined CTCF and RAD21 binding sites into a composite set, extended the summit of peaks to 300 bp, and calculated either CTCF or RAD21 normalized ChIP-seq tag density at each binding region using BedTools

Coverage option. We classified the sites as "Cohesin-Non-CTCF" or "CTCF depleted of RAD21" if a difference in the tag density between the two factors was more than 3-fold at the binding region. To calculate the percent of cohesin (RAD21) occupancy at the lost CTCF sites in Fig. 7, we calculated RAD21 ChIP-seq tag densities (normalized to the number of mapped reads) mediated by the ectopic expression of either empty vector, FL-CTCF, chimeric or mutant constructs in mut CH12 cells. The RAD21 ChIP-seq tag density at the lost CTCF sites either followed by the ectopic expression of empty vector was taken as 0% or followed by the expression of FL-CTCF was taken as 100% cohesin occupancy. The percent of cohesin occupancy at the lost CTCF sites by chimeric and mutant proteins was calculated on the scale between 0% and 100%. In the case of chimeric proteins (Fig. 7B), we calculated RAD21 ChIP-seq tag density only at the lost CTCF sites that have a similar occupancy (ChIP-seq tag density) for FL-CTCF and the corresponding chimeric protein at these sites. All ChIP-seq data have been deposited in the Gene Expression Omnibus (GEO) repository with the following GEO accession number: GSE137216.

RNA-seq

The RNA sequencing library preparation and sequencing procedures were carried out according to Illumina protocols. FASTQ files were mapped to the UCSC Mouse reference (mm9) using TopHat2 (14) with the default parameter setting of 20 alignments per read and up to two mismatches per alignment. The aligned reads (BAM files) were analyzed with Cufflinks 2.0 to estimate transcript relative abundance using the UCSC reference annotated transcripts (mm9). The expression of each transcript was quantified as the number of reads mapping to a transcript divided by the transcript length in kilobases and the total number of mapped reads in millions (FPKM). Cuffdiff was applied to obtain the list of deregulated genes. Transcripts having more than 2-fold changes in their expression and p-value less than 0.005 were used for further analysis. RNA-seq data have been deposited in the GEO repository with the following accession number: GSE137216.

Hi-C

In situ Hi-C experiments were performed as previously described using the MboI restriction enzyme (15). The crosslinked pellets (1.5 million cells) were incubated and washed with 200 µL of lysis buffer (10 mM Tris-HCl pH 8.0, 10 mM NaCl, 0.2% Igepal CA630, 33 µL Protease Inhibitor (Sigma, P8340)) on ice, and then incubated in 50 µL of 0.5% SDS for 10 min at 62°C. After heating, 170 µL of 1.47% Triton X-100 was added and incubated for 15 min at 37˚C. To digest chromatin, 100 U MboI and 25 µL of 10X NEBuffer2 were added followed by overnight incubation at 37˚C.

The digested ends were filled and labeled with biotin by adding 37.5 µL of 0.4 mM biotin-14-dATP (Life Tech), 1.5 µL of 10 mM dCTP, 10 mM dTTP, 10 mM dGTP, and 8 µL of 5 U/µL Klenow (New England Biolabs) and incubating at 23°C for 60 minutes with shaking at 500 rpm on a thermomixer. Then the samples were mixed with 1x T4 DNA ligase buffer (New England Biolabs), 0.83% Triton X-100, 0.1 mg/mL BSA, 2000 U T4 DNA Ligase (New England Biolabs, M0202), and incubated for at 23°C for 4 hours to ligate the ends. After the ligation reaction, samples were resuspended in 550 µL 10 mM Tris-HCl, pH 8.0. To reverse the crosslinks, 50 µL of 20 mg/mL Proteinase K (New England Biolabs) and 57 µL of 10% SDS were mixed with the samples, and incubated at 55°C for 30 minutes, and then 67 µL of 5 M NaCl were added followed by overnight incubation at 68°C. After cooling at room temperature, 0.8X Ampure (Beckman-Coulter) purification was performed, and the samples were sonicated to a mean fragment length of

400 bp using Covaris M220. Two rounds of Ampure (Beckman-Coulter) beads purification was performed for size selection.

Biotin-labeled DNA was purified using Dynabeads MyOne T1 Streptavidin beads (Invitrogen). The beads were washed with 400 μL of 1x Tween Wash Buffer (5 mM Tris-HCl pH 7.5, 0.5 mM EDTA, 1 M NaCl, 0.05% Tween-20), and resuspended in 300 μL of 2x Binding Buffer (10 mM Tris-HCl pH 7.5, 1 mM EDTA, 2 M NaCl). The beads were added to samples and incubated for 15 minutes at room temperature. Then the beads were washed twice by adding 600 μL of 1x Tween Wash Buffer. Then the beads were equilibrated once in 100 μL 1x NEB T4 DNA ligase buffer (New England Biolabs) followed by removal of the supernatant using a magnetic rack. To repair the fragmented ends, the beads were resuspended in 100 μL of the following: 88 μL 1X NEB T4 DNA ligase buffer (New England Biolabs, B0202), 2 μL of 25 mM dNTP mix, 5 μL of 10 U/μL T4 PNK (New England Biolabs), 4 μL of 3 U/μL NEB T4 DNA Polymerase (New England Biolabs), 1 μL of 5 U/μL Klenow (New England Biolabs). The beads were incubated for 30 minutes at room temperature. The beads were washed twice by adding 600 μL of 1x Tween Wash Buffer. To add a dA-tail, the beads were resuspended in 100 μL of the following: 90 μL of 1X NEBuffer2, 5 μL of 10 mM dATP, and 5 μL of 5 U/μL Klenow (exo-) (New England Biolabs). The beads were incubated for 30 minutes at 37˚C. The beads were washed twice by adding 600 μL of 1x Tween Wash Buffer. Following the washes, the beads were equilibrated once in 100 μL 1x NEB Quick Ligation Reaction Buffer (New England Biolabs) and the supernatants were removed using a magnetic rack. The beads were then resuspended in 50 μL 1x NEB Quick Ligation Reaction Buffer. To ligate adapters, 2 μL of NEB DNA Quick Ligase (New England Biolabs) and 3 μL of Illumina Indexed adapter were added to the beads and incubated for 15 minutes at room temperature. The supernatant was removed and the beads were washed twice by adding 600 μL of 1x Tween Wash Buffer. Then the beads were resuspended once in 100 μL 10 mM Tris-HCl, pH 8.0, followed by removal of the supernatant and resuspension again in 50 μL 10 mM Tris-HCl, pH 8.0. After deciding an optimal PCR cycle number using KAPA DNA Quantification kit (Kapa Biosystems), 6 cycles of PCR amplification were performed with the following reaction mixture: 10 μL Phusion HF Buffer (New England Biolabs), 3.125 μL 10 μM TruSeq Primer 1, 3.125 μL 10 μM TruSeq Primer 2, 1 μL 10 mM dNTPs, 0.5 μL Fusion HotStartII, 20.75 μL ddH20, 11.5 μL Bead-bound Hi-C library. PCR products were subjected to a final purification using AMPure beads (Beckman-Coulter) and were eluted in 30 μL 10 mM Tris-HCl, pH 8.0. Libraries were sequenced on the Illumina HiSeq 4000 platform. Hi-C data have been deposited in the GEO repository with the following accession number: GSE136122.

Hi-C data analysis

Hi-C reads (paired end, 50 bases) were aligned against the mm9 genome using BWA-mem (16). PCR duplicate reads were removed using Picard MarkDuplicates. We used juicebox (17) to create hic file with -q 30 -f options and to visualize Hi-C data. The aggregate analysis of chromatin loops was performed using APA (17) with default parameters and 10 kb resolution. The list of chromatin loops identified in wild type CH12 were downloaded from (15).

Published next-generation experiments used in this study

ChIP-seq data for CTCF and RAD21 in K562 and HEPG2 cell lines used in the study: GSE32465 (18), GSE38163 (19), GSE30263(20), GSE25021 (21), GSE36030 (19),

GSM1010820 (19), GSM749733 (19), GSM822311 (19), GSM935407 (18). Other ChIP-seq data used in this study: GSE76893 for NIPBL ChIP-seq in HEPG2 and MCF7 cells (22); GSM1979785 for NIPBL in mES cells (23); GSM1187116 for ESR1 in MCF7 cells (24); GSE25021 for CEPBA in HEPG2 (21), GSM1910644 for OCT4 in mES (25), GSM2418860 and GSM2418859 for CTCF and RAD21 in mES, respectively (26); GSM2587373 for ChIA-PET in CH12 wt cells (4); GSE63525 for Hi-C in CH12 wt cells (15). ChIP-seq for Ets1 (GSM1003774), CoRest (GSM1003786), HCFC1 (GSM1003795) (19).

Nuclear extract (NUN) preparation

Nuclei were purified from K562 cells by homogenization in a 1.9 M final concentration sucrose solution, followed by pelleting once through a 2 M sucrose cushion (27). DNA concentrations were determined by spectrophotometric absorbance at 260 nm, and nuclei were used either directly or flash-frozen in nuclear storage buffer (NSB: 20 mM Tris-Cl (pH 7.9), 75 mM NaCl, 0.5 mM EDTA, 0.85 mM DTT, 0.125 mM PMSF, 50% glycerol) and stored in liquid nitrogen. Thawed or fresh nuclei in NSB were concentrated to 25 mg/mL of DNA content, which is essentially a nuclear slurry, in a 1.5-mL plastic microcentrifuge tube or in a glass Corex tube. After resuspension by gentle agitation, nine volumes of a 1.1x NUN solution was added to give final concentrations of 1 M urea, 0.3 M NaCl, 1% Nonidet-P40, 25 mM HEPES (pH 7.6), and 1 mM DTT (28). After vigorous vortex agitation for 5 sec, the tubes were left on ice for 15 min, during which time a string-like precipitate of chromatin and associated structures appeared. The chromatin precipitate was sedimented by centrifugation (10 min at 4°C in a microcentrifuge), allowing nearly quantitative removal of the supernatant. Glycerol was added to the supernatant to a final concentration of 10%, and small aliquots were flash-frozen and stored in liquid nitrogen.

Sephacryl-300 chromatography

2 mL K562 NUN nuclear extract was loaded to Sephacryl 300 column and separated in 1xNUN. 2 mL fractions were collected and 20 μL of each fraction was analyzed by Western blot.

Western blotting

Protein extracts were prepared with RIPA Lysis buffer (Millipore) containing 50 mM Tris–HCl, pH 7.4, 1 % Nonidet P-40, 0.25 % sodium deoxycholate, 500 mM NaCl, 1 mM EDTA, 1× protease inhibitor cocktail (Roche Applied Science). The protein extracts were resolved by SDS-PAGE, transferred to a PVDF membrane, and incubated with the indicated antibodies. Detections were performed using ECL reagents. Primary antibodies used in Western experiments: anti-CTCF N-terminal (custom-made mouse monoclonal CTCF antibodies described in (6), and anti-CTCF C-terminal (Santa Cruz (SC-271514); custom-made BORIS antibodies previously described in (7); anti-V5-Tag (# R960-25, ThermoFisher), anti-RAD21 (ab992, Abcam) antibodies.

Co-immunoprecipitation assay

For co-immunoprecipitation reactions, 20 μL of DiaMag protein G-coated magnetic beads (Diagenode) were incubated with either 5 μg of anti-CTCF (sc-271514, Lot#2016, Santa Cruz Biotechnology), anti-YY1 (sc-7341, Lot#E039, Santa Cruz Biotechnology), anti-PARP1 (#39559, Lot#10618002, Active Motif), anti-cMYC (ab152146, Abcam) anti-V5 Tag (46-0705, Lot#1949337, Invitrogen), anti-RAD21 (ab992, Lot#GR3216371-3, Abcam), anti-SMC3 (ab9263, Lot#GR290533-8, Abcam), anti-SA2 (ab229681, Lot#GR3230882-2, Abcam), or anti-SMC1 (A300-055A, Bethyl) antibodies in PBS

supplied with 0.5% of bovine serum albumin (BSA) for 2 h at room temperature under a constant rotation. After 2 h of incubation, the beads were washed three times with PBS + 0.5% of BSA. Protein extracts of wt CH12 cells were prepared with RIPA Lysis buffer (Millipore) containing 50 mM Tris–HCl, pH 7.4, 1 % Nonidet P-40, 0.25 % sodium deoxycholate, 500 mM NaCl, 1 mM EDTA, 1× protease inhibitor cocktail (Roche Applied Science). Next, the antibody-bound beads were incubated with 1.5 mg of protein extracts in the presence of ethidium bromide (100 μg/μL) overnight at 4°C with constant rotation. Of note, the protein extracts were pre-cleared with 30 μL of DiaMag protein G-coated magnetic beads for 2 h under a constant rotation at 4°C. The immunoprecipitates were collected using a magnetic rack, washed five times with PBS+0.5 % BSA, dissolved in 1X LDS Sample buffer (Invitrogen) supplemented with DTT (50 mM final concentration), and boiled for 5 min at 90°C. Immunoprecipitated samples were resolved by SDS-PAGE, transferred to a PVDF membrane, and incubated with the indicated antibodies. Detections were performed using ECL reagents.

Electrophoretic mobility shift assay (EMSA)

A DNA fragment encompassing a 187 bp-long sequence of the human *p53* promoter, derived from a CTCF ChIP-seq peak mapped in K562 cells (5), was synthesized by PCR with the primers (Forward: 5' GAGCTCGATAATAAATATTTTTTGAATGAG 3' and Reverse: 5' CTGAACGCTTCTATCTTGGCGAGAAGC 3'). The PCR fragment was labeled using 32P-γ-ATP with T4 polynucleotide kinase (New England, Biolabs) as described in (6). NUN protein extract (1μl)–DNA complexes were incubated for 1 h at room temperature in binding buffer containing 25 mM Tris pH 7.4, 0.1 mM ZnSO4, 5 mM MgCl2, 5 % Nonidet P-40 in PBS, 0.25 mM mercaptoethanol, 10 % glycerol and 0.5 μg of poly dI-dC. For supershift analysis, either 2 μg of anti-CTCF (sc-271514, Santa Cruz Biotechnology), anti-RAD21 (ab992, Abcam) or anti-BORIS (custom-made (5)) antibodies were added for an additional 30 min incubation at room temperature. Protein–DNA complexes were separated from the unbound probe using 5 % native polyacrylamide gels (PAAG) and autoradiographed in 24-48 hours.

References

1. Nakahashi H, *et al.* (2013) A genome-wide map of CTCF multivalency redefines the CTCF code. *Cell Rep* 3(5):1678-1689.
2. Lim WF, *et al.* (2016) Directing an artificial zinc finger protein to new targets by fusion to a non-DNA-binding domain. *Nucleic Acids Res* 44(7):3118-3130.
3. Morita S, Kojima T, & Kitamura T (2000) Plat-E: an efficient and stable system for transient packaging of retroviruses. *Gene Ther* 7(12):1063-1066.
4. Vian L, *et al.* (2018) The Energetics and Physiological Impact of Cohesin Extrusion. *Cell* 173(5):1165-1178 e1120.
5. Pugacheva EM, *et al.* (2015) Comparative analyses of CTCF and BORIS occupancies uncover two distinct classes of CTCF binding genomic regions. *Genome Biol* 16:161.
6. Pugacheva EM, *et al.* (2005) Familial cases of point mutations in the XIST promoter reveal a correlation between CTCF binding and pre-emptive choices of X chromosome inactivation. *Hum Mol Genet* 14(7):953-965.

7. Suzuki T, *et al.* (2010) Expression of a testis-specific form of Gal3st1 (CST), a gene essential for spermatogenesis, is regulated by the CTCF paralogous gene BORIS. *Mol Cell Biol* 30(10):2473-2484.
8. Langmead B, Trapnell C, Pop M, & Salzberg SL (2009) Ultrafast and memory-efficient alignment of short DNA sequences to the human genome. *Genome Biol* 10(3):R25.
9. Robinson JT, *et al.* (2011) Integrative genomics viewer. *Nat Biotechnol* 29(1):24-26.
10. Quinlan AR & Hall IM (2010) BEDTools: a flexible suite of utilities for comparing genomic features. *Bioinformatics* 26(6):841-842.
11. Ye T, *et al.* (2011) seqMINER: an integrated ChIP-seq data interpretation platform. *Nucleic Acids Res* 39(6):e35.
12. Bailey TL, *et al.* (2009) MEME SUITE: tools for motif discovery and searching. *Nucleic Acids Res* 37(Web Server issue):W202-208.
13. Shin H, Liu T, Manrai AK, & Liu XS (2009) CEAS: cis-regulatory element annotation system. *Bioinformatics* 25(19):2605-2606.
14. Trapnell C, Pachter L, & Salzberg SL (2009) TopHat: discovering splice junctions with RNA-Seq. *Bioinformatics* 25(9):1105-1111.
15. Rao SS, *et al.* (2014) A 3D map of the human genome at kilobase resolution reveals principles of chromatin looping. *Cell* 159(7):1665-1680.
16. Li H & Durbin R (2010) Fast and accurate long-read alignment with Burrows-Wheeler transform. *Bioinformatics* 26(5):589-595.
17. Durand NC, *et al.* (2016) Juicebox Provides a Visualization System for Hi-C Contact Maps with Unlimited Zoom. *Cell Syst* 3(1):99-101.
18. Gertz J, *et al.* (2013) Distinct properties of cell-type-specific and shared transcription factor binding sites. *Mol Cell* 52(1):25-36.
19. Consortium EP (2012) An integrated encyclopedia of DNA elements in the human genome. *Nature* 489(7414):57-74.
20. Wang H, *et al.* (2012) Widespread plasticity in CTCF occupancy linked to DNA methylation. *Genome Res* 22(9):1680-1688.
21. Schmidt D, *et al.* (2010) A CTCF-independent role for cohesin in tissue-specific transcription. *Genome Res* 20(5):578-588.
22. Fournier M, *et al.* (2016) FOXA and master transcription factors recruit Mediator and Cohesin to the core transcriptional regulatory circuitry of cancer cells. *Sci Rep* 6:34962.
23. Busslinger GA, *et al.* (2017) Cohesin is positioned in mammalian genomes by transcription, CTCF and Wapl. *Nature* 544(7651):503-507.
24. Fletcher MN, *et al.* (2013) Master regulators of FGFR2 signalling and breast cancer risk. *Nat Commun* 4:2464.
25. Liu Z & Kraus WL (2017) Catalytic-Independent Functions of PARP-1 Determine Sox2 Pioneer Activity at Intractable Genomic Loci. *Mol Cell* 65(4):589-603 e589.
26. Hansen AS, Pustova I, Cattoglio C, Tjian R, & Darzacq X (2017) CTCF and cohesin regulate chromatin loop stability with distinct dynamics. *Elife* 6.
27. Tian JM & Schibler U (1991) Tissue-specific expression of the gene encoding hepatocyte nuclear factor 1 may involve hepatocyte nuclear factor 4. *Genes Dev* 5(12A):2225-2234.

28. Lavery DJ & Schibler U (1993) Circadian transcription of the cholesterol 7 alpha hydroxylase gene may involve the liver-enriched bZIP protein DBP. *Genes Dev* 7(10):1871-1884.
